# Whole Genome Sequencing of Infectious Bursal Disease Viruses Isolated from a Californian Outbreak Unravels the Underlying Virulence Markers and Highlights Positive Selection Incidence

**DOI:** 10.3390/v15102044

**Published:** 2023-10-03

**Authors:** Islam Nour, Julia R. Blakey, Sonsiray Alvarez-Narvaez, Sujit K. Mohanty

**Affiliations:** United States Department of Agriculture, Agricultural Research Service (USDA-ARS), US National Poultry Research Center, Athens, GA 30605, USA; islam.mohamed@usda.gov (I.N.); julia.blakey@usda.gov (J.R.B.); sonsiray.alvareznarvaez@usda.gov (S.A.-N.)

**Keywords:** IBDV, virulence, genotype, reassortant, recombination, positive selection

## Abstract

Outbreaks of the immunosuppressive infectious bursal disease (IBD) are frequently reported worldwide, despite the vaccination regimes. A 2009 Californian IBD outbreak caused by rA and rB isolates was described as very virulent (vv) IBD virus (IBDV); however, molecular factors beyond this virulence were not fully uncovered. Therefore, segments of both isolates were amplified, successfully cloned, whole genome sequenced by Next Generation Sequencing, genotyped, and the leading virulence factors were entirely investigated in terms of phylogenetic and amino acid analysis and protein modeling for positive selection orientation and interaction analysis. rA and rB isolates displayed the highest amino acid identity (97.84–100%) with Genotype 3 strains. Interestingly, rA and rB contained all virulence hallmarks of hypervariable (HVR), including 222A, 242I, 249Q, 256I, 284A, 286T, 294I, 299S, and 318G, as well as the serine-rich heptapeptide sequence. Moreover, we pinpointed the A3B2 genotype of rA and rB, predominant in non-reassortants, and we highlighted the absence of recombination events. Furthermore, gene-wise phylogenetic analysis showed the entire genes of rA and rB clustered with the vvIBDVs and emphasized their share in IBDV virulence. VP5 showed a virulence marker, MLSL (amino acid sequence). VP2 encountered three significant novel mutations apart from the HVR, including G163E in rA and Y173C and V178A in rB, all residing within interacting motifs. VP4 contained 168Y, 173N, 203S, and 239D characteristic for the vv phenotype. A235V mutation was detected at the dsRNA binding domain of VP3. In VP1, the TDN triplet and the mutation (V4I) were detected, characteristic of hypervirulence occurring at the N-terminus responsible for protein priming. Although selection analysis revealed seven sites, codon 222 was the only statistically significant selection site. The VP2 modeling of rA and rB highlighted great structure fitness, with 96.14% Ramachandran favored positioning including the 222A, i.e., not influencing the structure stability. The 222A was found to be non-interface surface residue, associated with no interaction with the attachment-mediated ligand motif. Our findings provide pivotal insights into the evolution and underlying virulence factors and will assist in the development of control strategies via sequence-based continuous monitoring for the early detection of novel vv strains.

## 1. Introduction

Infectious bursal disease (IBD) is an acute, highly contagious viral infection of young chickens, first discovered in 1960 in Gumboro, DE, USA, and is therefore also referred as Gumboro disease [1]. IBD is almost seen in every country around the world and represents a significant economic impact on the poultry industry because it causes high mortality in chickens 3–6 weeks of age, immunosuppression leading to vaccination failure, or secondary bacterial and viral infections. The disease is caused by the infectious bursal disease virus (IBDV), a member of the family Birnaviridae, genus Avibirnavirus. IBDV is a non-enveloped virus with icosahedral capsid symmetry encapsulating double-stranded RNA [2,3]. IBDV is characterized by its bipartite genome, which encompasses segments A and B. Segment A (3.4 kb) contains two partially overlapped open reading frames (ORFs) comprising the polyprotein (PP) (NH_2_-VP2 (38 kDa)-VP4 (28 kDa)-VP3 (32 kDa)-COOH), encoding a larger ORF and a smaller ORF-encoding protein VP5 (17 kDa) [4]. VP4 encodes the viral protease required for PP cleavage, yielding the two major structural proteins, VP2 and VP3. VP2, the viral capsid protein that mostly generates serotype-neutralizing antibodies, is a frequent target for IBDV molecular diagnosis and genotyping [5]. Segment B (2.8 kb) encodes the VP1 protein that functions as an RNA-dependent RNA polymerase. Sequence analysis of various IBDV pathotypes resulted in the identification of the VP2 hypervariable region (HVR) (amino acids (aa) positions 206 to 350) that contains the typical immunodominant epitopes required for the generation of protective antibodies [6,7,8]. HVR is also commonly used for genetic evolution analyses [3,9]. VP2-HVR is mostly hydrophobic, but it contains two major hydrophilic peaks, A and B, at aa 212–224 and 312–324, respectively. IBDV antigenic variations are frequent [8] and significant at these peaks to the extent that a single mutation at these loops could hamper IBD vaccine efficacy [9,10,11].

IBDV strains are antigenically classified into two distinct serotypes, 1 and 2, that can infect turkeys and chickens indistinctly. Still, the clinical disease is typically detected in IBDV serotype 1-infected chickens [12]. Moreover, IBDV serotype 1 can be classified according to its virulence into four pathotypes, which include classical virulent (Cl), antigenic variant (v), very virulent (vv), and attenuated strains. Initially, IBDVs of serotype 1 were regarded as the same antigenic type and termed a classic viruses. Thereafter, IBDV antigenic variants emerged in the United States [13]. These variants were characterized by their ability to overcome the acquired immunity in vaccinated chickens or flocks with maternal antibodies against the classical IBDVs [14]. Interestingly, the analysis of the sequencing data for VP2 revealed that there are characteristic amino acids for each of the different pathotypes (Cl, v, and vv). For instance, the aa P, Q, T, and G at 222, 249, 286, and 318, respectively, define the IBDV Cl strains, whereas the aa A, Q, T, and G denote the vv-IBDVs at the same positions [9,15]. On the contrary, aa 222T, 249K, 286I, and 318D are characteristic of the variant strains [16,17,18].

Ongoing surveillance programs of the circulating IBDV pathotypes are essential for reviewing the applied vaccination protocols and for enhancing control strategies. IBD surveillance generally involves the molecular characterization of new isolates and usually follows the same framework comprising periodic field sampling, IBD molecular detection, and sequencing the immunodominant region of the virus [5,19]. IBD sequencing data has been used to study IBDV evolution in different geographical locations and to report the detection of new antigenic variants [20,21], reassortants [22,23], recombinants [24], and distinct strains [25,26], which subsequently influenced the effectiveness of vaccination programs due to the diverse antigenicity.

Constant monitoring of the genomic characteristics of the circulating IBDV field isolates should be performed to cope with the high risk of vaccine-escaping IBDV variants’ emergence and spread. In this study, we selected two strains (rA and rB) that were isolated during the IBDV 2009 outbreak in California (USA) for whole genome sequencing (WGS), with the aim of providing more updated IBDV genetic information relevant to disease control strategies. Herein we define the probable virulence driving factors involved in virus reassortment, recombination, and positive selection, as well as the possible contribution of genes other than VP2 to virulence. Additionally, protein modeling of IBDV VP2 was conducted to define viral protein fitness and stability and the probable VP2 antigenic epitopes.

## 2. Methods

### 2.1. Virus Isolation and Propagation

The bursal tissues of IBDV strains rA and rB were obtained from the UC-Davis California Animal Health and Food Safety Laboratory (Beate Crossley) during a high mortality event associated with an IBD outbreak in two California layer flocks [27,28]. A 20% weight/volume suspension of bursal homogenate was prepared in phosphate-buffered saline (PBS) containing antibiotics (10,000 IU/mL Penicillin G, 1 mg/mL Gentamycin, 0.65 mg/mL Kanamycin sulfate, 2.0 mg/mL Streptomycin sulfate, and 20 µg/mL Amphotericin B). Bursal homogenate of both viruses in a 0.1 mL volume was injected separately into nine-day-old specific pathogen-free embryonated chicken eggs via the chorioallantoic membrane (CAM) route. Inoculated eggs were candled over a seven-day period, with mortality occurring in the first 24 h considered nonspecific, and eggs were discarded. After seven days, CAM, liver, and spleen were harvested from deceased embryos. Collected tissues were pooled, diluted to a 20% weight/volume suspension in PBS, homogenized, and freeze–thawed three times. Finally, the suspension was clarified by centrifugation at 12,000 rpm, and the supernatant was aliquoted into vials of 1 mL volume and frozen at −80 °C. until further use.

### 2.2. RNA Extraction and Reverse Transcription

RNA was extracted from 140 μL of CAM homogenate of each virus using the Qiagen viral mini-RNA isolation kit (Qiagen, USA) according to the manufacturer’s instructions. About 1 µg of total RNA was reverse-transcribed into cDNA in a 20 μL reaction volume containing 500 ng of oligo (dT), 1 µL of 10 mM dNTP mix, 5 μL of 5X buffer, 1 μL of 0.1 M DTT, and 1 μL of Superscript III reverse transcriptase (Invitrogen, USA). The reaction mixture was incubated at 50 °C for 60 min, followed by heat inactivation at 70 °C for 15 min.

### 2.3. IBDV Full-Length Genome Amplification and Cloning

For each virus, the whole genome of segment A was amplified by PCR using the forward primer (Seg A For, 5′-CGAGCTCGGTACCTAATACGACTCACTATAGGATACGATCGGTCTGACCCCGGGGG-3′) and the reverse primer (Seg A Rev, 5′-GGCTAAGATATCAGGGGACCCGCGAACGGGTCCAATTTGGATGTTGTATGGC-3′), yielding a product size of 3.5 kb. Similarly, the 2.8 kb segment B was PCR-amplified using the forward primer (Seg B For, 5′-GAGCTCGAATTCTAATACGACTCACTATAGGATACGATGGGTCTGACCCTCTGGG-3′) and the reverse primer (Seg B Rev, 5′-GGCTAACCGCGGGGGGGCCCCCGCAGGCGAA-3′). Briefly, a 25 μL PCR mixture was set up, containing 2 μL of cDNA template, 400-nM forward and reverse primer, 300 μM dNTPs, 5 μL of 5X LongAmp Taq Reaction Buffer, and 1 μL (2.5U) of LongAmp Taq DNA Polymerase (NEB, Ipswich, MA, USA). PCR was conducted at 94 °C for 3 min, followed by 35 cycles of 94 °C for 30 s, and 65 °C for 4 min 30 s, and a final extension at 65 °C for 10 min. A 1% agarose gel electrophoresis was used to visualize the 3.5 kb and 2.8 kb amplicons. Amplicons matching expected sizes were then purified from the electrophoresis gel utilizing the PureLink Quick Gel Extraction Kit (Invitrogen, Carlsbad, CA, USA) according to the manufacturer’s protocol. The purified products were ligated into the pGEMT easy vector (Promega, Madison, WI, USA) using the standard procedure for sequencing.

### 2.4. IBDV Whole Genome Sequencing and Raw Data Processing

Accurate quantification of purified plasmids containing either segment A or B derived from rA and rB isolates was conducted using the QubitTM dsDNA HS assay kit (Invitrogen, USA). Whole Plasmid Sequencing was performed using Oxford Nanopore long-read sequencing (Eurofins Genomics, Louisville, KY, USA) from 10 μL of purified plasmid at a concentration of 30 ng/μL. NanoPack was used for visualization and processing of long-read sequencing data [29]. To begin with, read filtering and trimming were conducted using NanoFilt, relying on read length, mean read quality, and mean GC content. The quality-filtered reads obtained were quality-checked using NanoStat and NanoPlot. NanoStat generated a comprehensive statistical data analysis, including mean and median read length, mean and median read quality, read length N50, number, percentage, and megabases of reads above quality cutoffs. Whereas the quality control graphs were obtained by NanoPlot, including read length histograms, cumulative yield plots, and bivariate plots (log transformed read lengths are compared with their mean quality score). The reads that passed the quality check were used for de novo assembly using Geneious Prime (Version 2023.0.1), and a consensus sequence was subsequently obtained.

### 2.5. Phylogenetic Analyses

The IBDV consensus sequences of rA and rB isolates were subjected to several phylogenetic analyses using the MEGA 11 software [30]. For rA and rB genotyping, the VP2 hypervariable regions (VP2-HVR, aa position 211–350) of both isolates were extracted manually in Geneious Prime together with the VP2-HVR sequences of 45 isolates representing the seven genotypes according to Jackwood’s classification [31]. The phylogenetic relatedness of the 47 sequences was estimated using the best fitting substitution model and the OH isolate (serotype 2, accession number: U30818) as an outgroup. For testing reassortment incidence, separate phylogenetic analyses of VP2 and VP1 genes were carried out according to the most recent dual-classification system [32] using MEGA 11 and compared with 176 and 174 sequences, respectively. The contribution of genes other than VP2 to IBDV virulence was tested via phylogenetic analysis to define any significant clustering with a well-defined, phenotype-based, very virulent clade [33,34]. The sequence alignments were generated using ClustalW with an opening penalty of 15 and an extension penalty of 6.66. Phylogenetic trees were constructed using the maximum likelihood method and the best-fit nucleotide substitution model using the minimum Bayesian information criterion. Moreover, the Akaike information criterion, maximum likelihood value (lnL), corrected value, and the count of parameters used (including branch lengths) were measured for each model. The reliability of the phylogenetic tree was estimated by the bootstrapping of 1000 replicates. The names and accession numbers of all IBDVs used in this study are summarized in Tables S3 and S8.

### 2.6. Detection of Recombination Incidence

Potential recombination events in IBDV genomic segments A and B were assessed using RDP4 version 4.101 [35]. RDP4 analysis was carried out based on the complete genomic sequence of each IBDV gene using the following seven different algorithms: RDP, BootScan, GENECONV, Chimera, SISCAN, maximum chi square, and 3SEQ. A putative recombination event was passed to consequent analysis only if it was plausibly defined by at least four of the above-mentioned seven algorithms. The minor parent was defined as the one contributing the smaller fraction of the obtained recombinant, whereas the major parent was the one contributing the larger fraction of the yielded recombinant [36]. Moreover, the recognized recombination events were detected with a Bonferroni-corrected *p*-value cut-off of 0.01.

### 2.7. Estimation of Selection Pressure

The selection pressures on the coding sequences of VP5, mature VP2, VP4, VP3, and VP1 proteins were estimated using Tajima’s D neutrality test and tested for statistical significance using the codon-based Z-test of selection in MEGA 11. The site-specific selection pressures were also detected by a non-synonymous (dN) to synonymous (dS) nucleotide substitution rate per codon using single likelihood ancestor counting (SLAC) and Fast, Unconstrained Bayesian AppRoximation for Inferring Selection (FUBAR).

### 2.8. Secondary Structure Investigation of IBDV VP2

The influence of the secondary structure of VP2 on virulence in the context of positive selection incidence was tested. Virulence-related aa hallmarks were assessed in terms of their positioning in the favored positions using SWISS-MODEL (https://swissmodel.expasy.org/ (accessed on 22 June 2023)) [37]. The detection and orientation of distinctive residues (prolines and cis prolines, rotamer outliers) and the abundance of possible residual outliers that impact protein stability and fitness were investigated using Ramachandran plotting and Molprobity [38]. The rA and rB VP2 sequences (target) were modeled against a crystalline structure of the VP2 (protein databank [PDB]:2df7) as a template. Furthermore, the models obtained for both rA and rB were used for alignment and comparison via Pymol (Schrodinger Ltd., New York, NY, USA).

## 3. Results

### 3.1. Genomic Organization of Isolates rA and rB

**Figure 1:**
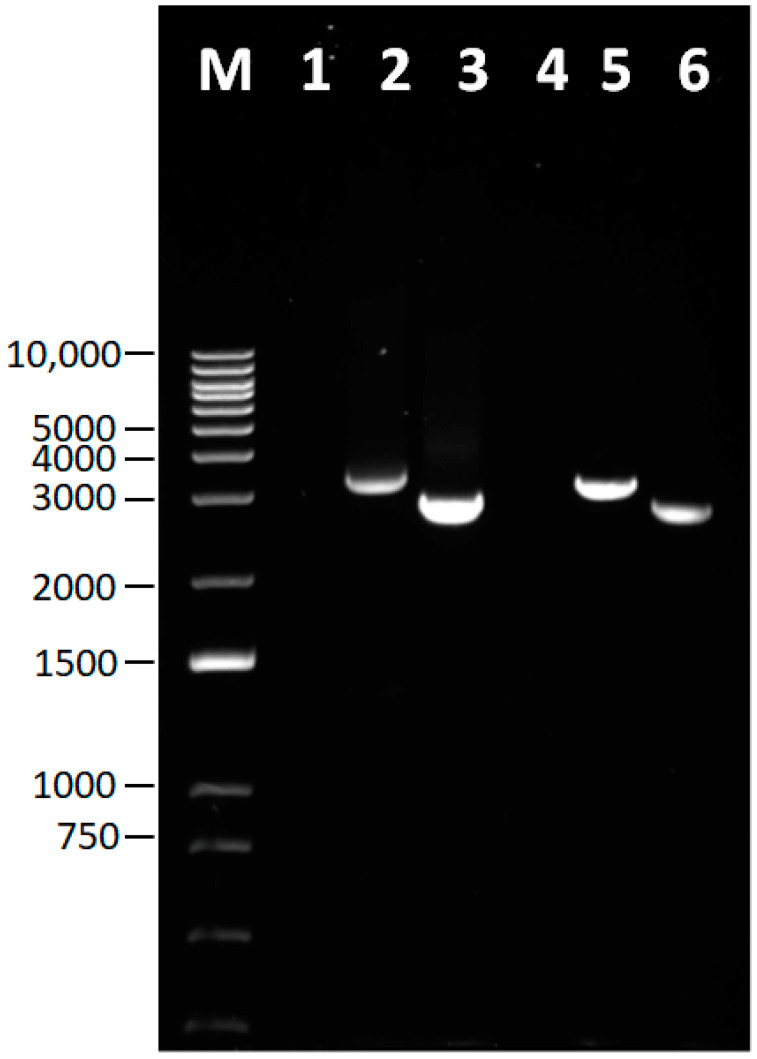
PCR amplification of segment A and segment B of IBDV strains rA and rB. Lane M—DNA ladder, number represents base pair; Lane 1—negative control for rA; Lane 2—rA segment A; Lane 3—rA segment B; Lane 4—negative control for rB; Lane 5—rB segment A; Lane 6—rB segment B.

The RT-PCR successfully amplified the whole genome of the two IBDV isolates (Figure 1). Moreover, the NGS-based sequence results of segment A and segment B for both rA and rB isolates contained 3261 bp and 2827 bp, respectively. The coding and non-coding regions are displayed in Table 1. The overall genomic organization of rA and rB genomic segments was identical. Segment A of both isolates contained the VP5 coding sequence ([CDS], 450 bp) overlapping the VP2-VP4-VP3 polyprotein CDS (3039 bp). In segment A, the coding region was flanked by 5′-UTR (84 bp) and 3′-UTR (92 bp), whereas segment B encoded the RNA-dependent RNA polymerase (VP1, 2640 bp), flanked by 5′-UTR (111 bp) and 3′-UTR (76 bp).

### 3.2. rA and rB Isolates Belong to the Genogroup 3 Based on HVR Genotyping

**Figure 2:**
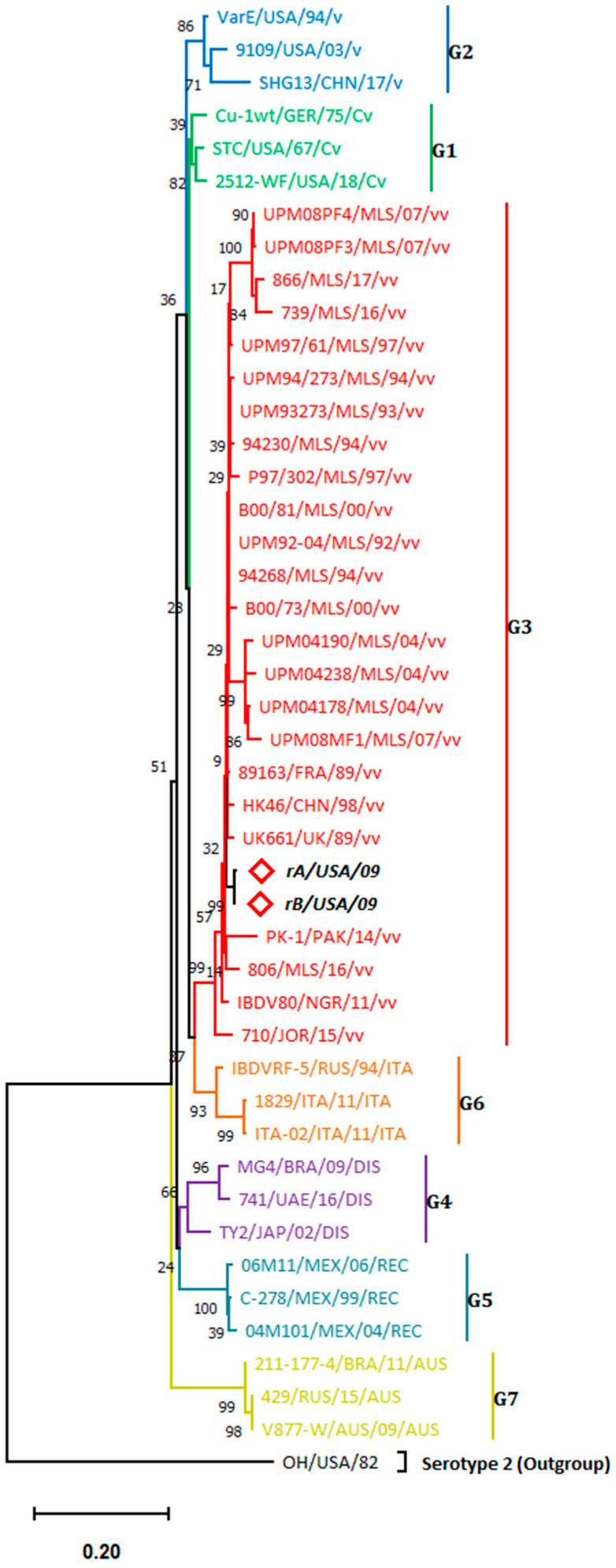
Phylogenetic tree for the IBDV-HVR-derived sequences constructed by the maximum likelihood method and Kimura 2-parameter model. The highest log likelihood tree is displayed (−3036.60). The percentage of associated taxa that are clustered together is provided at each branch. Heuristic search-dependent initial trees were produced automatically via Neighbor-Join and BioNJ algorithms applied to the pairwise distance matrix and assessed by the maximum composite likelihood (MCL) approach, followed by the highest log likelihood-resulted topology selection. Modeling the evolutionary rate differences among sites employed a discrete Gamma distribution (5 categories (+G, parameter = 0.6583)), according to the best fitting substitution model validation (Table S2). Accession numbers of sequences used for phylogenetic analysis are displayed in Table S3. Taxa nomenclature follows this order: Isolate_name/Country_of_origin/Collection_year/Classification.

Both sequenced IBDV isolates showed distinctive 761–1180 positioned nucleotides and 211–350 positioned amino acids according to the HVR numbering system of Bayliss et al. [6] The phylogenetic analysis of the VP2-HVRs of 47 IBDV isolates resulted in seven genogroups (Figure 2).

rA and rB isolates showed the highest nucleotide sequence homology to each other of 99.76% (i.e., showing the closest evolutionary relationship to each other, *d* = 0.002426, Table S1) and clustered together with other very virulent (vv) members of genogroup 3 (G3) with which they share 92.9–98.1% and 93.14–98.34% nucleotide homology, respectively. (Figure 2). Furthermore, rA and rB isolates were found to be highly evolutionary related to a vvIBDV isolate (89163) recovered earlier from France (*d* = 0.019589 and 0.017189, respectively). Since both isolates showed G3 clustering, the amino acid analysis was conducted to examine the amino acid hallmarks of the vvIBDVs.

### 3.3. HVR of rA and rB Contained the Virulence aa Hallmarks

**Figure 3:**
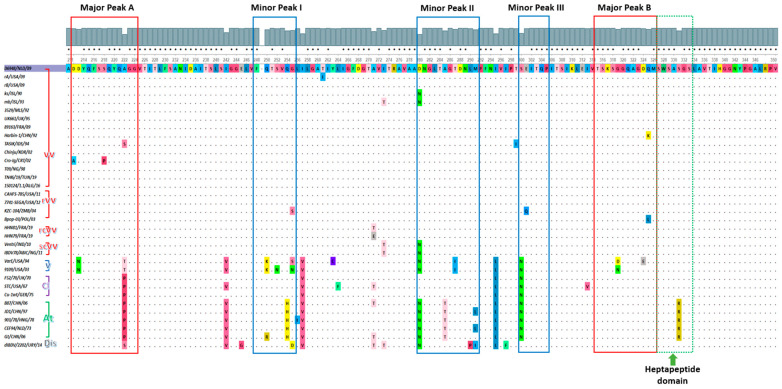
VP2 hypervariable region aa sequences of IBDV isolates (aa 211–350). Major hydrophilic peaks (A and B), minor hydrophilic peaks (I–III), and the heptapeptide domain are boxed with a red line, a blue line, and a dotted green line, respectively. Dots denote identical amino acids to the vv reference strain D6948 and were generated using UGENE (version 48). Accession numbers of sequences used for amino acid analysis are displayed in Table S4.

The deduced aa sequences of VP2-HVR further supported the phylogenetic relatedness of isolates rA and rB and the vv strains of genogroup 3 (Figure 3). Specifically, the IBDV isolate rB shared 100% aa identity with the vvIBDV isolates D6948 (reference strain), Gz, Hub-1, 89163, 3529, DD1, T09, 150124, 150144, TN46/19, and two reassortant vvIBDVs, including CAHFS-785 and 7741-SEGA. The closest aa identity of rA is with rB and all the sequences mentioned above, which each share an identical aa sequence, except for a single aa at position 260. This aa substitution T206I was distinctive to rA, and to our knowledge, it has not been reported previously. Interestingly, all amino acid hallmarks of vv strains existed in both rA and rB isolates, including 222A, 249Q, 286T, and 318G. Moreover, the reported amino acids characteristic of the vv Nigerian isolates [39] were entirely present in both isolates, including 242I, 256I, 294I, and 299S. Additionally, the HVR of rA and rB isolates preserves the serine-rich heptapeptide sequence SWSASGS (indicated by the doted green box in Figure 3—adjacent to the major peak B), which is a principal virulence marker.

### 3.4. rA and rB Do Not Show Evidence of Reassortment or Recombination

Since nucleotide and aa analysis assured the vv genotype based on the HVR genotyping, we investigated four molecular mechanisms that could contribute to the emergence of vv strains, including (i) reassortment, (ii) recombination, (iii) the contribution of other genes that may have accumulated significant mutations and have not been previously investigated, or (iv) positive selective pressure due to vaccination.

**Figure 4:**
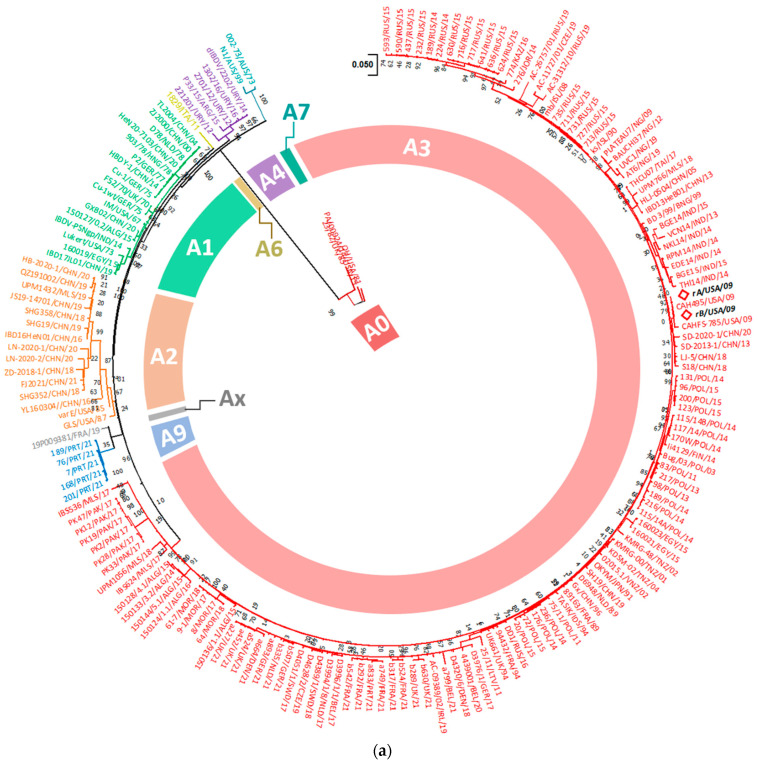

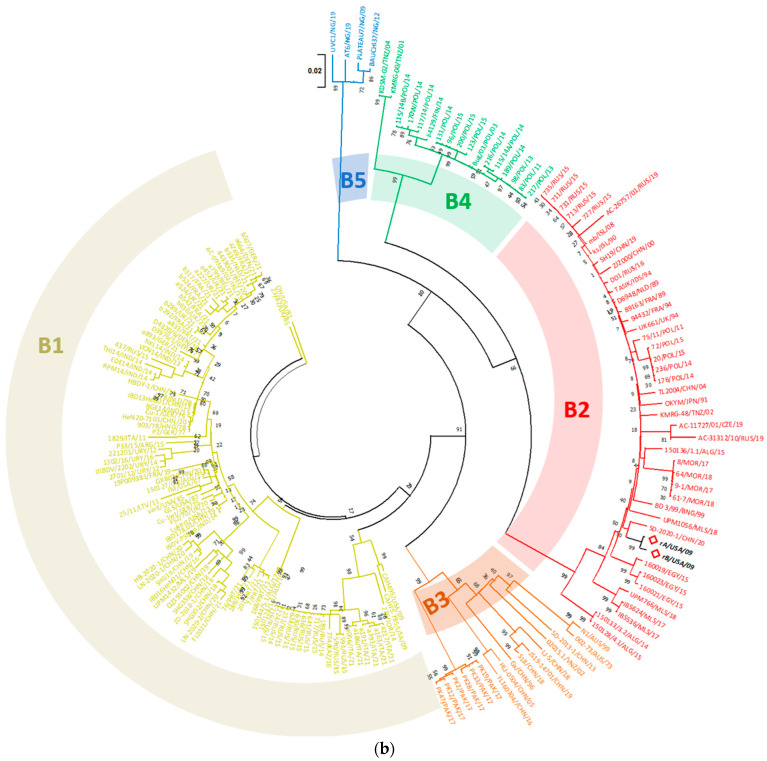
Phylogenetic analysis of the (**a**) truncated sequence of VP2 and (**b**) VP1 genes according to Wang et al. [32] rules with some modifications. Trees were inferred by using the Maximum Likelihood method and a General Time Reversible model constructed using a discrete Gamma distribution model evolutionary rate with differences among sites (five categories (+G), parameter = 0.3017 for VP2 and 0.2990 for VP1). The rate variation model allowed for some sites to be evolutionarily invariable ([+I], 30.91%, and 32.06% sites) for VP2 and VP1, respectively, according to the best fitting substitution model (Tables S6 and S7). The trees are drawn to scale, with branch lengths measured in the number of substitutions per site. Accession numbers of sequences used for phylogenetic analysis are displayed in Table S8. The main IBDV genogroups are denoted with designations and colors, and the taxa names of rA and rB strains are written in bold black with a red diamond. For enhanced visualization of our isolates’ orientation in VP2 and VP1 phylogenetic trees, non-closely related branches were collapsed/compressed and named after the country of origin (Figure S1).

We used a dual segment classification system to determine the capacity of rA and rB to exchange their genome segments during co-infection by reassortment. To begin with, phylogenetic analysis of VP2 showed that the rA and rB isolates were clustered within the A3 clade, which includes the very virulent strains (Figure 4a). Moreover, rA and rB were found to be closely related to the reassortant vv strain CAH495 (*d* = 0.004136 and 0.0033, Table S5) isolated from California in 2009, while VP1 analysis showed that rA and rB were not reassortants since both isolates were clustered within the B2 clade indicative of the vvIBDV strains (Figure 4b). Therefore, the current study shows that rA and rB strains are typical non-reassortant vvIBDVs belonging to the A3B2 genotype, unlike the reassortant vv Californian strain CAH495 that belongs to the genotype A3B1.

Next, the incidence of recombination in rA and rB isolates was determined. The RDP4 program was run to detect the putative recombinant events of IBDV genomic sequences for both segments A and B. No recombination events were detected by any of the seven algorithms for Californian isolates rA and rB when compared with IBDV sequences representative of all defined IBDV pathotypes. This finding supports these strains’ novelty owing to the absence of any probable breakpoints—indicative of recombination events.

### 3.5. Positive Contribution of IBDV Genes Leading to Virulence of rA and rB

A gene-wise phylogenetic analysis was conducted to investigate the clustering pattern of rA and rB with vvIBDVs, and any associated amino acid alterations that impacted the IBDV virulence of these strains were also identified.

#### 3.5.1. Gene-Wise Phylogenetic Analysis Showed Entire Clustering of rA and rB with vvIBDVs

**Figure 5:**
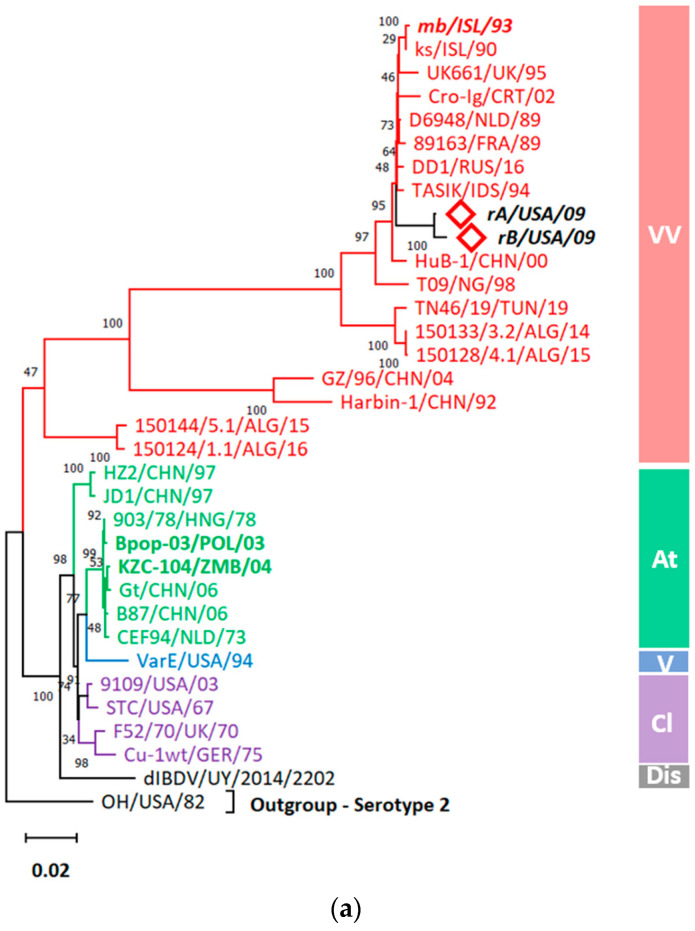

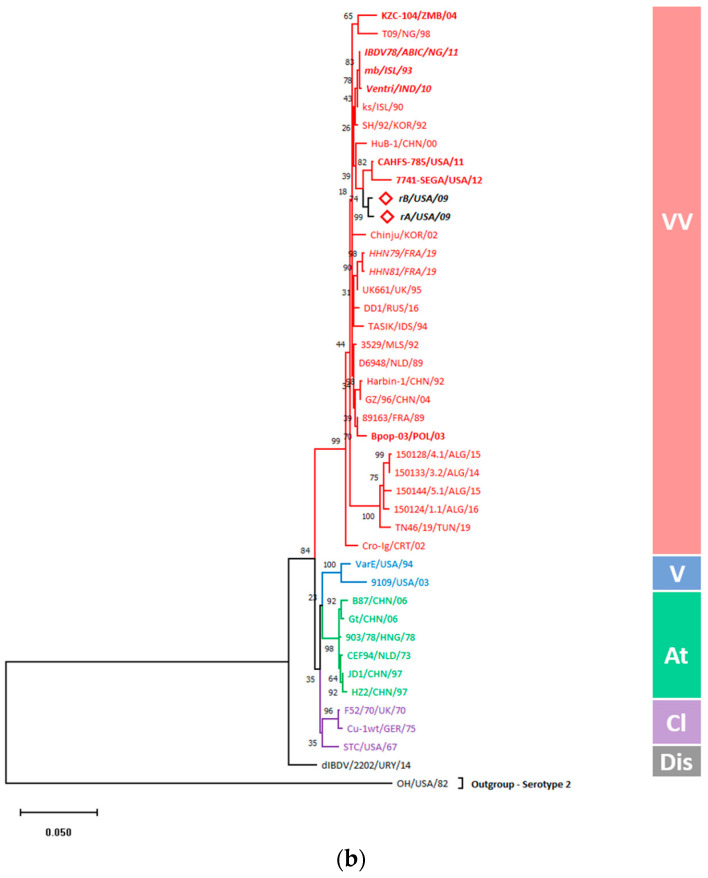

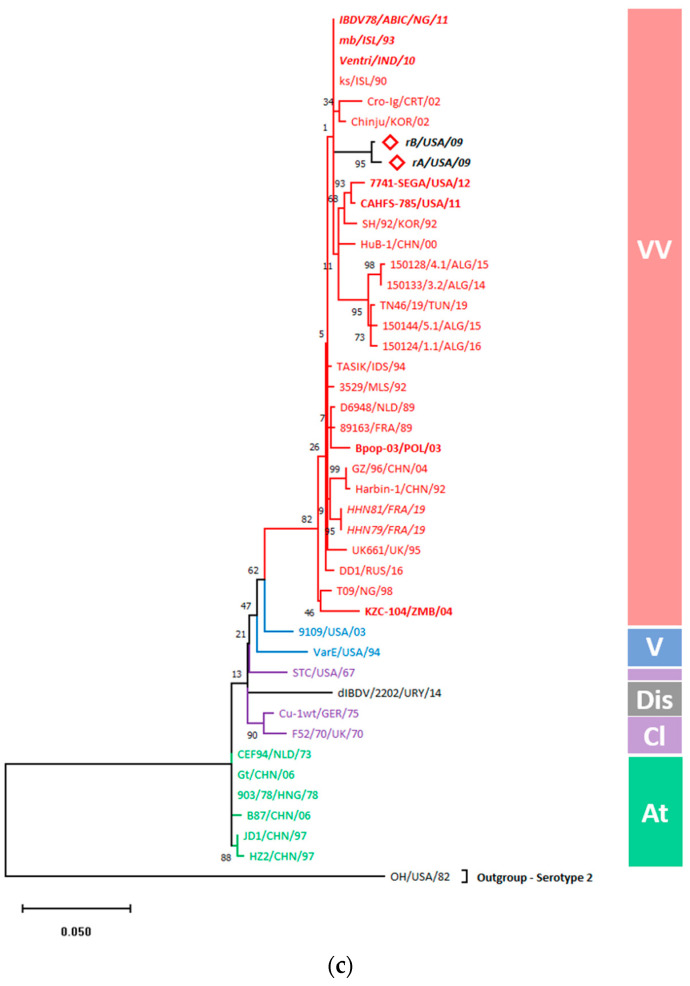

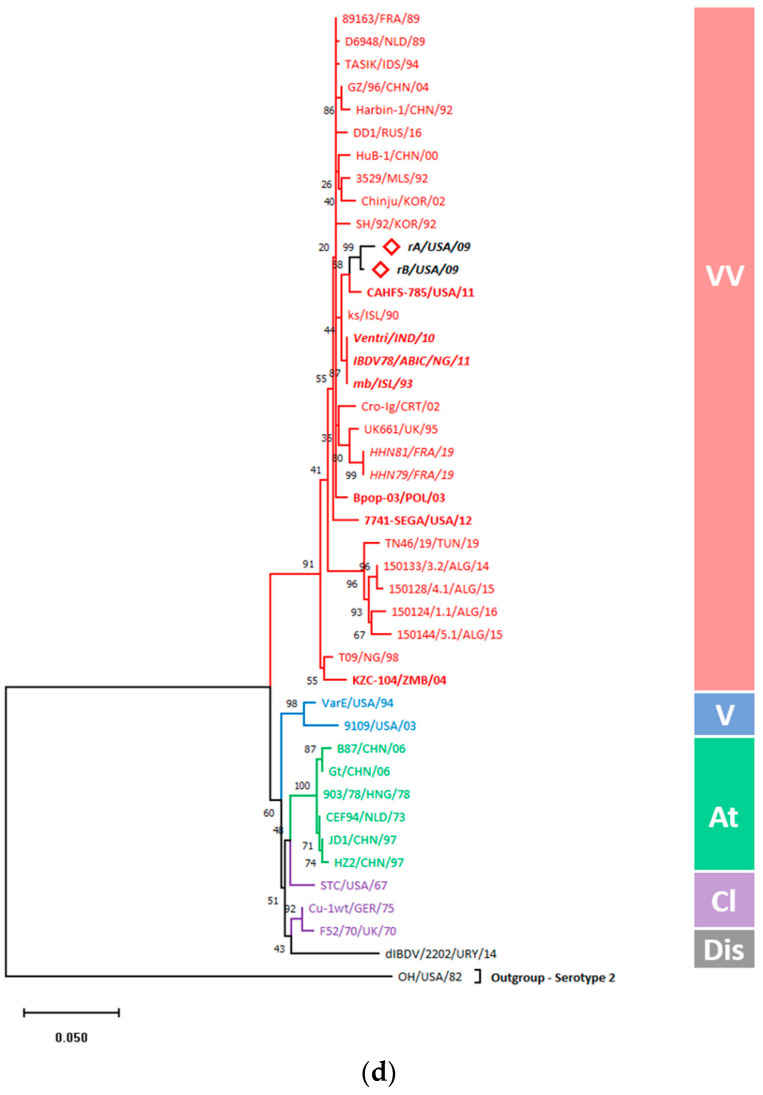

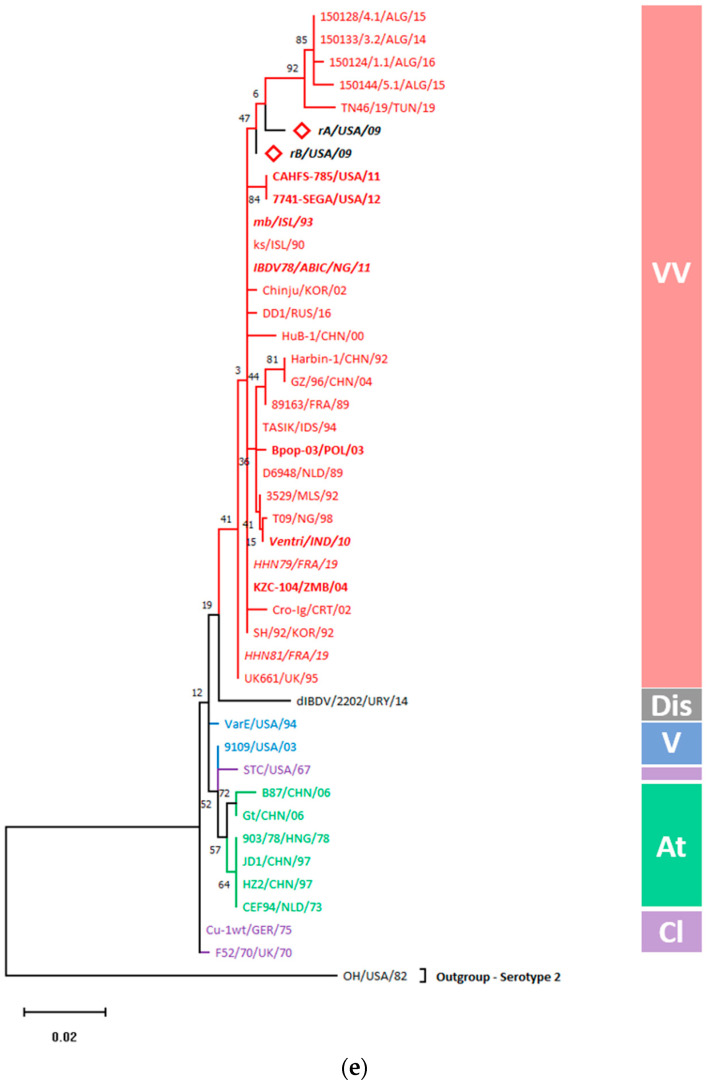
Gene-wise phylogenetic analysis. Evolutionary trees were inferred by using the Maximum Likelihood method and Tamura-Nei model using a discrete Gamma distribution model evolutionary rate differences among sites (5 categories, +G) for (**a**) VP1 (G = 0.2655) and (**c**) VP3 (G = 0.2298) or General Time Reversible model + G for (**b**) VP2 (G = 0.2060) or Kimura 2-parameter + G for (**d**) VP4 (G = 0.2433) and (**e**) VP5 (G = 0.1723) according to the most fitting nucleotide substitution model (Tables S14–S18). Accession numbers of sequences used for phylogenetic analysis are displayed in Table S4. The main IBDV phenotypes (according to virulence) are denoted with designations and colors, and the taxa names of rA and rB isolates are shown in bold black with red diamonds. Taxa shown in bold are reassortants, and red italicized bold taxa denote isolates that are serial passages from vv isolates.

Gene-wise phylogenetic analysis showed similar clustering of the entire genes of rA and rB with the vvIBDVs (Figure 5a–e). Interestingly, rA and rB isolates were found to be most closely related to each other in terms of all inspected genes (*d* = 0.00507 (Table S9), 0.005516 (Table S10), 0.005721 (Table S11), and 0.008475 (Table S12) for VP1 to VP4, respectively), except for VP5 (*d* = 0.006952, Table S13). Although VP5 phylogenetic analysis showed that rA was most related to rB, rB was the closest to the mb isolate (*d* = 0.002383, Table S13), which is an Israeli isolate that was serially passaged from the vv ks Israeli isolate—the next closest to the rB isolate (*d* = 0.002397). In the same manner, VP3 and VP4 of rA and rB were closely related to the vv ks VP3 (*d* = 0.022167 and 0.01915, respectively) and VP4 (*d* = 0.017208 and 0.011448, respectively). rA and rB VP1 was next evolutionary like the D6948 strain (vvIBDV, *d* = 0.01686 and 0.02137, respectively, Table S9) that was isolated from the Netherlands in 1989. On the contrary, rA and rB VP2 were next related to the Californian isolate CAHFS-785 (*d* = 0.01211 and 0.012896, respectively; Table S10) that was found to be a reassortant, vvIBDV.

#### 3.5.2. Amino Acid Substitution Analysis Denoted the Virulence Signatures within rA and rB Genes

The VP5 protein of rA, rB, and other viruses contains 149 amino acids that represent the first coding region of segment A. The N-terminal of the rA and rB VP5 proteins started with MLSL residues commonly found in most vvIBDV strains. rB showed the highest VP5 aa sequence identity homology (99.33%) to rA and the two Israeli isolates (ks and mb), whereas rA displayed a higher sequence homology to rB than to the Israeli isolates (98.66%).

The VP5 protein of rA and rB isolates contained the MLSL residues within the N-terminal as well as the amino acid substitution E18K. This aa substitution was common for all IBDV phenotypes (Table 2). Moreover, rA and rB shared the W80G that was also detected in the Tunisian (TN46/19) and Algerian isolates (150124/1.1, 150133/3.2, 150144/5.1, and 150128/4.1), whereas rA was found to have a unique mutation L95P. In addition, rA and rB VP5 contained R49, F78, P129, and W137 (Figure S2).

The second coding region in segment A is the PP that consists of the mature VP2 protein (441 amino acids) followed by the VP4 protein (243 amino acids), and eventually the VP3 protein (257 amino acids). To begin with, analysis of the VP2 protein of rA and rB isolates showed the highest amino acid sequence identity (99.09% and 99.31%, respectively) towards the very virulent isolates (D6948, DD1, 3529, UK661, 89163, HuB-1, GZ/96, Chinju, T09, TN46/19, 150124/1.1, and 150144/5.1) even more than towards each other (98.41%). Moreover, the rA and rB VP2 protein were found to have amino acid alterations at positions other than the HVR. rA isolates displayed three unique amino acid mutations in VP2, encompassing F59S, Y80H and G163E (Table 2). STC, a classical strain, encountered another mutation at a similar position (80Y) to rA with Y80L substitution (Figure S3). Furthermore, three other amino acid mutations including Y173C, V178A and F426S were found distinctive to the rB isolate.

The second protein of PP, VP4 (513–755 amino acids), showed the highest amino acid homology between rB and the ks isolate (99.58%), whereas rA VP4 accounted for the highest amino acid identity towards the rB isolate (98.76%) rather than the ks strain (98.35%). Analysis of the VP4 protein revealed three unique mutations for the rA isolate, including I535T, S604F, and V667A (Table 2). Moreover, we identified two other amino acid mutations shared between rA, rB and most vvIBDVs. Initially, the amino acid substitution K642N was detected in rA, rB, and vvIBDVs (ks, mb, 3529, CAHFS-785, Ventro, and IBDV78/ABIC). However, the classical strain F52/70 had the K130R mutation at the same position. The other amino acid substitution (N233S) was also detected in rA and rB, the same as in ks, mb, KZC-104, Ventri, and IBDV78/ABIC (vvIBDVs) strains. Significant amino acids, including 168Y, 173N, 203S, and 239D, were also detected in VP4 of both isolates (Figure S4).

The VP3 protein, the third protein of PP (amino acid position 756–1012), displayed the highest sequence identity of rA and rB to the Chinese isolate HuB-1 (99.61%). Moreover, VP3 amino acid analysis presented some extraordinary amino acid substitutions, two of which are distinctive to rA or rB isolates, and the other two were shared with selected vv IBDVs. To begin with, rA possessed the unique E18V mutation, unlike the M215V mutation detected in the rB isolate. Moreover, rA and rB shared the A19V substitution with only the HuB-1 strain. The last mutation, A235V, was a common mutation between rA and rB and most vv strains, as shown in Table 2.

Segment B of rA and rB contained 879 amino acids for VP1, which encodes the RNA-dependent RNA polymerase. rA and rB showed the highest amino acid homology of 98.97% to each other. Moreover, they showed higher sequence homology (99.88 and 99.86%, respectively) to the vv IBDV isolates including D6948, TASIK, DD1, and HuB-1 than variant (97.38–97.95% and 96.35–96.92%, respectively), classical (98.06 and 97.04%, respectively), and attenuated isolates (97.95–98.29% and 96.92–97.26%, respectively).

VP1 aa analysis of rA showed higher amino acid homology to the vv reference strain D6948 than rB with a single amino acid substitution (V4I) that was common with rB and all IBDV phenotypes involving the very virulent (TN46/19, 150133/3.2, and 150128/4.1), variant, classical, distinct, and attenuated isolates (Table 3). On the other hand, rB showed nine distinct amino acid alterations, including I111F, R186G, F378V, C424R, H494L, D513G, V545A, S607G, and L637P. These mutations have not been shared with any other strains except for the mutation at D513 that was found to be D513H in the Harbin-1 strain rather than D513G in the rB isolate. The VP1 protein signature sequence TDN, a characteristic for vvIBDVs, was also present in both isolates at aa 145–147 (Figure S6).

### 3.6. Selection Pressure Analysis Highlighted the Incidence of Both Purifying and Diversifying Selections

**Figure 6:**
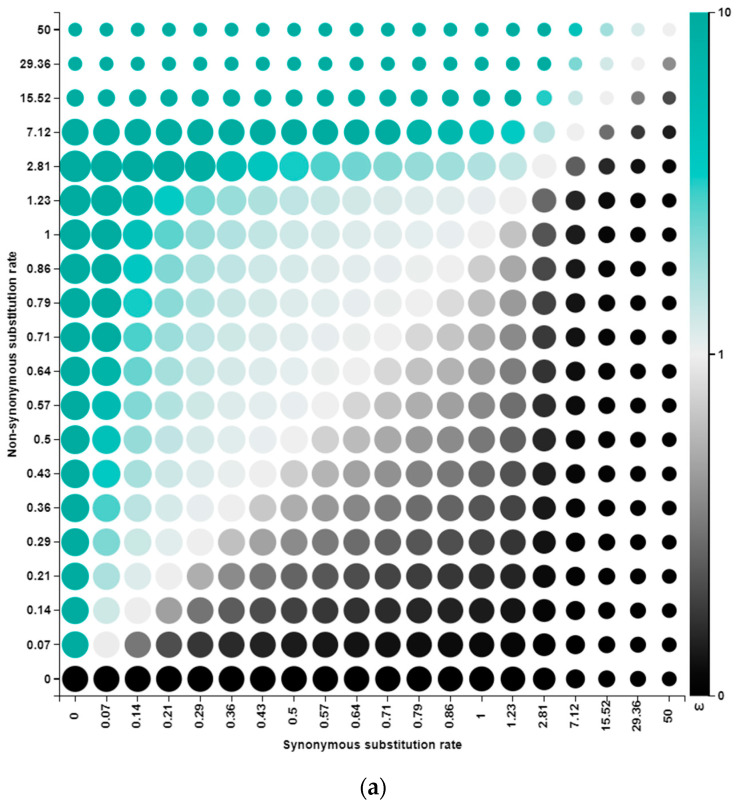

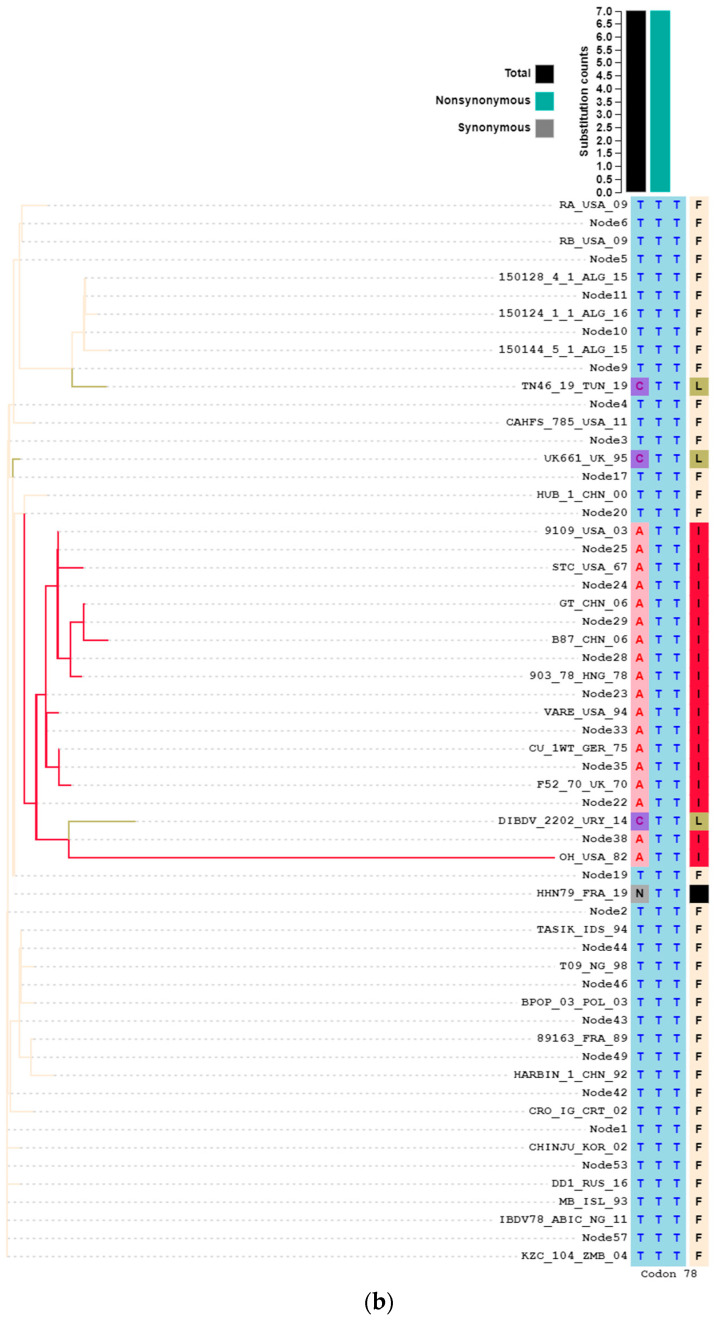
Selection pressure across VP5 sequences. (**a**) Alignment-wide posterior distribution graph over the discretized rate grid. The dot size is proportional to the posterior weight allocated to that grid point, and the color shows the intensity of selection (i.e., green refers to a possible positive selection, whereas black refers to a negative selection probability). (**b**) Codon-specific SLAC Phylogenetic Alignment Tree showing aa positive selection across different sequences representing all IBDV phenotypes. The tree branch color is equivalent to the amino acid at this site (codon 78).

The neutrality test for all segment A genes demonstrates a negative D value with no significance (*p* > 0.1) except for VP2, which was found to be statistically significant for codon-based positive selection at *p* = 0.00567 (Table 4). The SLAC and FUBAR analyses showed a significant deviation of most sites (*p* < 0.1) from neutrality to a negative selection. However, a single site (222) in mature VP2 or (13) in HVR, another site (168) in VP4, and five sites (44, 78, 104, 116, 138) in VP5 underwent positive selection. Roughly all the positive selection sites (6/7) were detected by the unconstrained FUBAR method (Table 4). Although the FUBAR method identified the entire positive selection in VP5 (Figure 6a), most of the positive selections in VP5 (4/5) were shared with all tested phenotypes. The positive selection at codon 78 of VP5 showed a differential pattern since 78F for almost all vv isolates, 78I for all classical, variant, and attenuated phenotypes, and 78L was only shared between the distinct strain and two vv IBDVs, involving UK661 and TN46/19 (Figure 6b). The SLAC method assured that positive selection at site 222 of PP, occurring in the major hydrophilic peak of HVR (codon 13), is of higher discrimination power genotypically than phenotypically (222A for most G3, 222S for only G4, 222Q for only G6, 222P for G1 and G7, and 222T is shared between G2 and G3, Figure S7). The distribution of codons under selection pressure displayed the highest negative selection in VP1 (50.6%), and the least negative selection was found in VP5 (1.34%). The greater substitution percentage across almost all genes was based on a random selection with no restraints (i.e., neutral selection).

Since the only statistically significant selection pressure was occurring in VP2 (222) and within the HVR (13), secondary structure analysis of mature VP2 was performed to define the positioning of this mutation and its impact on structure stability.

### 3.7. Secondary Structure Analysis Emphasized VP2 Protein Structure Fitness and Stability

**Figure 7:**
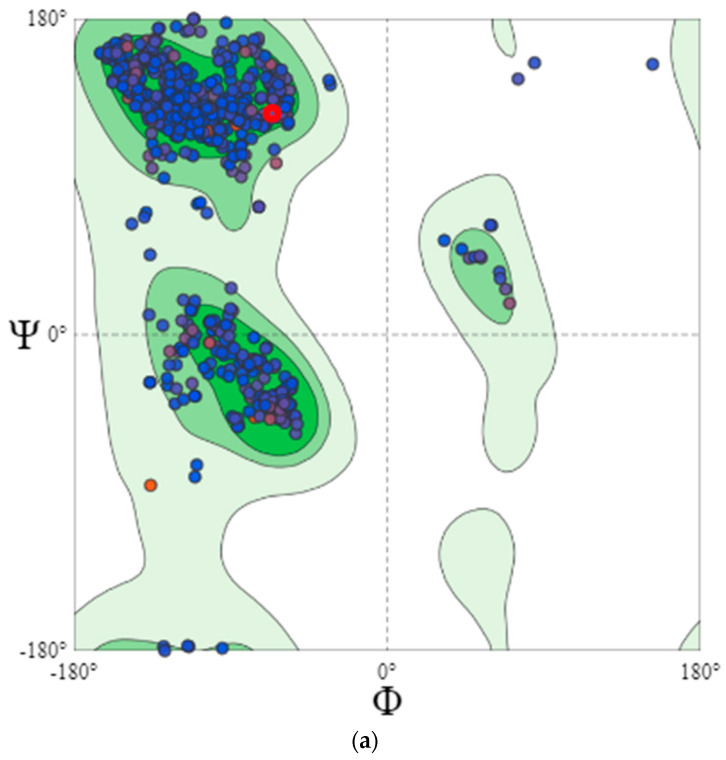

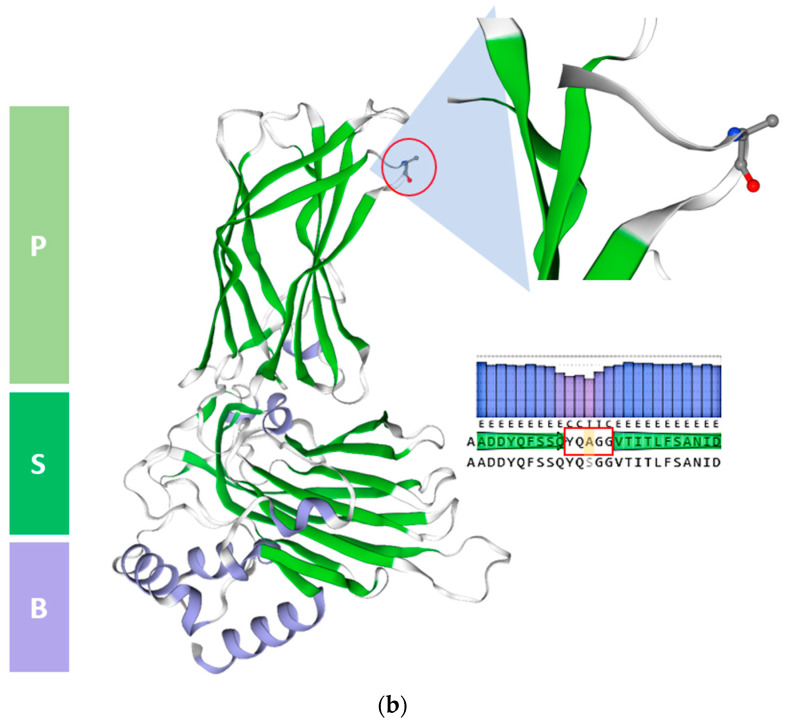
Secondary structure analysis of VP2. (**a**) Ramachandran plot showing VP2 structure fitness. The red circle indicates the aa under positive selection (222A) within the best-fitting position of the β-sheet. (**b**) VP2 3D model showing the positioning of 222A at the apical loop linking two β-strands.

The crystalline VP2 structure (PDB: 2df7) showed high fitness and similarity to the VP2 of rA and rB. Quality estimation of VP2 protein modeling showed the robustness of our VP2 model for downstream analysis (Figure S8). rA and rB secondary structure alignment was conducted using Pymol and showed no significant differences (Figure S9); consequently, rA was used for further analysis in terms of structure fitness and the impact of the positive selection pressure. The mature VP2 of rA and rB showed high structure fitness (Figure 7a), since Ramachandran favored positioning scored 96.14% with 2% rotamer outliers, including 330S, 315S, 219Q, 187I, 383L, 51D, and 121N. Therefore, the 222A that occurred within the positive selection site was present in the favorite positioning and does not impact the structure’s stability. However, how this mutation could have occurred or if it would impact viral attachment since VP2 is the virus attachment protein remains to be answered. The secondary structure analysis showed that this amino acid 222A exists within the YQ**A**GG motif at the apical loop linking two β-strands within the P-domain (Figure 7b). Then, solvent accessibility was checked by computing the solvent exposure using SPPIDER, which scored 52% for the relative solvent accessibility (RSA) of 222A (Figure 7). Moreover, the RSA of the whole motif containing the positive selection site (222A) ranged from 25% to 50%, with higher RSA for both amino acids flanking the 222A. Since RSA was above 20%, the whole motif is exposed with the highest exposure recorded by our positively selected codon, which justifies the reason for this amino acid substitution. The 222A was also found to be non-interface surface residues (RSA ≥ 25%), and consequently, it is not directly interacting with the ligand motif required for attachment.

## 4. Discussion

We performed deep molecular characterization of two IBDV isolates, rA and rB, which were reported earlier to be associated with a Californian outbreak with high mortality events (26% and 34%, respectively) with pathological manifestations demonstrated by inflammation in the bursa, spleen, thymus, severe lymphoid necrosis in Peyer patches, and cecal tonsils of layer pullets [28]. Therefore, these isolates were identified as vv IBDVs; however, the molecular characterization of the isolates was not sufficient to reveal the probable reasons beyond this vv phenotype since it relied on truncated sequence analysis, including HVR and partial VP1 sequences, as well as less representative sequences, not covering all possible IBDV phenotypes. Our analysis, however, was based on whole genome sequencing data, enabling the revelation of all possible attributes of virulence, including recombination, reassortment-based evolution, or even positive selection induced by the vaccination regime, as well as the possible contribution of other genes/regions within VP2 excluding the HVR.

To begin with, HVR analysis of rA showed the greatest sequence homology to rB, which was expected owing to its occurrence within the same outbreak in California. Both rA and rB were classified as belonging to IBDV genogroup G3, which includes other vv isolates also included in the previous study that earlier reported rA and rB as vv and closely related to UK661 [28]. However, we report that rA and rB isolates were found to be more closely related to the French vvIBDV (89163) recovered earlier in 1989 than the UK661. This discrepancy could be owing to the absence of this isolate in the phylogenetic analysis of the earlier study [28].

The rB HVR shared entire aa homology with most vv IBDVs, unlike the rA isolate, which encountered the unusual single aa substitution T260I located between the minor hydrophilic peaks I and II. Specifically, the mutation resides within a surface non-interacting residue of the E β-strand in the P-domain. Interestingly, rA and rB contained all five amino acid hallmarks of virulent strains, including 222A, 249Q, 284A, 286T, and 318G [9,15], further supporting the classification of these isolates as members of genogroup 3. Of particular interest is the aa 222 because of its apical positioning at the BC loop of the P domain, whose changes have been reported to impact vaccine efficacy [40]. In addition, 249Q seems to be an essential aa since it resides within the P_DE_ loop (aa 247–254), and any changes in this loop were found to be associated with antigenic drift and impaired virulence [11,41]. Moreover, 253Q and 284A, detected in our isolates, were found to be related to cell tropism and viral virulence in another recent study [42]. Consequently, aa substitution at position 284 could impact the strain’s virulence. In fact, A284T substitution was reported to assist in cell culture adaptation of a vv IBDV strain [43,44,45]. Furthermore, the aa at position 318 within the P_HI_ loop is essential in growth kinetics since 318 aa mutants depict lower viral titers, besides influencing the formation of antigenic determinants [9,17]. Furthermore, the four aa signatures of Nigerian vv IBDVs (242I, 256I, 294I, and 299S) [39] that are considered virulence markers with a significant share in cellular tropism, and pathogenicity outcomes were present in both rA and rB strains [46]. For instance, the aa substitution V256I was found to surge IBDV virulence and, being very close to the minor P_DE_ hydrophilic peak A, is suspected to be associated with potent antigenic activity [47]. In addition, the 299S, which resides within a groove, was reported to play a principal role in IBDV binding to susceptible cells [19]. The serine-rich heptapeptide sequence (SWSASGS), found in the rA and rB HVR sequences adjacent to the major hydrophilic peak B (aa 326–332), was reported as a signature for vvIBDV in other isolates [26,48]. The inter- and intra-molecular interactions are enhanced by the hydrogen bonding at the serine-rich motif required for typical viral virulence [34]. Indeed, single or double serine substitutions would result in a larger space in terms of the molecular configuration of the yielded protein, which hampers such interactions at the molecular level in low- or avirulent viruses [49].

While the presence of these potential aa signatures in the HVR region defines rA and rB virulence, other factors may also have a significant impact on the IBDV pathotype. Double-stranded viruses, including IBDV, are frequently depicted with high rates of point mutations, reassortment, and recombination [50]. For instance, novel IBDV reassortants involving A3B1, A3B3, and A3B5 genotypes were found to be very virulent and circulating across western Europe [51,52], China [53], and Nigeria [39], respectively. The HVR of the rB isolate shared entire amino acid sequence homology with two reassortant vv strains (CAHFS-785 and 7741-SEGA, collected from the USA in successive years). In addition, mature VP2 proteins of both rA and rB isolates displayed evolutionarily close relationships to the Californian isolate (CAH495, isolated in 2009). The current study isolates were found, however, to belong to the non-reassortant vvIBDVs with the A3B2 genotype, which has been globally predominant since its initial outbreak in Belgium in 1987 [53,54]. Our findings negate the possibility of reassortment as the driving force behind the rA and rB vv phenotypes. It also assures that HVR is not quite sufficient for defining the IBDV genotype and associated virulence and that inclusion of VP1 is essential; VP1 was also reported to modulate IBDV virulence [55].

Recombination within virus segments may also lead to the evolution of novel IBDV strains with molecular adaptation and enhanced virulence, particularly in the presence of vaccine strains [53]. A homologous recombination was reported between the intermediate vaccine strain and the novel IBDV variant (nVarIBDV), in which thirty regions of the vIBDV segment A were replaced by the equivalent regions of the vaccine strain, displaying higher pathogenicity in chick embryos [56]. rA and rB showed no recombination events when compared with IBDV sequences representative of all defined IBDV pathotypes, as indicated by the entire absence of any recombination breakdown positions, assuring the novelty of these strains at the time of collection.

IBDV virulence has been reported to be related to several genes, not exclusively to VP2 [34]. VP5, for example, drives virus dissemination from infected cells and contributes to pathogenesis via the induction of late apoptosis [57,58]. Consequently, gene-wise analysis was conducted, and all genes confirmed entire clustering within the vvIBDVs clade. The N-terminal of VP5 in rA and rB included MLSL residues that were commonly found in vvIBDVs [5]. Additionally, rA and rB VP5 contained R49, F78, P129, and W137, characteristic of vvIBDVs [59]. Although E18 was defined as distinctive to vv isolates, we encountered E18K substitution in both isolates, which was also common in various IBDV vv phenotypes [59]. Therefore, both strains preserved their VP5-related vv phenotype. The VP5 gene deletion displayed a significant reduction in bursal lesions compared to wild-type IBDVs [60]. The VP2, encoding the major capsid protein, remains the crucial gene involved in pathogenicity, virulence, and tropism [61], and consequently, it defines the virus’s genetic and antigenic patterns [8,62]. Aside from the earlier HVR discussion, VP2 showed other amino acid substitutions that were distinctive to each isolate. rA and rB showed three mutations each, but those are not exposed aa residues (RSA < 20%). However, a single mutation (G163E) in rA and two mutations (Y173C and V178A) in rB were significant mutations since they reside within the predicted interacting motifs (E**E**V, **C**DL, and Y**A**RL) within the loop and initiate the β-strand at the valine residue, completely within the loop or within the β-strand, respectively. Furthermore, variations within the VP4 protein sequences were suggested to influence IBDV virulence [63], since it encodes the viral protease required for the PP intermediate precursor cleavage [64]. Previous studies showed that 680 Y, 685 N, and 715 S and 751 D are characteristic for the vvIBDV PP and reside in VP4 [65,66]. Likewise, all these significant amino acids were detected in our isolates equivalent to 168Y, 173N, 203S, and 239D in VP4, confirming the vv phenotype of rA and rB. In VP3, we encountered a significant substitution (A235V) that is equivalent to position 990 in the PP and distinctive to vvIBDV strains, in line with previous findings [65,67]. The 990A was detected in avirulent IBDV strains and was associated with reduced viral replication [68]. Moreover, any aa alteration in definite VP3 positions, including 783, 918, 981, 990, and 1005, following SPF embryos- or fibroblast cultures-mediated attenuation process, could result in reduced virulence [69]. Interestingly, these sites preserved its amino acids, favoring the vv phenotype in rA and rB isolates. On the other hand, segment B encodes the VP1 protein that significantly contributes to IBDV pathogenicity and virulence [55,70]. We detected amino acid substitution (V4I) within the VP1 N-terminus region that is responsible for protein priming of IBDV. This mutation was reported previously in the HN strain and was suggested for the hypervirulence of the vvIBDV strains [71]. Moreover, the TDN aa triplet demonstrated in rA and rB is considered a virulent marker, influencing the IBDV RNA-dependent RNA polymerase (RdRP) [72,73]. The rB isolate encountered eight aa substitutions within the central domain of RdRP (aa 168–658), required for nucleotide recognition and binding [71]. Nevertheless, the significant aa residues defining the vv strains, including 287A, 508K, and 511S, remained unchanged [24]. To sum up, all genes showed a potential share in the high virulence of our isolates with non-destructive mutations.

Moreover, the mutant spectra in the IBDV composition within infected chicks during the Californian outbreak could also be justified by the quasispecies nature of IBDV [74,75]. The circulating vaccine strains induce multiple selection pressures that result in virus evolution associated with the emergence of various genotypes [19,76].

Notoriously, half and about a third of the whole codons of VP1 and PP, respectively, were found evolving under purifying selection. This phenomenon denotes that both proteins underwent restrictive evolution (i.e., were less prone to high sequence variations). We encountered multiple positive selections, some of which were within virulence determinant sites. They were, however, not linked to virion-dependent intramolecular interactions [77]. Also, the diversifying selection was entirely detected within the PP. The only significant positive selection was at site 222, which resides in the P_BC_ loop (in the major hydrophilic peak A), reported to be under constant host immunity-mediated selection pressure [78]. Moreover, our VP2 protein modeling showed this aa residue to be exposed and consequently prone to frequent selections. Therefore, this site denotes a typical antigenic variation region for IBDVs [9]. More positive selection pressures were detected in VP5, though they were not related to virulence determinants. A mutant virus with VP5 deletion displayed diminished virulence, which still highlights the possible impact of deleterious changes in VP5 on IBDV virulence or antigenicity [60]. A previous study reported positive selection at the dsRNA binding domain (site 990), but that was not the case in our study. Although we encountered this mutation (A990V), it was found to be due to a random mutation rather than positive selection. Furthermore, we detected both aa residues (A and V) in other vv isolates similar to those reported elsewhere [79].

In conclusion, the current study revealed the driving molecular factors behind the outbreak incidence triggered by these vvIBDV isolates, rA and rB. Reassortment and recombination were not the virulence inducers, and consequently, no parental strains to share their segments or partial sequences were found, confirming the novelty of these strains. On the other hand, each gene showed characteristic virulence signatures, while novel mutations were detected in rA and rB, some of them in significant interacting sites. The significant positive selection in VP2 was found to not negatively impact the viral attachment protein fitness and stability and could enhance viral virulence since it was recognized within the favorite protein orientation. Furthermore, other positive selections were observed that would require investigation via reverse genetics to define their corresponding roles in IBDV virulence.

## Figures and Tables

**Table 1:** Whole genome annotation of both IBDV isolates.

**Segment A**							
5′UTR	VP5	VP2			VP4	VP3	3′UTR
		*Whole*	*Mature*	HVR *			
1–84	85–534	131–1666	131–1453	761–1180	1667–2395	2396–3169	3170–3261
**Segment B**							
5′UTR		VP1			3′UTR		
1–111		112–2751			2752–2827		

* HVR: highly variable region of mature VP2. rA segments A and B were deposited in Genbank with accession numbers OR528907 and OR528909, respectively. Segments A and B of the rB isolate were denoted with accession numbers OR528908 and OR528910, respectively.

**Table 2:** Amino acid substitutions in the segment A genes of study isolates.

Strain	Phenotype	VP5			Mature VP2 (Excluding HVR)						VP4					VP3			
		18	80	95	59	80	163	173	178	426	535 (23) ^¥^	604 (92)	642 (130)	667 (155)	745 (233)	773 (18)	774 (19)	970 (215)	990 (235)
**D6948**	Very virulent	**E**	**W**	**L**	**F**	**Y**	**G**	**Y**	**V**	**F**	**I**	**S**	**K**	**V**	**N**	**E**	**A**	**M**	**A**
rA		K	G	P	S	H	E	*	*	*	T	F	N	A	S	V	V	*	V
rB		K	G	*	*	*	*	C	A	S	*	*	N	*	S	*	V	V	V
SH		K	*	*	*	*	*	*	*	*	*	*	*	*	*	*	*	*	V
ks		K	*	*	*	*	*	*	*	*	*	*	N	*	S	*	*	*	V
mb		K	*	*	*	*	*	*	*	*	*	*	N	*	S	*	*	*	V
DD1		K	*	*	*	*	*	*	*	*	*	*	*	*	*	*	*	*	V
3529		*	*	*	*	*	*	*	*	*	*	*	N	*	*	*	*	*	V
UK661		K	*	*	*	*	*	*	*	*	*	*	*	*	*	*	*	*	V
89163		*	*	*	*	*	*	*	*	*	*	*	*	*	*	*	*	*	*
HuB-1		K	*	*	*	*	*	*	*	*	*	*	*	*	*	*	V	*	V
GZ/96		*	*	*	*	*	*	*	*	*	*	*	*	*	*	*	*	*	V
Hairbin-1		*	*	*	*	*	*	*	*	*	*	*	*	*	*	*	*	*	V
TASIK		*	*	*	*	*	*	*	*	*	*	*	*	*	*	*	*	*	V
Chinju		K	*	*	*	*	*	*	*	*	*	*	*	*	*	*	*	*	V
Cro-Ig		K	*	*	*	*	*	*	*	*	*	*	*	*	*	*	*	*	*
T09		*	*	*	*	*	*	*	*	*	*	*	*	*	*	*	*	*	V
TN46/19		K	G	*	*	*	*	*	*	*	*	*	*	*	*	*	*	*	*
150124/1.1		K	G	*	*	*	*	*	*	*	*	*	*	*	*	*	*	*	*
150133/3.2		K	G	*	*	*	*	*	*	*	*	*	*	*	*	*	*	*	*
150144/5.1		K	G	*	*	*	*	*	*	*	*	*	*	*	*	*	*	*	*
150128/4.1		K	G	*	*	*	*	*	*	*	*	*	*	*	*	*	*	*	*
CAFHS-785		K	*	*	*	*	*	*	*	*	*	*	N	*	*	*	*	*	V
7741-SEGA		K	*	*	*	*	*	*	*	*	*	*	*	*	*	*	*	*	V
KZC-104		K	*	*	*	*	*	*	*	*	*	*	*	*	S	*	*	*	V
Bpop-03		*	*	*	*	*	*	*	*	*	*	*	*	*	*	*	*	*	*
HHN81		*	*	*	*	*	*	*	*	*	*	*	*	*	*	*	*	*	*
HHN79		*	*	*	*	*	*	*	*	*	*	*	*	*	*	*	*	*	*
Ventri		*	*	*	*	*	*	*	*	*	*	*	N	*	S	*	*	*	V
IBDV78/ABIC		K	*	*	*	*	*	*	*	*	*	*	*	*	S	*	*	*	V
VarE	Variant	K	*	*	*	*	*	*	*	*	*	*	*	*	*	*	*	*	*
9109		K	*	*	*	*	*	*	*	*	*	*	*	*	*	*	*	*	*
F52/70	Classical	K	*	*	*	*	*	*	*	*	*	*	R	*	*	*	*	*	*
STC		K	*	*	*	L	*	*	*	*	*	*	*	*	*	*	*	*	*
Cu-1wt		K	*	*	*	*	*	*	*	*	*	*	*	*	*	*	*	*	*
B87	Attenuated	K	*	*	*	*	*	*	*	*	*	*	*	*	*	*	*	*	*
JD1		K	*	*	*	*	*	*	*	*	*	*	*	*	*	*	*	*	*
HZ2		K	*	*	*	*	*	*	*	*	*	*	*	*	*	*	*	*	*
903/78		K	*	*	*	*	*	*	*	*	*	*	*	*	*	*	*	*	*
CEF94		K	*	*	*	*	*	*	*	*	*	*	*	*	*	*	*	*	*
Gt		K	*	*	*	*	*	*	*	*	*	*	*	*	*	*	*	*	*
dIBDV	Distinct	K	*	*	*	*	*	*	*	*	*	*	*	*	*	*	*	*	*

^¥^: The numbers between brackets refer to the aa position within a particular gene (Figures S4 and S5), whereas the number outside the bracket refers to the aa position within the VP2-VP4-VP3 polyprotein (PP). The highlighted asterisks refer to amino acids identical to the reference sequence displayed in bold.

**Table 3:** The characteristic amino acids in the VP1 gene of study isolates.

Strain	Phenotype	VP1									
		4	111	186	378	424	494	513	545	607	637
**D6948**	Very virulent	**V**	**I**	**R**	**F**	**C**	**H**	**D**	**V**	**S**	**L**
rA		I	*	*	*	*	*	*	*	*	*
rB		I	F	G	V	R	L	G	A	G	P
ks		*	*	*	*	*	*	*	*	*	*
mb		*	*	*	*	*	*	*	*	*	*
DD1		*	*	*	*	*s	*	*	*	*	*
UK661		*	*	*	*	*	*	*	*	*	*
89163		*	*	*	*	*	*	*	*	*	*
HuB-1		*	*	*	*	*	*	*	*	*	*
GZ/96		*	*	*	*	*	*	*	*	*	*
Hairbin-1		*	*	*	*	*	*	H	*	*	*
TASIK		*	*	*	*	*	*	*	*	*	*
Cro-Ig		*	*	*	*	*	*	*	*	*	*
T09		*	*	*	*	*	*	*	*	*	*
TN46/19		I	*	*	*	*	*	*	*	*	*
150124/1.1		*	*	*	*	*	*	*	*	*	*
150133/3.2		I	*	*	*	*	*	*	*	*	*
150144/5.1		*	*	*	*	*	*	*	*	*	*
150128/4.1		I	*	*	*	*	*	*	*	*	*
VarE	Variant	I	*	*	*	*	*	*	*	*	*
9109		I	*	*	*	*	*	*	*	*	*
F52/70	Classical	I	*	*	*	*	*	*	*	*	*
JD1	Attenuated	I	*	*	*	*	*	*	*	*	*
HZ2		I	*	*	*	*	*	*	*	*	*
CEF94		I	*	*	*	*	*	*	*	*	*
dIBDV	Distinct	I	*	*	*	*	*	*	*	*	*

The highlighted asterisks refer to amino acids identical to the reference sequence displayed in bold.

**Table 4:** Codon-based selection pressures on the current study isolates.

Protein		Tajima’s D	Z-Test	dN/dS	Positively Selected Sites		Negatively Selected Sites	
					SLAC (Codon)	FUBAR (Codon)	SLAC	FUBAR
VP1		0.72408	NS	0.0473	0	0	133	445
VP5		−1.35217	NS	1.62	0	5 (44, 78, 104, 116, 138)	2	2
PP	VP2	−1.02634	**SS**	0.111	0	1 (222)	66	171
	VP4	−0.85195	NS	0.0938	0	1 (168 [680 *])	26	65
	VP3	−1.35183	NS	0.0983	0	0	37	74
HVR		−0.47423	NS	0.161 ^¥^/0.162 ^§^	0 ^¥^/1 ^§^ (13[222] *)	1 (13[222] *)	16 ^¥^/49 ^§^	30 ^¥^/65 ^§^

NS: non-significant (*p* > 0.05), SS: statistically significant (*p* = 0.00567, i.e., *p* < 0.05). *: codon position in the polyprotein (PP). ^¥^: Phenotype-dependent, ^§^: Genotype-dependent.

## Data Availability

rA and rB whole genome sequences were deposited in GenBank with accession numbers OR528907–OR528910. Moreover, the sequences used in this study for phylogenetic analysis are openly available in the NCBI GenBank repository, with accession numbers mentioned in Tables S3, S4 and S8.

## Supplementary Materials

The following supporting information can be downloaded at: https://www.mdpi.com/article/10.3390/v15102044/s1, Figure S1. Compressed phylogenetic trees of the (a) truncated sequence of VP2 and (b) VP1 genes. Figure S2. VP5 amino acid sequences of IBDV isolates (aa 1–149). Figure S3. VP2 amino acid sequences of IBDV isolates (aa 1–441). Figure S4. VP4 amino acid sequences of IBDV isolates (aa 1–243). Figure S5. VP3 amino acid sequences of IBDV isolates (aa 1–257). Figure S6. Aligned VP1 amino acid sequences of IBDV isolates (aa 1–879). Figure S7. Codon-specific SLAC Phylogenetic Alignment Tree showing aa positive selection across different sequences representing all IBDV phenotypes. Figure S8. Quality estimation of VP2 protein modeling via (a) local quality estimate and (b) comparison with a non-redundant set of PDB structures. Figure S9. VP2 3D model alignment of both rA and rB isolates using Pymol. Table S1. Evolutionary divergence estimates between HVR sequences of IBDV isolates. Table S2. Best fitting model selection for VP2 HVR using Maximum Likelihood fits of 24 different nucleotide substitution models. Table S3. Accession numbers of sequences used for phylogenetic analysis of IBDV HVR for genotyping. Table S4. Accession numbers of sequences used for amino acid sequence analysis of VP2-HVR. Table S5. Evolutionary divergence estimates between mature VP2 sequences of the study isolates and other IBDV isolates. Table S6. Best fitting model selection for truncated VP2 using Maximum Likelihood fits of 24 different nucleotide substitution models. Table S7. Best fitting model selection for VP1 using Maximum Likelihood fits of 24 different nucleotide substitution models. Table S8. Accession numbers of sequences used for reassortment analysis of Californian isolates. Table S9. Evolutionary divergence estimates between mature VP1 sequences of the study isolates and other IBDV isolates for gene-wise analysis. Table S10. Evolutionary divergence estimates between VP2 sequences of the study isolates and other IBDV isolates for gene-wise analysis. Table S11. Evolutionary divergence estimates between VP3 sequences of the study isolates and other IBDV isolates for gene-wise analysis. Table S12. Evolutionary divergence estimates between VP4 sequences of the study isolates and other IBDV isolates for gene-wise analysis. Table S13. Evolutionary divergence estimates between VP5 sequences of the study isolates and other IBDV isolates for gene-wise analysis. Table S14. Best fitting model selection for VP1 using Maximum Likelihood fits of 24 different nucleotide substitution models. Table S15. Best fitting model selection for mature VP2 using Maximum Likelihood fits of 24 different nucleotide substitution models. Table S16. Best fitting model selection for mature VP3 using Maximum Likelihood fits of 24 different nucleotide substitution models. Table S17. Best fitting model selection for mature VP4 using Maximum Likelihood fits of 24 different nucleotide substitution models. Table S18. Best fitting model selection for mature VP5 using Maximum Likelihood fits of 24 different nucleotide substitution models.
