# Supplementary material for: Whole Genome Sequencing of Infectious Bursal Disease Viruses Isolated from a Californian Outbreak Unravels the Underlying Virulence Markers and Highlights Positive Selection Incidence
Source: Viruses. 2023 Oct 3;15(10):2044. doi: 10.3390/v15102044 (PMC10612053; doi:10.3390/v15102044)
Supplement: Supplementary file 1 [file viruses-15-02044-s001.zip › viruses-2635247-supplementary.pdf]

Supplementary information

A. Figures

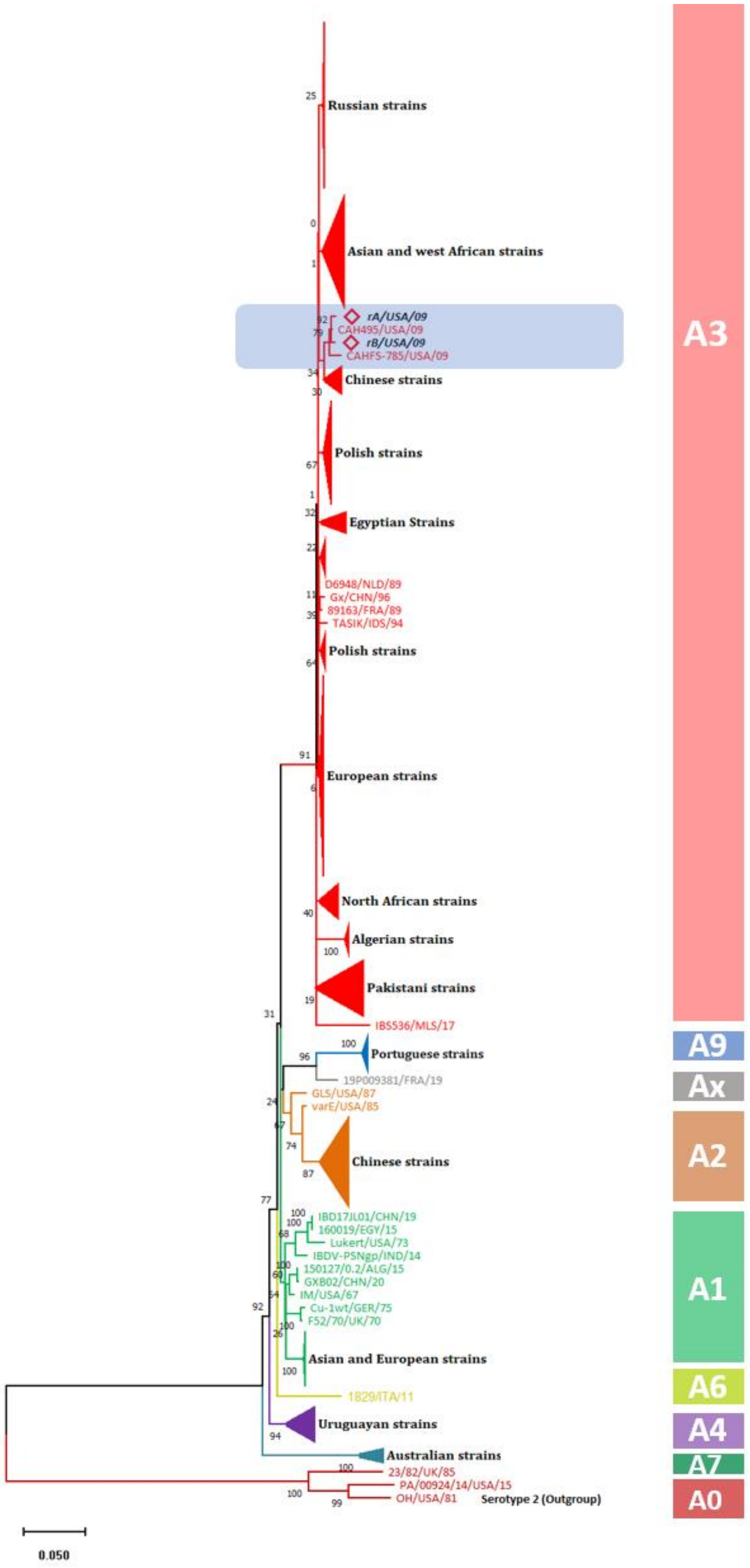

(a)

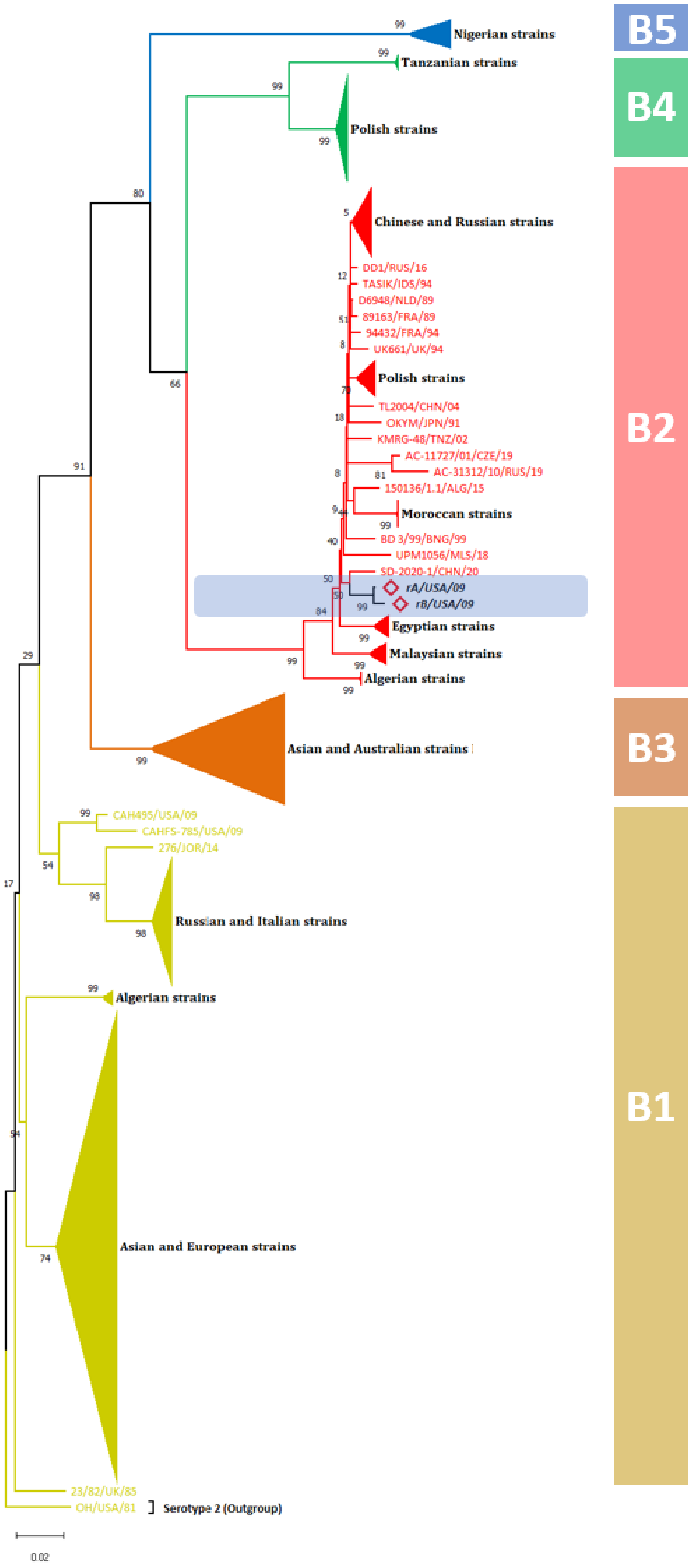

Nigerian strains
Tanzanian strains
Polish strains
Chinese and Russian strains
DD1/RUS/16
TASIK/IDS/94
D6948/NLD/89
89163/FRA/89
94432/FRA/94
UK661/UK/94
Polish strains
TL2004/CHN/04
OKYM/JPN/91
KMRG-48/TNZ/02
AC-11727/01/CZE/19
AC-31312/10/RUS/19
150136/1.1/ALG/15
Moroccan strains
BD 3/99/BNG/99
UPM1056/MLS/18
SD-2020-1/CHN/20
rA/USA/09
rB/USA/09
Egyptian strains
Malaysian strains
Algerian strains
Asian and Australian strains
CAH495/USA/09
CAHFS-785/USA/09
276/JOR/14
Russian and Italian strains
Algerian strains
Asian and European strains
23/82/UK/85
OH/USA/81
Serotype 2 (Outgroup)
0.02
B5
B4
B2
B3
B1

(b)

Figure S1. Compressed phylogenetic trees of the (a) truncated sequence of VP2 and (b) VP1 gene. Trees were inferred by using the Maximum Likelihood method and GTR + G + I model according to best fitting substitution model. Tree branches, not closely related to our isolates, were collapsed and named after the country of origin. Our isolates are denoted by italized bold black text with red diamond and highlighted in blue.

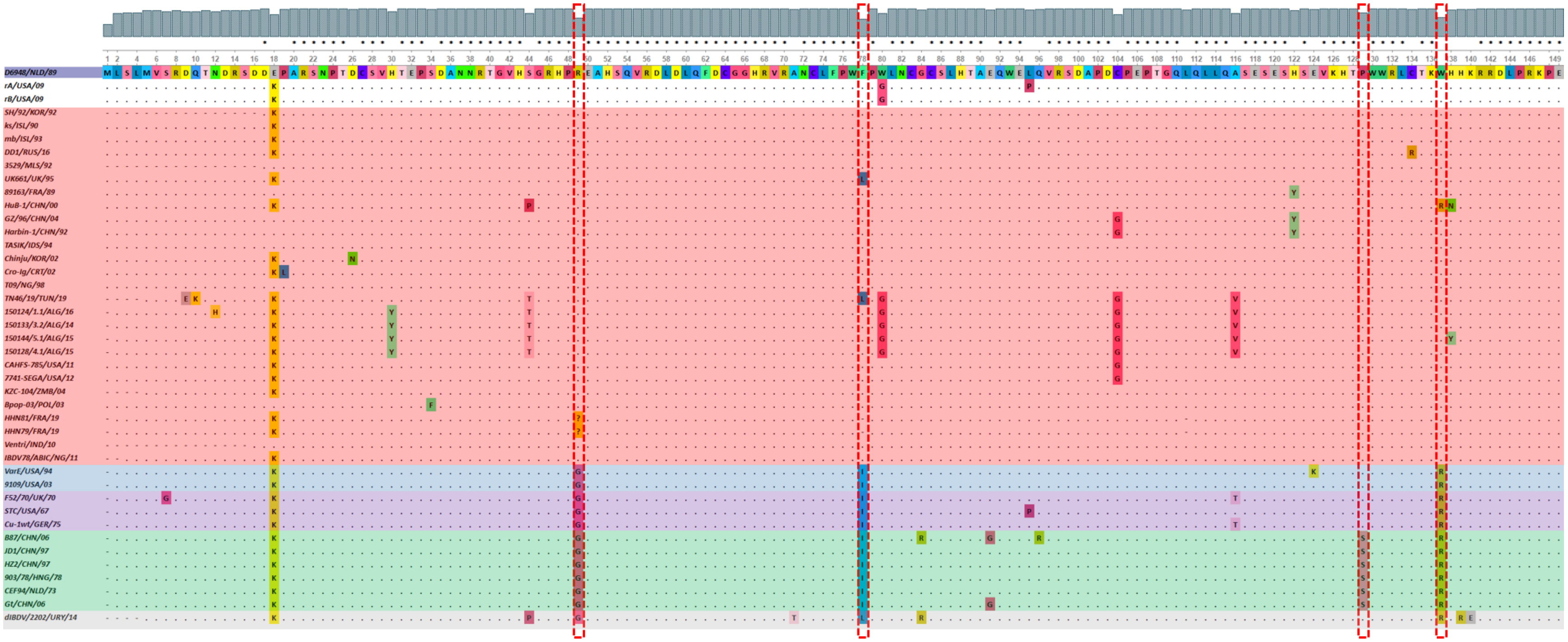

Figure S2. VP5 amino acid sequences of IBDV isolates (aa 1-149). Characteristic amino acids defining the vvIBDVs are boxed in dashed red lines. The highlights denote the different IBDV phenotypes (very virulent: red, variant: blue, classical: purple, attenuated: green and distinct: grey). D6948, the first sequence, refers to the reference vvIBDV strain used for comparison. Isolate nomenclature is expressed as isolate name/country of origin/collection year.

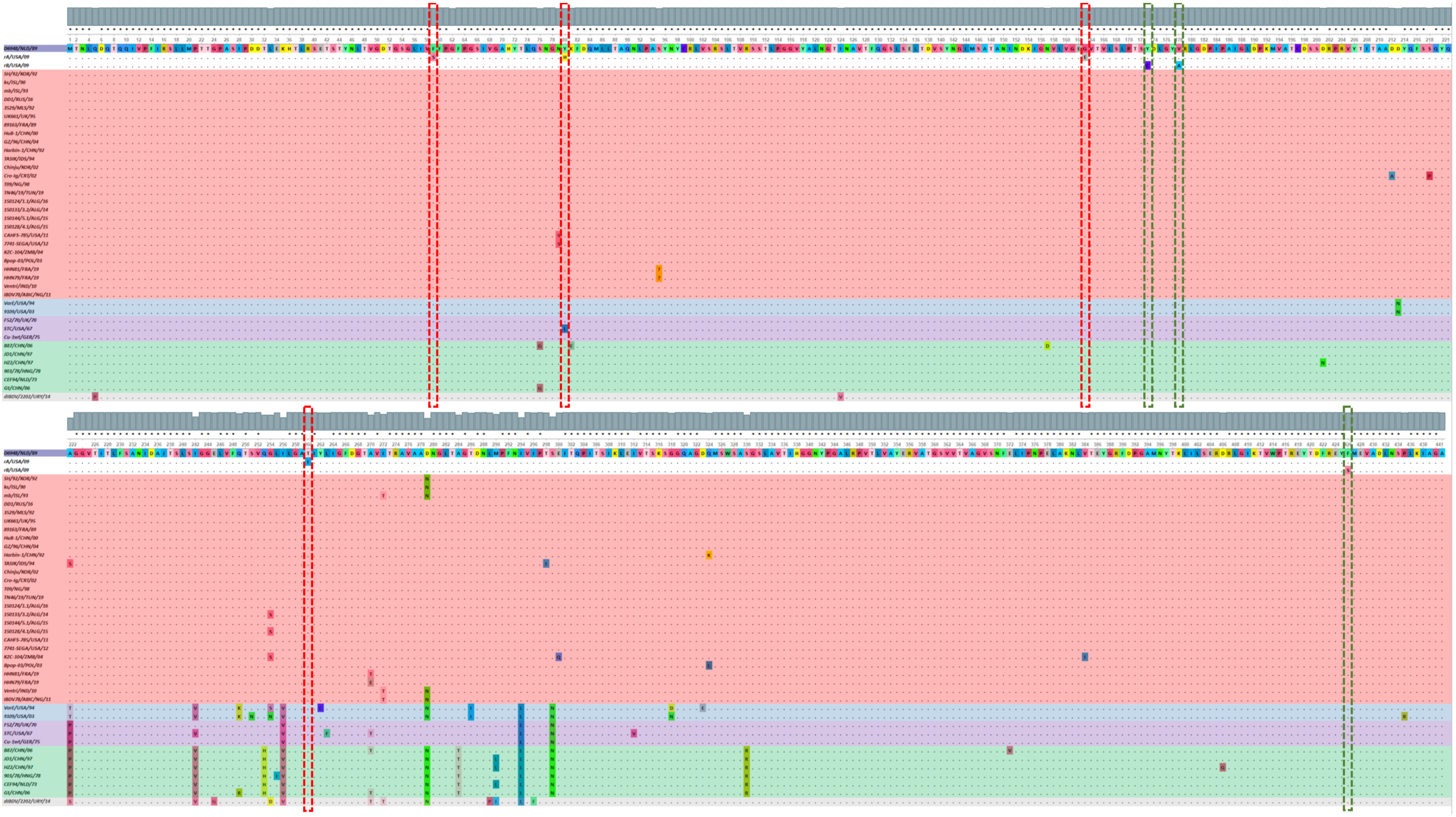

Figure S3. VP2 amino acid sequences of IBDV isolates (aa 1-441). Unique amino acid substitutions, aside from HVR, for rA and rB are boxed in dashed red and green lines, respectively. The highlights denote the different IBDV phenotypes (very virulent: red, variant: blue, classical: purple, attenuated: green and distinct: grey). D6948, the first sequence, refers to the reference vvIBDV strain.

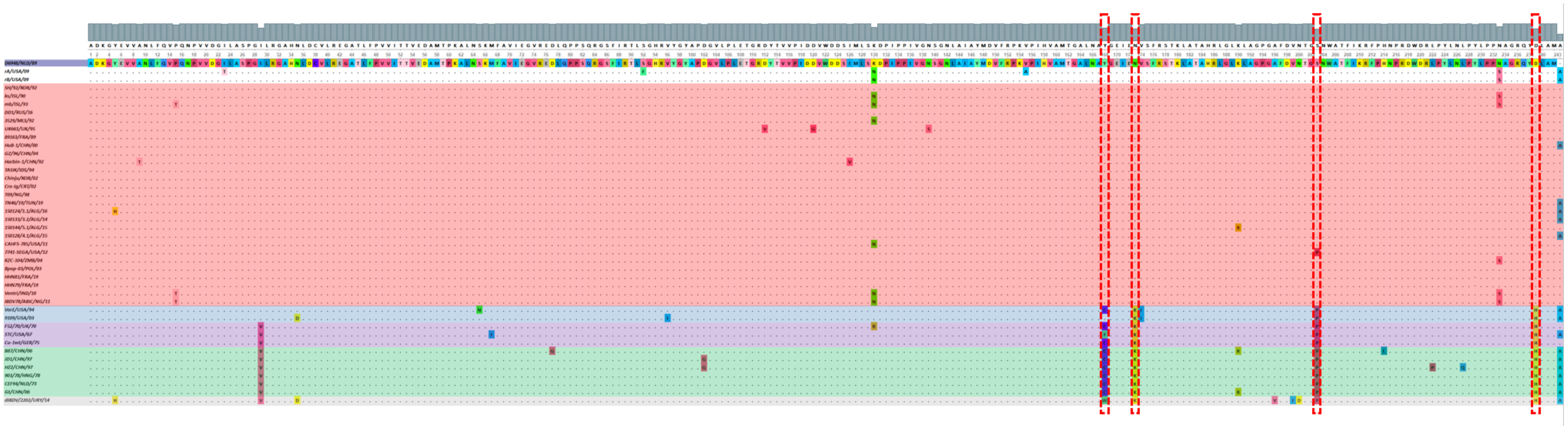

Figure S4. VP4 amino acid sequences of IBDV isolates (aa 1-243). Signature amino acids defining the vvIBDVs are boxed in dashed red lines. The highlights denote the different IBDV phenotypes (very virulent: red, variant: blue, classical: purple, attenuated: green and distinct: grey). D6948, the first sequence, refers to the reference vvIBDV strain.

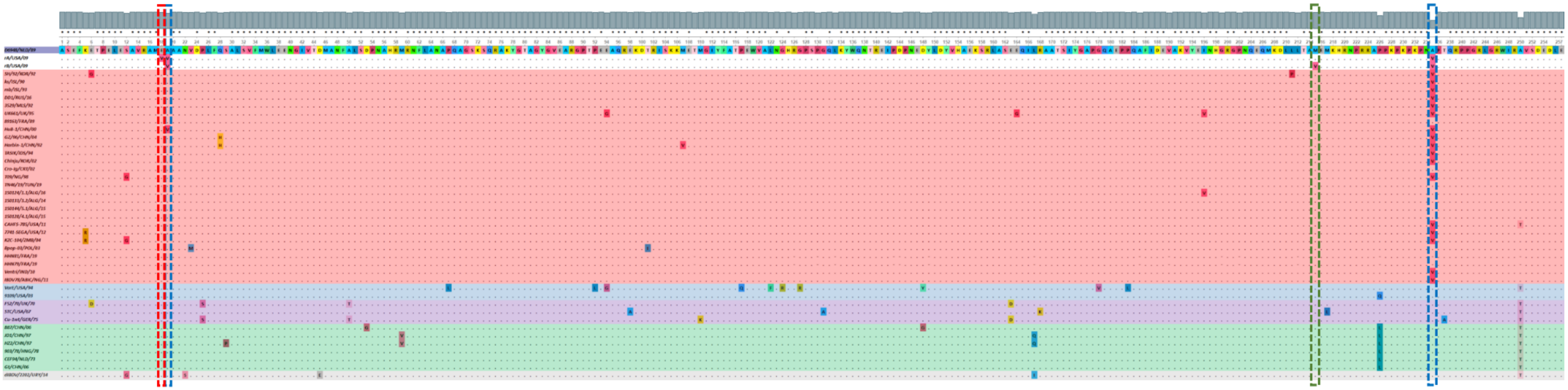

Figure S5. VP3 amino acid sequences of IBDV isolates (aa 1-257). Unique amino acid substitutions for rA and rB are boxed in dashed red and green lines, respectively. Amino acid substitution shared among rA, rB and vvIBDVs is marked by dashed blue line. The highlights denote the different IBDV phenotypes (very virulent: red, variant: blue, classical: purple, attenuated: green and distinct: grey). D6948, the first sequence, refers to the reference vvIBDV strain.

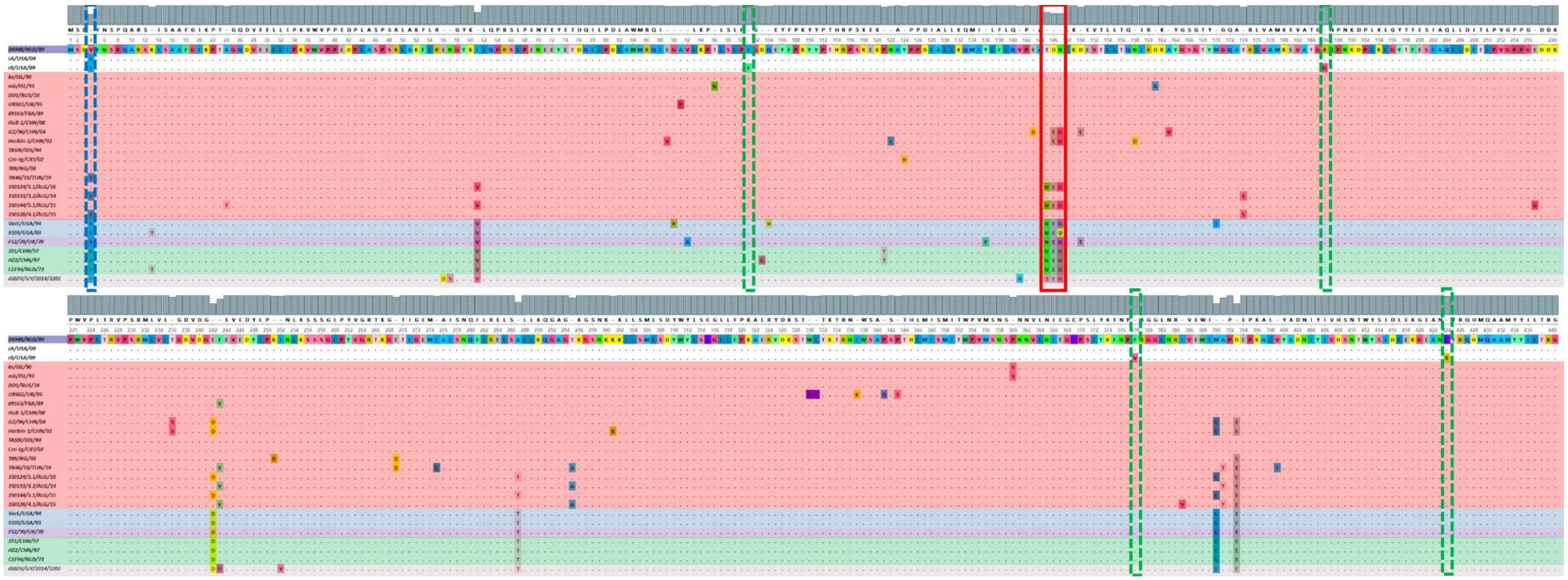

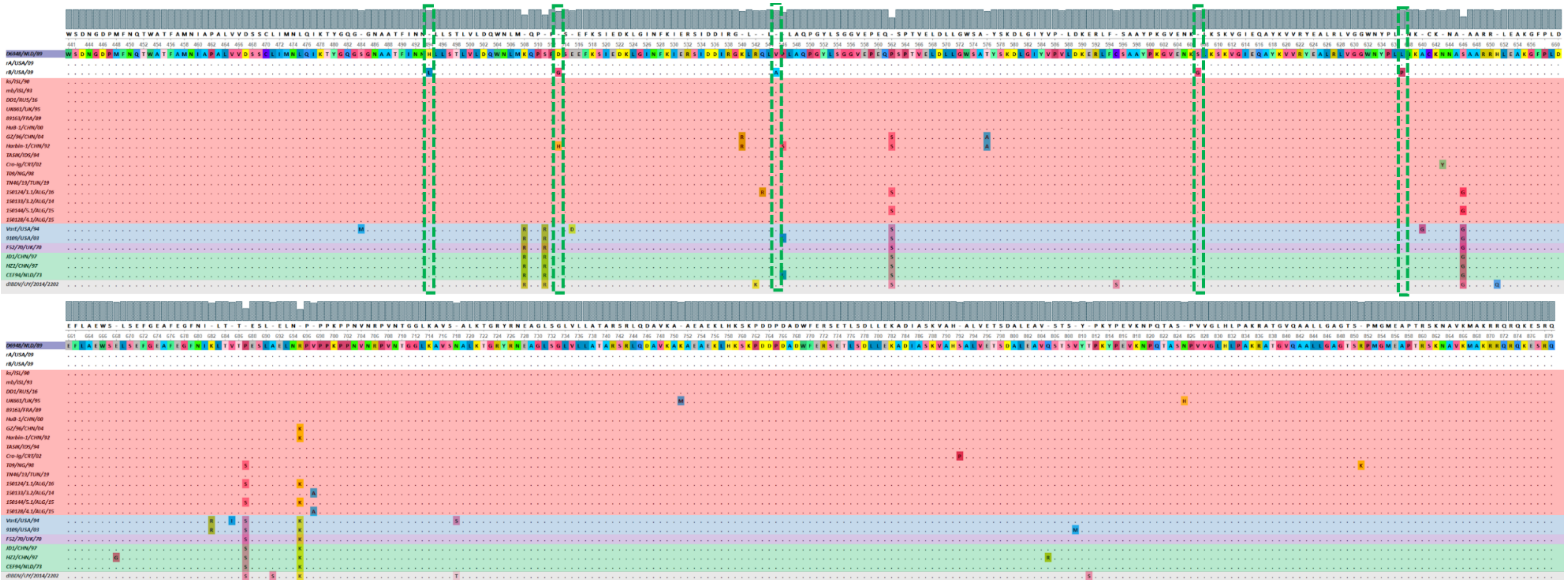

Figure S6. Aligned VP1 amino acid sequences of IBDV isolates (aa 1-879). Unique amino acid substitutions for rB are boxed in dashed green lines. Amino acid substitution shared among rA, rB and other IBDVs (belonging to various phenotypes) is marked by dashed blue line. The VP1 protein sequence TDN triplet (145-147), virulence signature, is displayed in red box. The highlights denote the different IBDV phenotypes (very virulent: red, variant: blue, classical: purple, attenuated: green and distinct: grey). D6948, the first sequence, refers to the reference vvIBDV strain.

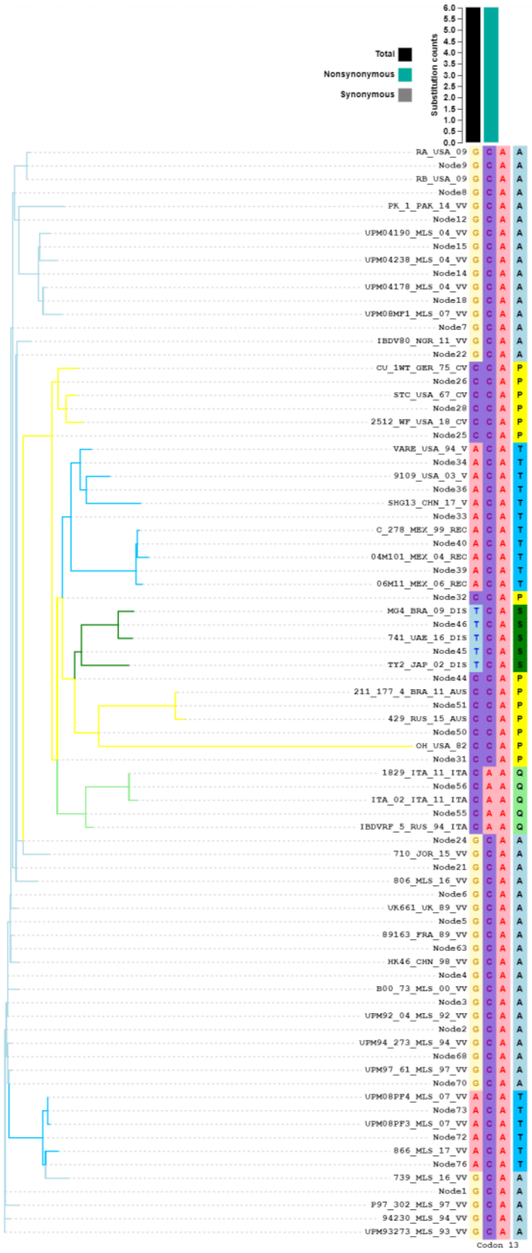

Figure S7. Codon-specific SLAC Phylogenetic Alignment tree showing aa positive selection across different sequences representing all IBDV phenotypes. The tree branch color is equivalent to the amino acid at this site (codon 13 in HVR, equivalent to codon 222 in VP2 and PP). The current study isolates rA and rB are the first two isolates in the tree. The sequences nomenclature is expressed as isolate name_country of origin_Collection year_Genogroup type (cv [Classical] for G1, v [Variant] for G2, vv [Very virulent] for G3, DIS [Distinct] for G4, REC [Recombinant] for G5, ITA [Italian] for G6 and AUS [Australian] for G7).

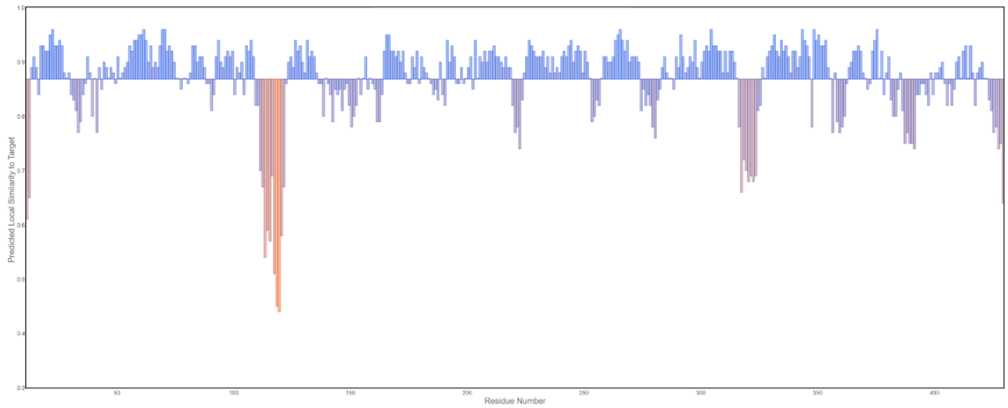

(a)

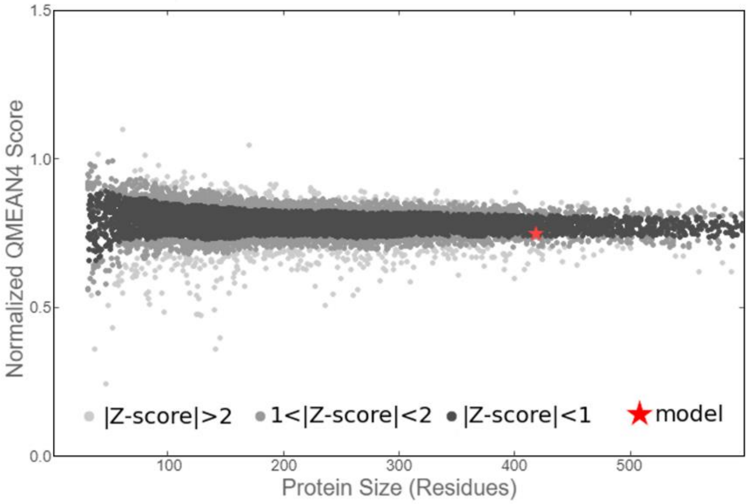

(b)

Figure S8. Quality estimation of VP2 protein modeling via (a) local quality estimate and (b) comparison with non-redundant set of PDB structures. QMEANDisCo Global is 0.87 ± 0.05 which indicates our model robustness.

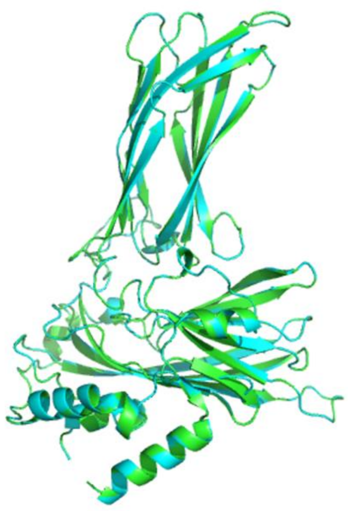

Figure S9. VP2 3D model alignment of both rA and rB isolates using Pymol. Alignment showed no molecular conformation changes since no looping or state change was noted from β-strand to helical structure and vice versa.

B. Tables

Table S1. Evolutionary divergence estimates between HVR sequences of IBDV isolates.

Species 1	Species 2	Distance (*d*)
**rA/USA/09**	**rB/USA/09**	**0.0024264**
**rB/USA/09**	**89163/FRA/89/vv**	**0.0171892**
rB/USA/09	B00/81/MLS/00/vv	0.0172288
rB/USA/09	94268/MLS/94/vv	0.0172421
rB/USA/09	UPM92-04/MLS/92/vv	0.0172553
**rA/USA/09**	**89163/FRA/89/vv**	**0.0195892**
rA/USA/09	B00/81/MLS/00/vv	0.0196288
rA/USA/09	94268/MLS/94/vv	0.0196421
rA/USA/09	UPM92-04/MLS/92/vv	0.0196553
rB/USA/09	UPM93273/MLS/93/vv	0.0196670
rB/USA/09	UK661/UK/89/vv	0.0220433
rB/USA/09	UPM97/61/MLS/97/vv	0.0220634
rA/USA/09	UPM93273/MLS/93/vv	0.0220670
rB/USA/09	HK46/CHN/98/vv	0.0220990
rA/USA/09	UK661/UK/89/vv	0.0244433
rA/USA/09	UPM97/61/MLS/97/vv	0.0244634
rB/USA/09	UPM94/273/MLS/94/vv	0.0244852
rA/USA/09	HK46/CHN/98/vv	0.0244990
rB/USA/09	94230/MLS/94/vv	0.0245113
rA/USA/09	UPM94/273/MLS/94/vv	0.0268852
rA/USA/09	94230/MLS/94/vv	0.0269113
rB/USA/09	B00/73/MLS/00/vv	0.0270187
rB/USA/09	IBDV80/NGR/11/vv	0.0271782
rA/USA/09	B00/73/MLS/00/vv	0.0294187
rA/USA/09	IBDV80/NGR/11/vv	0.0295782
rB/USA/09	P97/302/MLS/97/vv	0.0319135
rA/USA/09	P97/302/MLS/97/vv	0.0343135
rB/USA/09	806/MLS/16/vv	0.0346809
rA/USA/09	806/MLS/16/vv	0.0370809
rB/USA/09	UPM04178/MLS/04/vv	0.0500109
rA/USA/09	UPM04178/MLS/04/vv	0.0524109
rB/USA/09	UPM04190/MLS/04/vv	0.0524781
rB/USA/09	710/JOR/15/vv	0.0535659
rA/USA/09	UPM04190/MLS/04/vv	0.0548781
rB/USA/09	UPM08PF4/MLS/07/vv	0.0554881
rA/USA/09	710/JOR/15/vv	0.0559659
rB/USA/09	UPM04238/MLS/04/vv	0.0575892
rA/USA/09	UPM08PF4/MLS/07/vv	0.0578881
rB/USA/09	UPM08PF3/MLS/07/vv	0.0578883
rA/USA/09	UPM04238/MLS/04/vv	0.0599892
rA/USA/09	UPM08PF3/MLS/07/vv	0.0602883
rB/USA/09	PK-1/PAK/14/vv	0.0614246
rA/USA/09	PK-1/PAK/14/vv	0.0638246
rB/USA/09	UPM08MF1/MLS/07/vv	0.0653060
rA/USA/09	UPM08MF1/MLS/07/vv	0.0677060
rB/USA/09	866/MLS/17/vv	0.0680983
rA/USA/09	866/MLS/17/vv	0.0704983
rB/USA/09	739/MLS/16/vv	0.0808111
rA/USA/09	739/MLS/16/vv	0.0832111
rB/USA/09	STC/USA/67/Cv	0.0864294
rA/USA/09	STC/USA/67/Cv	0.0888294
rB/USA/09	Cu-1wt/GER/75/Cv	0.0912025
rB/USA/09	2512-WF/USA/18/Cv	0.0917685

rA/USA/09	Cu-1wt/GER/75/Cv	0.0936025
rA/USA/09	2512-WF/USA/18/Cv	0.0941685
rB/USA/09	IBDVRF-5/RUS/94/ITA	0.1002234
rB/USA/09	VarE/USA/94/v	0.1013795
rA/USA/09	IBDVRF-5/RUS/94/ITA	0.1026234
rA/USA/09	VarE/USA/94/v	0.1037795
rB/USA/09	9109/USA/03/v	0.1297360
rA/USA/09	9109/USA/03/v	0.1321360
rB/USA/09	TY2/JAP/02/DIS	0.1347998
rB/USA/09	1829/ITA/11/ITA	0.1353884
rB/USA/09	ITA-02/ITA/11/ITA	0.1362449
rA/USA/09	TY2/JAP/02/DIS	0.1371998
rA/USA/09	1829/ITA/11/ITA	0.1377884
rA/USA/09	ITA-02/ITA/11/ITA	0.1386449
rB/USA/09	MG4/BRA/09/DIS	0.1628463
rB/USA/09	SHG13/CHN/17/v	0.1639328
rB/USA/09	741/UAE/16/DIS	0.1649308
rA/USA/09	MG4/BRA/09/DIS	0.1652463
rB/USA/09	C-278/MEX/99/REC	0.1658685
rA/USA/09	SHG13/CHN/17/v	0.1663328
rB/USA/09	06M11/MEX/06/REC	0.1672374
rA/USA/09	741/UAE/16/DIS	0.1673308
rA/USA/09	C-278/MEX/99/REC	0.1682685
rA/USA/09	06M11/MEX/06/REC	0.1696374
rB/USA/09	04M101/MEX/04/REC	0.1736745
rA/USA/09	04M101/MEX/04/REC	0.1760745
rB/USA/09	211-177-4/BRA/11/AUS	0.2029601
rA/USA/09	211-177-4/BRA/11/AUS	0.2053601
rB/USA/09	429/RUS/15/AUS	0.2127904
rB/USA/09	V877-W/AUS/09/AUS	0.2127904
rA/USA/09	429/RUS/15/AUS	0.2151904
rA/USA/09	V877-W/AUS/09/AUS	0.2151904
rB/USA/09	OH/USA/82	0.7358425
rA/USA/09	OH/USA/82	0.7382425

rA and rB, displaying the closest evolutionary distance to each other, are shown in bold red. The isolate in bold black is the next closest to rA or rB.

Table S2. Best fitting model selection for VP2 HVR using Maximum Likelihood fits of 24 different nucleotide substitution models.

Model	BIC	AICc	lnL	I	G	R	FreqA	FreqT	FreqC	FreqG	A=>T	A=>C	A=>G	T=>A	T=>C	T=>G	C=>A	C=>T	C=>G	G=>A	G=>T	G=>C
**K2+G***	**6754.7**	6056.7	-2938.9	n/a	0.472	3.316	0.25	0.25	0.25	0.25	0.03	0.03	0.19	0.03	0.19	0.03	0.03	0.19	0.03	0.19	0.03	0.03
K2+G+I	6764.5	6058.6	-2938.9	0.069	0.541	3.344	0.25	0.25	0.25	0.25	0.03	0.03	0.19	0.03	0.19	0.03	0.03	0.19	0.03	0.19	0.03	0.03
T92+G	6766.8	6061.0	-2940.0	n/a	0.469	3.332	0.239	0.239	0.261	0.261	0.03	0.03	0.2	0.03	0.2	0.03	0.03	0.18	0.03	0.18	0.03	0.03
GTR+G	6770.7	6017.9	-2912.4	n/a	0.453	3.271	0.271	0.208	0.273	0.248	0.05	0.04	0.17	0.06	0.23	0.01	0.04	0.18	0.01	0.19	0.01	0.01
HKY+G	6771.9	6050.4	-2932.7	n/a	0.461	3.406	0.271	0.208	0.273	0.248	0.02	0.03	0.19	0.03	0.21	0.03	0.03	0.16	0.03	0.21	0.02	0.03
T92+G+I	6776.4	6062.8	-2939.9	0.065	0.537	3.344	0.239	0.239	0.261	0.261	0.03	0.03	0.2	0.03	0.2	0.03	0.03	0.18	0.03	0.18	0.03	0.03
TN93+G	6779.7	6050.3	-2931.7	n/a	0.464	3.390	0.271	0.208	0.273	0.248	0.02	0.03	0.17	0.03	0.24	0.03	0.03	0.18	0.03	0.19	0.02	0.03
GTR+G+I	6780.0	6019.3	-2912.2	0.057	0.509	3.282	0.271	0.208	0.273	0.248	0.05	0.04	0.17	0.06	0.23	0.01	0.04	0.18	0.01	0.19	0.01	0.01
HKY+G+I	6781.6	6052.3	-2932.7	0.062	0.524	3.423	0.271	0.208	0.273	0.248	0.02	0.03	0.19	0.03	0.21	0.03	0.03	0.16	0.03	0.21	0.02	0.03
TN93+G+I	6789.5	6052.3	-2931.7	0.053	0.518	3.402	0.271	0.208	0.273	0.248	0.02	0.03	0.17	0.03	0.24	0.03	0.03	0.18	0.03	0.19	0.02	0.03
K2+I	6807.0	6109.0	-2965.1	0.438	n/a	3.134	0.250	0.250	0.250	0.250	0.03	0.03	0.19	0.03	0.19	0.03	0.03	0.19	0.03	0.19	0.03	0.03
T92+I	6818.8	6113.0	-2966.1	0.438	n/a	3.145	0.239	0.239	0.261	0.261	0.03	0.03	0.2	0.03	0.2	0.03	0.03	0.18	0.03	0.18	0.03	0.03
HKY+I	6822.2	6100.7	-2957.9	0.440	n/a	3.207	0.271	0.208	0.273	0.248	0.02	0.03	0.19	0.03	0.21	0.03	0.03	0.16	0.03	0.21	0.02	0.03
TN93+I	6828.3	6098.9	-2956.0	0.441	n/a	3.233	0.271	0.208	0.273	0.248	0.02	0.03	0.17	0.03	0.24	0.03	0.03	0.18	0.03	0.18	0.02	0.03
GTR+I	6849.3	6096.5	-2951.7	0.435	n/a	2.349	0.271	0.208	0.273	0.248	0.04	0.04	0.17	0.06	0.2	0.03	0.04	0.15	0.03	0.19	0.02	0.03
K2	6978.7	6288.5	-3055.8	n/a	n/a	2.672	0.250	0.250	0.250	0.250	0.03	0.03	0.18	0.03	0.18	0.03	0.03	0.18	0.03	0.18	0.03	0.03
T92	6992.3	6294.3	-3057.7	n/a	n/a	2.673	0.239	0.239	0.261	0.261	0.03	0.04	0.19	0.03	0.19	0.04	0.03	0.17	0.04	0.17	0.03	0.04
HKY	7000.8	6287.1	-3052.1	n/a	n/a	2.682	0.271	0.208	0.273	0.248	0.03	0.04	0.18	0.04	0.2	0.03	0.04	0.15	0.03	0.2	0.03	0.04
TN93	7007.3	6285.8	-3050.5	n/a	n/a	2.684	0.271	0.208	0.273	0.248	0.03	0.04	0.16	0.04	0.22	0.03	0.04	0.17	0.03	0.18	0.03	0.04
GTR	7023.6	6278.6	-3043.8	n/a	n/a	2.094	0.271	0.208	0.273	0.248	0.04	0.05	0.16	0.05	0.19	0.03	0.05	0.14	0.03	0.18	0.02	0.03
JC+G	7065.4	6375.3	-3099.2	n/a	0.543	0.5	0.25	0.25	0.25	0.25	0.08	0.08	0.08	0.08	0.08	0.08	0.08	0.08	0.08	0.08	0.08	0.08
JC+G+I	7075.1	6377.1	-3099.1	0.087	0.656	0.5	0.25	0.25	0.25	0.25	0.08	0.08	0.08	0.08	0.08	0.08	0.08	0.08	0.08	0.08	0.08	0.08
JC+I	7109.1	6418.9	-3121.0	0.427	n/a	0.5	0.25	0.25	0.25	0.25	0.08	0.08	0.08	0.08	0.08	0.08	0.08	0.08	0.08	0.08	0.08	0.08
JC	7258.0	6575.7	-3200.4	n/a	n/a	0.5	0.25	0.25	0.25	0.25	0.08	0.08	0.08	0.08	0.08	0.08	0.08	0.08	0.08	0.08	0.08	0.08

*Model with the lowest BIC scores (Bayesian Information Criterion) is considered to best describe the substitution pattern (shown in bold red). For each model, AICc value (Akaike Information Criterion, corrected), Maximum Likelihood value (lnL), and the number of parameters (including branch lengths) are also depicted above. 5 rate categories-discrete Gamma distribution (+G) was applied for modeling the evolutionary rates non-uniformity among sites and supposing that a certain fraction of sites is evolutionarily invariable (+I). Proposed or estimated values of transition/transversion bias (R) for each model are displayed. They are followed by nucleotide frequencies (f) and rates of base substitutions (r) for each nucleotide pair. A total of 435 positions existed in the final dataset. Abbreviations: K2: Kimura 2-parameter; TR: General Time Reversible; HKY: Hasegawa-Kishino-Yano; TN93: Tamura-Nei; T92: Tamura 3-parameter; JC: Jukes-Cantor.

Table S3. Accession numbers of sequences used for phylogenetic analysis of IBDV HVR for genotyping.

Accession number	Isolate	Country	Year	Classification	Abbreviated name*	Genogroup
AF362747	Cu-1 wt	Germany	1975	Classical	Cu-1wt/GER/75/Cv	1
D00499	STC	USA	1967	Classical	STC/USA/67/Cv	1
MH329181	2512 Winterfield	USA	2018	Classical	2512-WF/USA/18/Cv	1
AF133904	Var E	USA	1994	Variant	VarE/USA/94/v	2
AY462027	9109	USA	2003	Variant	9109/USA/03/v	2
MH879090	SHG13	China	2017	Variant	SHG13/CHN/17/v	2
GQ131541	UPM08PF4	Malaysia	2007	Very virulent	UPM08PF4/MLS/07/vv	3
GQ131540	UPM08PF3	Malaysia	2007	Very virulent	UPM08PF3/MLS/07/vv	3
MF142587	866	Malaysia	2017	Very virulent	866/MLS/07/vv	3
MF142568	739	Malaysia	2016	Very virulent	739/MLS/16/vv	3
AF247006	UPM97/61	Malaysia	1997	Very virulent	UPM97/61/MLS/97/vv	3
AF248612	UPM94/273	Malaysia	1994	Very virulent	UPM94/273/MLS/94/vv	3
AY245550	UPM93273	Malaysia	1993	Very virulent	UPM93273/MLS/93/vv	3
AY520911	94230	Malaysia	1994	Very virulent	94230/MLS/94/vv	3
AF464901	P97/302	Malaysia	1997	Very virulent	P97/302/MLS/97/vv	3
AY520910	B00/81	Malaysia	2000	Very virulent	B00/81/MLS/00/vv	3
AF262030	UPM92-04	Malaysia	1992	Very virulent	UPM92-04/MLS/92/vv	3
AY333088	94268	Malaysia	1994	Very virulent	94268/MLS/94/vv	3
AY520909	B00/73	Malaysia	2000	Very virulent	B00/73/MLS/00/vv	3
AY791998	UPM04190	Malaysia	2004	Very virulent	UPM04190/MLS/04/vv	3
DQ000436	UPM04238	Malaysia	2004	Very virulent	UPM04238/MLS/04/vv	3
AY970665	UPM04178	Malaysia	2004	Very virulent	UPM04178/MLS/04/vv	3
GQ131544	UPM08MF1	Malaysia	2007	Very virulent	UPM08MF1/MLS/07/vv	3
HG974563	89163	France	1989	Very virulent	89163/FRA/89/vv	3
AF092943	HK46	China	1998	Very virulent	HK46/CHN/98/vv	3
NC_004178	UK661	UK	1989	Very virulent	UK661/UK/89/vv	3
KT281984	PK-1	Pakistan	2014	Very virulent	PK-1/PAK/14/vv	3
MF142584	806	Malaysia	2016	Very virulent	806/MLS/16/vv	3
JX424079	IBDV80	Nigeria	2011	Very virulent	IBDV80/NGR/11/vv	3
MF142560	710	Jordan	2015	Very virulent	710/JOR/15/vv	3
JN982252	MG4	Brazil	2009	distinct	MG4/BRA/09/DIS	4
MF142569	741	UAE	2016	distinct	741/UAE/16/DIS	4
LC136880	TY2	Japan	2002	distinct	TY2/JAP/02/DIS	4
JQ277695	06M11	Mexico	2006	Recombinant	06M11/MEX/06/REC	5
AF498627	C-278	Mexico	1999	Recombinant	C-278/MEX/99/REC	5
DQ916210	04M101	Mexico	2004	Recombinant	04M101/MEX/04/REC	5
Z97002	IBDVRF-5	Russia	1994	Italian	IBDVRF-5/RUS/94/ITA	6
KY930929	1829	Italy	2011	Italian	1829/ITA/11/ITA	6
JN852986	ITA-02	Italy	2011	Italian	ITA-02/ITA/11/ITA	6
KY612971	211-177-4	Brazil	2011	Australian	211-177-4/BRA/11/AUS	7
MF142536	429	Russia	2015	Australian	429/RUS/15/AUS	7
HM071991	V877-W	Australia	2009	Australian	V877-W/AUS/09/AUS	7
U30818	OH	USA	1982	Serotype 2	OH/USA/82	N/A

*Abbreviated name followed the order of Isolate_name/Country_of_origin/Collection_year/Classification.

Table S4. Accession numbers of sequences used for amino acid sequence analysis of VP2-HVR.

Accession number	Isolate	Abbreviated name*	Country	Year	Phenotype
DQ927040	mb	mb/ISL/93	Israel	2008	very virulent
JX424077	IBDV78/ABIC	IBDV78/ABIC/NG/11	Nigeria	2011	very virulent
KJ547670	Ventri-IBDV-Plus	Ventri/IND/10	India	2010	very virulent
DQ927042	ks	ks/ISL/90	Israel	1990	very virulent
AF533670	SH/92	SH/92/KOR/92	South Korea	1992	very virulent
KF569805	HuB-1	HuB-1/CHN/00	China	2000	Very virulent
AY099456	T09 (NIE009t)	T09/NG/98	Nigeria	1998	Very virulent
AF508176	Chinju	Chinju/KOR/02	South Korea	2002	Very virulent
KC189836	3529/92	3529/MLS/92	Malaysia	1992	Very virulent
MH644846	DD1	DD1/RUS/16	Russia	2016	Very virulent
AF322444	TASIK	TASIK/IDS/94	Indonesia	1994	Very virulent
HG974563	89163	89163/FRA/89	France	1989	Very virulent
AF240686	D6948	D6948/NLD/89	Netherlands	1989	Very virulent
JF907702	CAHFS-785-SESW	CAHFS-785/USA/11	USA	2011	Very virulent (Reassortant)
AY598356	GZ/96	GZ/96/CHN/04	China	2004	Very virulent
EF517528	Harbin-1	Harbin-1/CHN/92	China	1992	Very virulent
EU184685	Cro-Ig/02	Cro-Ig/CRT/02	Croatia	2002	Very virulent
AB368968	KZC-104	KZC-104/ZMB/04	Zambia	2004	Very virulent (Reassortant)
JQ403646	7741-SEGA-SESW	7741-SEGA/USA/12	USA	2012	Very virulent (Reassortant)
MF969105	150124/1.1	150124/1.1/ALG/16	Algeria	2016	Very virulent
X92760	UK661	UK661/UK/95	UK	1995	Very virulent
MH545934	Bpop/03	Bpop-03/POL/03	Poland	2003	Very virulent (Reassortant)
MN480311	TN46/19	TN46/19/TUN/19	Tunisia	2019	Very virulent
MF969109	150128/4.1	150128/4.1/ALG/15	Algeria	2015	Very virulent
MF969111	150133/3.2	150133/3.2/ALG/14	Algeria	2014	Very virulent
MF969115	150144/5.1	150144/5.1/ALG/15	Algeria	2015	Very virulent
MT935590	*HHN79*	*HHN79/FRA/19*	*France*	*2019*	*Very virulent (Recombinant)*
MT935589	*HHN81*	*HHN81/FRA/19*	*France*	*2019*	*Very virulent (Recombinant)*
AF133904	Variant E	VarE/USA/94	USA	1994	Variant
AY462027	9109	9109/USA/03	USA	2003	Variant
HG974565	Faragher 52/70	F52/70/UK/70	UK	1970	Classical
AF362747	Cu-1wt	Cu-1wt/GER/75	Germany	1975	Classical
D00499	STC	STC/USA/67	USA	1967	Classical
DQ403248	Gt	Gt/CHN/06	China	2006	Attenuated
JQ411012	903/78	903/78/HNG/78	Hungary	1978	Attenuated
AF194428	CEF94	CEF94/NLD/73	Netherlands	1973	Attenuated
AF321055	JD1	JD1/CHN/97	China	1997	Attenuated
DQ906921	B87	B87/CHN/06	China	2006	Attenuated

AF321054	HZ2	HZ2/CHN/97	China	1997	Attenuated
KT336459	dIBDV/UY/2014/2202	dIBDV/2202/URY/14	Uruguay	2014	Distinct
U30818	OH	OH/USA/82	USA	1982	Serotype 2

*Abbreviated name followed the order of Isolate_name/Country_of_origin/Collection_year.

Table S5. Evolutionary divergence estimates between mature VP2 sequences of the study isolates and other IBDV isolates.

Species 1	Species 2	Distance (*d*)
**rB/USA/09**	**CAH495/USA/09**	**0.00330351**
**rA/USA/09**	**CAH495/USA/09**	**0.00413566**
**rA/USA/09**	**rB/USA/09**	**0.00584559**
rB/USA/09	CAHFS-785/USA/09	0.01349633
rB/USA/09	D6948/NLD/89	0.01418829
rA/USA/09	CAHFS-785/USA/09	0.01433059
rB/USA/09	LJ-5/CHN/18	0.01433116
rB/USA/09	ks/ISL/90	0.01501753
rA/USA/09	D6948/NLD/89	0.01502255
rA/USA/09	LJ-5/CHN/18	0.01516542
rA/USA/09	ks/ISL/90	0.01585179
rB/USA/09	SD-2013-1/CHN/13	0.01606253
rB/USA/09	89163/FRA/89	0.01666394
rB/USA/09	mb/ISL/08	0.01666549
rB/USA/09	735/RUS/15	0.01666655
rB/USA/09	711/RUS/15	0.01666760
rB/USA/09	731/RUS/15	0.01666866
rB/USA/09	727/RUS/15	0.01666971
rB/USA/09	713/RUS/15	0.01666971
rB/USA/09	UK661/UK/94	0.01667802
rA/USA/09	SD-2013-1/CHN/13	0.01689679
rA/USA/09	89163/FRA/89	0.01749820
rA/USA/09	mb/ISL/08	0.01749975
rA/USA/09	735/RUS/15	0.01750081
rA/USA/09	711/RUS/15	0.01750186
rA/USA/09	731/RUS/15	0.01750292
rA/USA/09	727/RUS/15	0.01750397
rA/USA/09	713/RUS/15	0.01750397
rA/USA/09	UK661/UK/94	0.01751228
rB/USA/09	DD1/RUS/16	0.01751885
rB/USA/09	S18/CHN/18	0.01781649
rB/USA/09	OKYM/JPN/91	0.01831884
rB/USA/09	Gx/CHN/96	0.01833738
rA/USA/09	DD1/RUS/16	0.01835311
rB/USA/09	236/POL/14	0.01853086
rB/USA/09	176/POL/14	0.01853086
rA/USA/09	S18/CHN/18	0.01865075
rA/USA/09	OKYM/JPN/91	0.01915310
rA/USA/09	Gx/CHN/96	0.01917164
rB/USA/09	75/11/POL/11	0.01917623
rB/USA/09	SH19/CHN/19	0.01923232
rA/USA/09	236/POL/14	0.01936512
rA/USA/09	176/POL/14	0.01936512
rB/USA/09	72/POL/15	0.01941053
rB/USA/09	20/POL/15	0.01941053
rA/USA/09	75/11/POL/11	0.02001049
rA/USA/09	SH19/CHN/19	0.02006658
rA/USA/09	72/POL/15	0.02024479
rA/USA/09	20/POL/15	0.02024479
rB/USA/09	02015.1/VNZ/02	0.02081021
rB/USA/09	94432/FRA/94	0.02081943
rB/USA/09	TASIK/IDS/94	0.02086795
rB/USA/09	KMRG-00/TNZ/01	0.02095573

rB/USA/09	Bug/03/POL/03	0.02099715
rA/USA/09	02015.1/VNZ/02	0.02164447
rA/USA/09	94432/FRA/94	0.02165369
rA/USA/09	TASIK/IDS/94	0.02170221
rA/USA/09	KMRG-00/TNZ/01	0.02178999
rA/USA/09	Bug/03/POL/03	0.02183141
rB/USA/09	KDSM-02/TNZ/04	0.02278079
rB/USA/09	SD-2020-1/CHN/20	0.02295123
rA/USA/09	KDSM-02/TNZ/04	0.02361505
rB/USA/09	83/POL/11	0.02367185
rA/USA/09	SD-2020-1/CHN/20	0.02378549
rB/USA/09	131/POL/14	0.02384115
rB/USA/09	BD 3/99/BNG/99	0.02427248
rB/USA/09	150136/1.1/ALG/15	0.02431756
rA/USA/09	83/POL/11	0.02450611
rB/USA/09	98/POL/13	0.02455778
rA/USA/09	131/POL/14	0.02467541
rB/USA/09	96/POL/15	0.02472323
rA/USA/09	BD 3/99/BNG/99	0.02510674
rA/USA/09	150136/1.1/ALG/15	0.02515182
rA/USA/09	98/POL/13	0.02539204
rA/USA/09	96/POL/15	0.02555749
rB/USA/09	200/POL/15	0.02561007
rB/USA/09	li4129/FIN/14	0.02599223
rB/USA/09	189/POL/14	0.02633552
rA/USA/09	200/POL/15	0.02644433
rB/USA/09	123/POL/15	0.02649219
rA/USA/09	li4129/FIN/14	0.02682649
rB/USA/09	170W/POL/14	0.02685036
rA/USA/09	189/POL/14	0.02716978
rB/USA/09	216/POL/14	0.02722099
rB/USA/09	115/14A/POL/14	0.02722099
rA/USA/09	123/POL/15	0.02732645
rB/USA/09	115/14B/POL/14	0.02768103
rB/USA/09	117/14/POL/14	0.02768103
rA/USA/09	170W/POL/14	0.02768462
rA/USA/09	216/POL/14	0.02805525
rA/USA/09	115/14A/POL/14	0.02805525
rB/USA/09	217/POL/13	0.02814672
rA/USA/09	115/14B/POL/14	0.02851529
rA/USA/09	117/14/POL/14	0.02851529
rA/USA/09	217/POL/13	0.02898098
rB/USA/09	KMRG-48/TNZ/02	0.03088346
rA/USA/09	KMRG-48/TNZ/02	0.03171772
rB/USA/09	NKL14/IND/14	0.03195883
rB/USA/09	RPM14/IND/14	0.03204899
rB/USA/09	64/MOR/18	0.03243174
rB/USA/09	8/MOR/17	0.03243279
rB/USA/09	9-1/MOR/17	0.03243385
rB/USA/09	61-7/MOR/18	0.03243385
rA/USA/09	NKL14/IND/14	0.03279309
rA/USA/09	RPM14/IND/14	0.03288325
rA/USA/09	64/MOR/18	0.03326600
rA/USA/09	8/MOR/17	0.03326705
rA/USA/09	9-1/MOR/17	0.03326811
rA/USA/09	61-7/MOR/18	0.03326811
rB/USA/09	IBD13HeB01/CHN/13	0.03402501

rB/USA/09	HLJ-0504/CHN/05	0.03482381
rA/USA/09	IBD13HeB01/CHN/13	0.03485927
rB/USA/09	PLATEAU7/NG/09	0.03559690
rB/USA/09	THCU07/TAI/17	0.03560670
rA/USA/09	HLJ-0504/CHN/05	0.03565807
rA/USA/09	PLATEAU7/NG/09	0.03643116
rA/USA/09	THCU07/TAI/17	0.03644096
rB/USA/09	160021/EGY/15	0.03687741
rB/USA/09	EDE14/IND/14	0.03749767
rA/USA/09	160021/EGY/15	0.03771167
rB/USA/09	160023/EGY/15	0.03781240
rA/USA/09	EDE14/IND/14	0.03833193
rA/USA/09	160023/EGY/15	0.03864666
rB/USA/09	BGE15/IND/15	0.04027283
rB/USA/09	UVC1/NG/19	0.04055561
rB/USA/09	THI14/IND/14	0.04094352
rB/USA/09	a893/GER/21	0.04099119
rB/USA/09	150124/1.1/ALG/16	0.04099205
rB/USA/09	b507/GER/21	0.04101011
rA/USA/09	BGE15/IND/15	0.04110709
rA/USA/09	UVC1/NG/19	0.04138987
rA/USA/09	THI14/IND/14	0.04177778
rA/USA/09	a893/GER/21	0.04182545
rA/USA/09	150124/1.1/ALG/16	0.04182631
rA/USA/09	b507/GER/21	0.04184437
rB/USA/09	IBS624/MLS/17	0.04218095
rB/USA/09	AT6/NG/19	0.04234487
rB/USA/09	D3996/1/BEL/17	0.04266148
rB/USA/09	D4051/1/SWD/17	0.04267137
rB/USA/09	BAUCHI37/NG/12	0.04284206
rA/USA/09	IBS624/MLS/17	0.04301521
rB/USA/09	b335/NLD/21	0.04305677
rB/USA/09	150133/3.2/ALG/14	0.04317208
rA/USA/09	AT6/NG/19	0.04317913
rA/USA/09	D3996/1/BEL/17	0.04349574
rA/USA/09	D4051/1/SWD/17	0.04350563
rA/USA/09	BAUCHI37/NG/12	0.04367632
rA/USA/09	b335/NLD/21	0.04389103
rB/USA/09	150128/4.1/ALG/15	0.04399961
rA/USA/09	150133/3.2/ALG/14	0.04400634
rB/USA/09	150144/5.1/ALG/15	0.04401040
rB/USA/09	D4320/6/DEN/18	0.04434321
rB/USA/09	D4389/1/SWD/18	0.04455946
rA/USA/09	150128/4.1/ALG/15	0.04483387
rA/USA/09	150144/5.1/ALG/15	0.04484466
rB/USA/09	b524/FRA/21	0.04513549
rB/USA/09	b317/FRA/21	0.04513549
rA/USA/09	D4320/6/DEN/18	0.04517747
rA/USA/09	D4389/1/SWD/18	0.04539372
rB/USA/09	D3976/1/GER/17	0.04557263
rB/USA/09	BGE14/IND/15	0.04564943
rA/USA/09	b524/FRA/21	0.04596975
rA/USA/09	b317/FRA/21	0.04596975
rB/USA/09	UPM766/MLS/18	0.04611931
rB/USA/09	276/JOR/14	0.04640021
rA/USA/09	D3976/1/GER/17	0.04640689
rB/USA/09	AC-09389/02/IRL/19	0.04647996

rA/USA/09	BGE14/IND/15	0.04648369
rB/USA/09	D4628/2/CZE/19	0.04649274
rA/USA/09	UPM766/MLS/18	0.04695357
rB/USA/09	4439001/BEL/20	0.04706962
rB/USA/09	b292/FRA/21	0.04719234
rA/USA/09	276/JOR/14	0.04723447
rA/USA/09	AC-09389/02/IRL/19	0.04731422
rA/USA/09	D4628/2/CZE/19	0.04732700
rB/USA/09	25/11/LTV/11	0.04774138
rA/USA/09	4439001/BEL/20	0.04790388
rA/USA/09	b292/FRA/21	0.04802660
rB/USA/09	D3994/1/8/NLD/17	0.04831410
rA/USA/09	25/11/LTV/11	0.04857564
rA/USA/09	D3994/1/8/NLD/17	0.04914836
rB/USA/09	a833/PRT/21	0.04926792
rB/USA/09	a749/FRA/21	0.04926898
rB/USA/09	a524/UK/21	0.04933266
rA/USA/09	a833/PRT/21	0.05010218
rA/USA/09	a749/FRA/21	0.05010324
rA/USA/09	a524/UK/21	0.05016692
rB/USA/09	a227/UK/21	0.05122511
rB/USA/09	a452/UK/21	0.05122590
rB/USA/09	b542/FRA/21	0.05135761
rB/USA/09	b289/UK/21	0.05152855
rB/USA/09	a664/DEN/21	0.05164983
rA/USA/09	a227/UK/21	0.05205937
rA/USA/09	a452/UK/21	0.05206016
rB/USA/09	224/RUS/14	0.05212103
rB/USA/09	189/RUS/14	0.05212209
rB/USA/09	232/RUS/15	0.05212314
rA/USA/09	b542/FRA/21	0.05219187
rB/USA/09	UPM1056/MLS/18	0.05235887
rA/USA/09	b289/UK/21	0.05236281
rB/USA/09	774/KAZ/16	0.05240150
rA/USA/09	a664/DEN/21	0.05248409
rA/USA/09	224/RUS/14	0.05295529
rA/USA/09	189/RUS/14	0.05295635
rA/USA/09	232/RUS/15	0.05295740
rB/USA/09	b630/UK/21	0.05312964
rA/USA/09	UPM1056/MLS/18	0.05319313
rA/USA/09	774/KAZ/16	0.05323576
rB/USA/09	437/RUS/15	0.05362600
rB/USA/09	593/RUS/15	0.05362706
rB/USA/09	590/RUS/15	0.05362706
rA/USA/09	b630/UK/21	0.05396390
rB/USA/09	VCN14/IND/13	0.05413746
rA/USA/09	437/RUS/15	0.05446026
rA/USA/09	593/RUS/15	0.05446132
rA/USA/09	590/RUS/15	0.05446132
rA/USA/09	VCN14/IND/13	0.05497172
rB/USA/09	IM/USA/67	0.05508038
rA/USA/09	IM/USA/67	0.05591464
rB/USA/09	PK33/PAK/17	0.05737206
rB/USA/09	150127/0.2/ALG/15	0.05760873
rB/USA/09	PK2/PAK/17	0.05784275
rB/USA/09	716/RUS/15	0.05808246
rA/USA/09	PK33/PAK/17	0.05820632

rB/USA/09	PK28/PAK/17	0.05822004
rB/USA/09	IBS536/MLS/17	0.05823572
rB/USA/09	GXB02/CHN/20	0.05842466
rA/USA/09	150127/0.2/ALG/15	0.05844299
rB/USA/09	PK19/PAK/17	0.05866349
rB/USA/09	PK12/PAK/17	0.05866452
rA/USA/09	PK2/PAK/17	0.05867701
rA/USA/09	716/RUS/15	0.05891672
rB/USA/09	AC-11727/01/CZE/19	0.05893462
rA/USA/09	PK28/PAK/17	0.05905430
rA/USA/09	IBS536/MLS/17	0.05906998
rA/USA/09	GXB02/CHN/20	0.05925892
rB/USA/09	PK47/PAK/17	0.05948177
rA/USA/09	PK19/PAK/17	0.05949775
rA/USA/09	PK12/PAK/17	0.05949878
rB/USA/09	641/RUS/15	0.05957548
rB/USA/09	636/RUS/15	0.05957653
rB/USA/09	624/RUS/15	0.05957653
rB/USA/09	630/RUS/15	0.05957968
rA/USA/09	AC-11727/01/CZE/19	0.05976888
rA/USA/09	PK47/PAK/17	0.06031603
rA/USA/09	641/RUS/15	0.06040974
rA/USA/09	636/RUS/15	0.06041079
rA/USA/09	624/RUS/15	0.06041079
rA/USA/09	630/RUS/15	0.06041394
rB/USA/09	717/RUS/15	0.06107571
rB/USA/09	F52/70/UK/70	0.06130507
rA/USA/09	717/RUS/15	0.06190997
rA/USA/09	F52/70/UK/70	0.06213933
rB/USA/09	Cu-1wt/GER/75	0.06325230
rA/USA/09	Cu-1wt/GER/75	0.06408656
rB/USA/09	AC-31312/10/RUS/19	0.06431237
rB/USA/09	P2/GER/77	0.06440629
rB/USA/09	903/78/HNG/78	0.06440713
rB/USA/09	D78/NLD/78	0.06444013
rB/USA/09	varE/USA/85	0.06454371
rB/USA/09	GLS/USA/87	0.06480432
rB/USA/09	IBDV-PSNgp/IND/14	0.06514260
rA/USA/09	AC-31312/10/RUS/19	0.06514663
rB/USA/09	HBDY-1/CHN/14	0.06523473
rA/USA/09	P2/GER/77	0.06524055
rA/USA/09	903/78/HNG/78	0.06524139
rA/USA/09	D78/NLD/78	0.06527439
rA/USA/09	varE/USA/85	0.06537797
rA/USA/09	GLS/USA/87	0.06563858
rA/USA/09	IBDV-PSNgp/IND/14	0.06597686
rA/USA/09	HBDY-1/CHN/14	0.06606899
rB/USA/09	HeN20-7103/CHN/20	0.06608692
rB/USA/09	Cu-1/GER/75	0.06689610
rB/USA/09	ZJ2000/CHN/00	0.06690747
rA/USA/09	HeN20-7103/CHN/20	0.06692118
rA/USA/09	Cu-1/GER/75	0.06773036
rA/USA/09	ZJ2000/CHN/00	0.06774173
rB/USA/09	a799/BEL/21	0.06788854
rA/USA/09	a799/BEL/21	0.06872280
rB/USA/09	TL2004/CHN/04	0.06940679
rB/USA/09	IBD17JL01/CHN/19	0.06980854

rB/USA/09	160019/EGY/15	0.06980854
rA/USA/09	TL2004/CHN/04	0.07024105
rA/USA/09	IBD17JL01/CHN/19	0.07064280
rA/USA/09	160019/EGY/15	0.07064280
rB/USA/09	AC-26757/01/RUS/19	0.07200857
rA/USA/09	AC-26757/01/RUS/19	0.07284283
rB/USA/09	Lukert/USA/73	0.07910655
rA/USA/09	Lukert/USA/73	0.07994081
rB/USA/09	dIBDV/2202/URY/14	0.08315060
rB/USA/09	2701/12/URY/12	0.08328121
rA/USA/09	dIBDV/2202/URY/14	0.08398486
rA/USA/09	2701/12/URY/12	0.08411547
rB/USA/09	1302/16/URY/16	0.08707541
rA/USA/09	1302/16/URY/16	0.08790967
rB/USA/09	19P009381/FRA/19	0.09016158
rB/USA/09	221201/URY/12	0.09039503
rA/USA/09	19P009381/FRA/19	0.09099584
rA/USA/09	221201/URY/12	0.09122929
rB/USA/09	P33/15/ARG/15	0.09396951
rA/USA/09	P33/15/ARG/15	0.09480377
rB/USA/09	YL160304//CHN/16	0.09903477
rB/USA/09	1829/ITA/11	0.09955796
rA/USA/09	YL160304//CHN/16	0.09986903
rA/USA/09	1829/ITA/11	0.10039222
rB/USA/09	SHG358/CHN/18	0.11115797
rB/USA/09	IBD16HeN01/CHN/16	0.11118143
rA/USA/09	SHG358/CHN/18	0.11199223
rA/USA/09	IBD16HeN01/CHN/16	0.11201569
rB/USA/09	7/PRT/21	0.11260163
rA/USA/09	7/PRT/21	0.11343589
rB/USA/09	189/PRT/21	0.11466613
rA/USA/09	189/PRT/21	0.11550039
rB/USA/09	SHG19/CHN/19	0.11631205
rB/USA/09	76/PRT/21	0.11668410
rB/USA/09	168/PRT/21	0.11668888
rB/USA/09	UPM1432/MLS/19	0.11687587
rA/USA/09	SHG19/CHN/19	0.11714631
rA/USA/09	76/PRT/21	0.11751836
rA/USA/09	168/PRT/21	0.11752314
rA/USA/09	UPM1432/MLS/19	0.11771013
rB/USA/09	HB-2020-1/CHN/20	0.11868931
rA/USA/09	HB-2020-1/CHN/20	0.11952357
rB/USA/09	QZ191002/CHN/19	0.12036620
rB/USA/09	201/PRT/21	0.12080569
rA/USA/09	QZ191002/CHN/19	0.12120046
rB/USA/09	SHG352/CHN/18	0.12149726
rA/USA/09	201/PRT/21	0.12163995
rB/USA/09	JS19-14701/CHN/19	0.12210932
rA/USA/09	SHG352/CHN/18	0.12233152
rB/USA/09	LN-2020-1/CHN/20	0.12261974
rA/USA/09	JS19-14701/CHN/19	0.12294358
rB/USA/09	FJ2021/CHN/21	0.12307487
rA/USA/09	LN-2020-1/CHN/20	0.12345400
rA/USA/09	FJ2021/CHN/21	0.12390913
rB/USA/09	LN-2020-2/CHN/20	0.12737745
rA/USA/09	LN-2020-2/CHN/20	0.12821171
rB/USA/09	ZD-2018-1/CHN/18	0.13130477

rA/USA/09	ZD-2018-1/CHN/18	0.13213903
rB/USA/09	002-73/AUS/73	0.14457663
rA/USA/09	002-73/AUS/73	0.14541089
rB/USA/09	N1/AUS/99	0.15769261
rA/USA/09	N1/AUS/99	0.15852687
rB/USA/09	23/82/UK/85	0.57582985
rA/USA/09	23/82/UK/85	0.57666411
rB/USA/09	OH/USA/81	0.58250235
rA/USA/09	OH/USA/81	0.58333661
rB/USA/09	PA/00924/14/USA/15	0.58513160
rA/USA/09	PA/00924/14/USA/15	0.58596586

The isolate showing the closest evolutionary distance to rA or rB, is shown in bold red. The evolutionary distance between the study isolates is displayed in bold black.

Table S6. Best fitting model selection for truncated VP2 using Maximum Likelihood fits of 24 different nucleotide substitution models.

Model	BIC	AICc	lnL	I	G	R	FreqA	FreqT	FreqC	FreqG	A=>T	A=>C	A=>G	T=>A	T=>C	T=>G	C=>A	C=>T	C=>G	G=>A	G=>T	G=>C
**GTR+G+I***	**28923.45**	25283.71	-12282.16	0.3973	0.5508	3.54	0.2734	0.2080	0.2679	0.2506	0.03	0.04	0.14	0.05	0.28	0.02	0.04	0.22	0.01	0.15	0.02	0.01
GTR+G	28963.15	25333.54	-12308.08	n/a	0.2481	3.51	0.2734	0.2080	0.2679	0.2506	0.04	0.04	0.14	0.05	0.28	0.02	0.04	0.22	0.01	0.15	0.02	0.01
TN93+G+I	29011.89	25402.55	-12344.60	0.3964	0.5403	3.53	0.2734	0.2080	0.2679	0.2506	0.02	0.03	0.14	0.03	0.28	0.03	0.03	0.22	0.03	0.15	0.02	0.03
K2+G+I	29048.08	25479.28	-12386.98	0.4013	0.5354	3.44	0.25	0.25	0.25	0.25	0.03	0.03	0.19	0.03	0.19	0.03	0.03	0.19	0.03	0.19	0.03	0.03
TN93+G	29054.40	25455.19	-12371.92	n/a	0.2472	3.51	0.2734	0.2080	0.2679	0.2506	0.02	0.03	0.14	0.03	0.28	0.03	0.03	0.22	0.03	0.15	0.02	0.03
HKY+G+I	29071.64	25472.44	-12380.54	0.4055	0.5401	3.53	0.2734	0.2080	0.2679	0.2506	0.02	0.03	0.2	0.03	0.21	0.03	0.03	0.16	0.03	0.21	0.02	0.03
T92+G+I	29073.99	25495.05	-12393.86	0.4024	0.5391	3.43	0.2407	0.2407	0.2593	0.2593	0.03	0.03	0.2	0.03	0.2	0.03	0.03	0.19	0.03	0.19	0.03	0.03
K2+G	29094.98	25536.31	-12416.50	n/a	0.2451	3.43	0.25	0.25	0.25	0.25	0.03	0.03	0.19	0.03	0.19	0.03	0.03	0.19	0.03	0.19	0.03	0.03
HKY+G	29116.45	25527.38	-12409.02	n/a	0.2413	3.52	0.2734	0.2080	0.2679	0.2506	0.02	0.03	0.2	0.03	0.21	0.03	0.03	0.16	0.03	0.21	0.02	0.03
T92+G	29119.47	25550.67	-12422.67	n/a	0.2448	3.42	0.2407	0.2407	0.2593	0.2593	0.03	0.03	0.2	0.03	0.2	0.03	0.03	0.19	0.03	0.19	0.03	0.03
GTR+I	29566.50	25936.89	-12609.76	0.5896	n/a	3.29	0.2734	0.2080	0.2679	0.2506	0.04	0.04	0.14	0.05	0.27	0.03	0.04	0.21	0.01	0.15	0.02	0.01
TN93+I	29659.32	26060.11	-12674.38	0.5903	n/a	3.25	0.2734	0.2080	0.2679	0.2506	0.02	0.03	0.14	0.03	0.27	0.03	0.03	0.21	0.03	0.15	0.02	0.03
K2+I	29715.44	26156.77	-12726.73	0.5912	n/a	3.19	0.25	0.25	0.25	0.25	0.03	0.03	0.19	0.03	0.19	0.03	0.03	0.19	0.03	0.19	0.03	0.03
T92+I	29739.05	26170.25	-12732.46	0.5912	n/a	3.19	0.2407	0.2407	0.2593	0.2593	0.03	0.03	0.2	0.03	0.2	0.03	0.03	0.18	0.03	0.18	0.03	0.03
HKY+I	29744.14	26155.07	-12722.86	0.5929	n/a	3.24	0.2734	0.2080	0.2679	0.2506	0.02	0.03	0.19	0.03	0.2	0.03	0.03	0.16	0.03	0.21	0.02	0.03
JC+G+I	30508.06	26949.39	-13123.04	0.4016	0.5693	0.5	0.25	0.25	0.25	0.25	0.08	0.08	0.08	0.08	0.08	0.08	0.08	0.08	0.08	0.08	0.08	0.08
JC+G	30556.11	27007.57	-13153.13	n/a	0.2592	0.5	0.25	0.25	0.25	0.25	0.08	0.08	0.08	0.08	0.08	0.08	0.08	0.08	0.08	0.08	0.08	0.08
JC+I	31129.68	27581.15	-13439.92	0.5921	n/a	0.5	0.25	0.25	0.25	0.25	0.08	0.08	0.08	0.08	0.08	0.08	0.08	0.08	0.08	0.08	0.08	0.08
GTR	31555.52	27936.04	-13610.34	n/a	n/a	2.35	0.2734	0.2080	0.2679	0.2506	0.04	0.05	0.14	0.05	0.23	0.02	0.05	0.18	0.03	0.15	0.02	0.03
TN93	31581.39	27992.32	-13641.49	n/a	n/a	2.94	0.2734	0.2080	0.2679	0.2506	0.03	0.03	0.14	0.03	0.26	0.03	0.03	0.2	0.03	0.15	0.03	0.03
K2	31606.49	28057.96	-13678.32	n/a	n/a	2.93	0.25	0.25	0.25	0.25	0.03	0.03	0.19	0.03	0.19	0.03	0.03	0.19	0.03	0.19	0.03	0.03
T92	31627.48	28068.82	-13682.75	n/a	n/a	2.93	0.2407	0.2407	0.2593	0.2593	0.03	0.03	0.19	0.03	0.19	0.03	0.03	0.18	0.03	0.18	0.03	0.03
HKY	31667.30	28088.37	-13690.52	n/a	n/a	2.93	0.2734	0.2080	0.2679	0.2506	0.03	0.03	0.19	0.03	0.2	0.03	0.03	0.16	0.03	0.2	0.03	0.03
JC	32965.84	29427.44	-14364.07	n/a	n/a	0.5	0.25	0.25	0.25	0.25	0.08	0.08	0.08	0.08	0.08	0.08	0.08	0.08	0.08	0.08	0.08	0.08

*Model with the lowest BIC scores (Bayesian Information Criterion) is considered to best describe the substitution pattern (shown in bold red). For each model. A total of 1359 positions existed in the final dataset. Abbreviations: K2: Kimura 2-parameter; TR: General Time Reversible; HKY: Hasegawa-Kishino-Yano; TN93: Tamura-Nei; T92: Tamura 3-parameter; JC: Jukes-Cantor.

Table S7. Best fitting model selection for VP1 using Maximum Likelihood fits of 24 different nucleotide substitution models.

Model	BIC	AICc	lnL	I	G	R	FreqA	FreqT	FreqC	FreqG	A=>T	A=>C	A=>G	T=>A	T=>C	T=>G	C=>A	C=>T	C=>G	G=>A	G=>T	G=>C
**GTR+G+I***	**51297.41**	47510.32	-23399.76	0.367	0.421	3.695	0.286	0.193	0.268	0.253	0.03	0.03	0.15	0.05	0.27	0.02	0.03	0.2	0.02	0.17	0.01	0.02
GTR+G	51337.91	47561.48	-23426.35	n/a	0.200	3.586	0.286	0.193	0.268	0.253	0.03	0.03	0.15	0.05	0.27	0.02	0.03	0.19	0.02	0.17	0.01	0.02
TN93+G+I	51378.22	47623.12	-23459.17	0.428	0.516	4.352	0.286	0.193	0.268	0.253	0.02	0.02	0.14	0.03	0.3	0.02	0.03	0.22	0.02	0.16	0.02	0.02
TN93+G	51401.70	47657.27	-23477.25	n/a	0.200	4.295	0.286	0.193	0.268	0.253	0.02	0.02	0.15	0.03	0.29	0.02	0.03	0.21	0.02	0.17	0.02	0.02
K2+G+I	51510.42	47797.98	-23550.61	0.417	0.488	4.226	0.25	0.25	0.25	0.25	0.02	0.02	0.2	0.02	0.2	0.02	0.02	0.2	0.02	0.2	0.02	0.02
HKY+G+I	51523.14	47778.72	-23537.97	0.439	0.521	4.455	0.286	0.193	0.268	0.253	0.02	0.02	0.21	0.03	0.22	0.02	0.03	0.16	0.02	0.23	0.02	0.02
T92+G+I	51542.40	47819.30	-23560.27	0.409	0.475	4.235	0.239	0.239	0.261	0.261	0.02	0.02	0.21	0.02	0.21	0.02	0.02	0.19	0.02	0.19	0.02	0.02
K2+G	51542.60	47840.84	-23573.04	n/a	0.200	4.187	0.25	0.25	0.25	0.25	0.02	0.02	0.2	0.02	0.2	0.02	0.02	0.2	0.02	0.2	0.02	0.02
HKY+G	51549.76	47816.00	-23557.61	n/a	0.195	4.399	0.286	0.193	0.268	0.253	0.02	0.02	0.21	0.03	0.22	0.02	0.03	0.16	0.02	0.23	0.02	0.02
T92+G	51567.24	47854.81	-23579.02	n/a	0.199	4.199	0.239	0.239	0.261	0.261	0.02	0.03	0.21	0.02	0.21	0.03	0.02	0.19	0.03	0.19	0.02	0.03
GTR+I	52411.25	48634.82	-23963.01	0.632	n/a	3.358	0.286	0.193	0.268	0.253	0.03	0.03	0.16	0.05	0.25	0.02	0.03	0.18	0.02	0.18	0.02	0.02
TN93+I	52456.35	48711.92	-24004.57	0.630	n/a	4.035	0.286	0.193	0.268	0.253	0.02	0.03	0.16	0.03	0.27	0.02	0.03	0.2	0.02	0.18	0.02	0.03
K2+I	52562.33	48860.56	-24082.90	0.633	n/a	3.979	0.25	0.25	0.25	0.25	0.03	0.03	0.2	0.03	0.2	0.03	0.03	0.2	0.03	0.2	0.03	0.03
T92+I	52597.08	48884.65	-24093.94	0.632	n/a	3.985	0.239	0.239	0.261	0.261	0.02	0.03	0.21	0.02	0.21	0.03	0.02	0.19	0.03	0.19	0.02	0.03
HKY+I	52609.95	48876.19	-24087.71	0.633	n/a	4.114	0.286	0.193	0.268	0.253	0.02	0.03	0.2	0.03	0.22	0.02	0.03	0.16	0.02	0.23	0.02	0.03
JC+G+I	54995.22	51293.45	-25299.35	0.370	0.437	0.5	0.25	0.25	0.25	0.25	0.08	0.08	0.08	0.08	0.08	0.08	0.08	0.08	0.08	0.08	0.08	0.08
JC+G	55011.40	51320.30	-25313.77	n/a	0.205	0.5	0.25	0.25	0.25	0.25	0.08	0.08	0.08	0.08	0.08	0.08	0.08	0.08	0.08	0.08	0.08	0.08
JC+I	55964.45	52273.35	-25790.30	0.632	n/a	0.5	0.25	0.25	0.25	0.25	0.08	0.08	0.08	0.08	0.08	0.08	0.08	0.08	0.08	0.08	0.08	0.08
GTR	57125.57	53359.81	-26326.51	n/a	n/a	3.082	0.286	0.193	0.268	0.253	0.03	0.04	0.14	0.05	0.27	0.02	0.04	0.19	0.02	0.16	0.01	0.03
TN93	57179.86	53446.10	-26372.66	n/a	n/a	3.771	0.286	0.193	0.268	0.253	0.02	0.03	0.14	0.03	0.29	0.03	0.03	0.21	0.03	0.16	0.02	0.03
K2	57310.05	53618.95	-26463.10	n/a	n/a	3.754	0.25	0.25	0.25	0.25	0.03	0.03	0.2	0.03	0.2	0.03	0.03	0.2	0.03	0.2	0.03	0.03
T92	57349.06	53647.29	-26476.27	n/a	n/a	3.756	0.239	0.239	0.261	0.261	0.03	0.03	0.21	0.03	0.21	0.03	0.03	0.19	0.03	0.19	0.03	0.03
HKY	57477.23	53754.13	-26527.68	n/a	n/a	3.759	0.286	0.193	0.268	0.253	0.02	0.03	0.2	0.03	0.21	0.03	0.03	0.15	0.03	0.23	0.02	0.03
JC	60622.60	56942.16	-28125.71	n/a	n/a	0.5	0.25	0.25	0.25	0.25	0.08	0.08	0.08	0.08	0.08	0.08	0.08	0.08	0.08	0.08	0.08	0.08

*Model with the lowest BIC scores (Bayesian Information Criterion) is to best describing the substitution pattern (shown in bold red). For each model. A total of 2640 positions existed in the final dataset. Abbreviations: K2: Kimura 2-parameter; TR: General Time Reversible; HKY: Hasegawa-Kishino-Yano; TN93: Tamura-Nei; T92: Tamura 3-parameter; JC: Jukes-Cantor.

Table S8. Accession numbers of sequences used for reassortment analysis of Californian isolates.

#	Accession No. (VP1)	Accession No. (VP2)	Isolate	Country	Collection year	Abbreviated name
1	AY029165	AY029166	Irwin Moulthrop (IM)	USA	1967	IM/USA/67
2	HG974566	HG974565	Faragher 52/70	UK	1970	F52/70/UK/70
3	AY918947	AY918948	Lukert	USA	1973	Lukert/USA/73
4	M19336	X03993	002-73	Australia	1973	002-73/AUS/73
5	AF362748	AF362747	Cu-1wt	Germany	1975	Cu-1wt/GER/75
6	X84035	X84034	P2	Germany	1977	P2/GER/77
7	JQ411013	JQ411012	903/78	Hungary	1978	903/78/HNG/78
8	AF499930	AF499929	D78	Netherlands	1978	D78/NLD/78
9	U30819	U30818	OH	USA	1981	OH/USA/81
10	AF362774	AF362773	23/82	UK	1985	23/82/UK/85
11	AF133905	AF133904	variant E	USA	1985	varE/USA/85
12	AY368654	AY368653	GLS	USA	1987	GLS/USA/87
13	HG974564	HG974563	89163	France	1989	89163/FRA/89
14	AF240687	AF240686	D6948	Netherlands	1989	D6948/NLD/89
15	DQ927043	DQ927042	ks	Israel	1990	ks/ISL/90
16	D49707	D49706	OKYM	Japan	1991	OKYM/JPN/91
17	AM167551	AM167550	94432	France	1994	94432/FRA/94
18	AF322445	AF322444	TASIK	Indonesia	1994	TASIK/IDS/94
19	NC_004179	NC_004178	UK661	UK	1994	UK661/UK/94
20	AY705393	AY444873	Gx	China	1996	Gx/CHN/96
21	AF362770	AF362776	BD_3/99	Bangladesh	1999	BD_3/99/BNG/99
22	DQ166818	AF321056	ZJ2000	China	2000	ZJ2000/CHN/00
23	AB368971	AB368970	KMRG-48	Tanzania	2002	KMRG-48/TNZ/02
24	AJ880090	AJ879932	02015.1	Venezuela	2002	02015.1/VNZ/02
25	MT629833	MT629830	Bug/03	Poland	2003	Bug/03/POL/03
26	DQ118374	DQ088175	TL2004	China	2004	TL2004/CHN/04
27	GQ451331	GQ451330	HLJ-0504	China	2005	HLJ-0504/CHN/05
28	JF907704	JF907703	CAH495	USA	2009	CAH495/USA/09
29	JF907705	JF907702	CAHFS-785	USA	2009	CAHFS-785/USA/09
30	MT629835	MT629832	75/11	Poland	2011	75/11/POL/11
31	KX520665	KY930929	1829	Italy	2011	1829/ITA/11
32	OP225971	OP225970	25/11	Latvia	2011	25/11/LTV/11
33	MN313619	MN313613	221201	Uruguay	2012	221201/URY/12
34	MN313620	MN313614	2701/12	Uruguay	2012	2701/12/URY/12
35	MN485881	MN485880	SD-2013-1	China	2013	SD-2013-1/CHN/13
36	KU578101	KU578100	VCN14	India	2013	VCN14/IND/13
37	KP676468	KP676467	IBD13HeB01	China	2013	IBD13HeB01/CHN/13
38	MT629834	MT629831	117/14	Poland	2014	117/14/POL/14
39	MK501732	MK501731	IBDV-PSNgp	India	2014	IBDV-PSNgp/IND/14
40	MG739299	MG739298	li4129	Finland	2014	li4129/FIN/14
41	MF969112	MF969111	150133/3.2	Algeria	2014	150133/3.2/ALG/14

42	KX592159	KX592158	HBDY-1	China	2014	HBDY-1/CHN/14
43	KU578103	KU578102	RPM14	India	2014	RPM14/IND/14
44	KU578105	KU578104	THI14	India	2014	THI14/IND/14
45	KU558698	KU558697	EDE14	India	2014	EDE14/IND/14
46	KU578099	KU578098	NKL14	India	2014	NKL14/IND/14
47	KT884453	KT884452	BGE14	India	2014	BGE14/IND/15
48	KT336458	KT336459	dIBDV/UY/2014/2202	Uruguay	2014	dIBDV/2202/URY/14
49	MN313616	MN313610	P33/15	Argentina	2015	P33/15/ARG/15
50	KY597860	KY610530	160021	Egypt	2015	160021/EGY/15
51	KY597861	KY610531	160023	Egypt	2015	160023/EGY/15
52	KY597863	KY610529	160019	Egypt	2015	160019/EGY/15
53	MF969106	MF969105	150124/1.1	Algeria	2016	150124/1.1/ALG/16
54	MF969108	MF969107	150127/0.2	Algeria	2015	150127/0.2/ALG/15
55	MF969110	MF969109	150128/4.1	Algeria	2015	150128/4.1/ALG/15
56	MF969114	MF969113	150136/1.1	Algeria	2015	150136/1.1/ALG/15
57	MF969116	MF969115	150144/5.1	Algeria	2015	150144/5.1/ALG/15
58	-	KP642112	IBDV/Turkey/PA/00924/14	USA	2015	PA/00924/14/USA/15
59	KT870149	KT870148	BGE15	India	2015	BGE15/IND/15
60	MZ066616	MZ066614	YL160304	China	2016	YL160304//CHN/16
61	MT179711	MT179710	IBD16HeN01	China	2016	IBD16HeN01/CHN/16
62	MN313621	MN313615	1/chicken/URY/1302/16	Uruguay	2016	1302/16/URY/16
63	MH644847	MH644846	DD1	Russia	2016	DD1/RUS/16
64	MT505344	MT505339	IBS536	Malaysia	2017	IBS536/MLS/17
65	MT505345	MT505340	IBS624	Malaysia	2017	IBS624/MLS/17
66	MN786769	MN786767	D3976/1	Germany	2017	D3976/1/GER/17
67	MK919703	MK800149	PK12	Pakistan	2017	PK12/PAK/17
68	MK919704	MK800150	PK19	Pakistan	2017	PK19/PAK/17
69	MK919705	MK800151	PK28	Pakistan	2017	PK28/PAK/17
70	MK919706	MK800152	PK33	Pakistan	2017	PK33/PAK/17
71	MK919707	MK800153	PK47	Pakistan	2017	PK47/PAK/17
72	MK580166	MK580161	61-7	Morocco	2018	61-7/MOR/18
73	MF996500	MF996499	PK2	Pakistan	2017	PK2/PAK/17
74	-	MZ614612	THCU07	Thailand	2017	THCU07/TAI/17
75	MT505346	MT505341	UPM766	Malaysia	2018	UPM766/MLS/18
76	MT505347	MT505342	UPM1056	Malaysia	2018	UPM1056/MLS/18
77	MN786770	MN786768	D4320/6	Denmark	2018	D4320/6/DEN/18
78	MT446362	MW030633	LJ-5	China	2018	LJ-5/CHN/18
79	MT179723	MT179721	SHG358	China	2018	SHG358/CHN/18
80	MT179722	MT179720	SHG352	China	2018	SHG352/CHN/18
81	MN485883	MN485882	ZD-2018-1	China	2018	ZD-2018-1/CHN/18
82	MK580164	MK580160	9-1	Morocco	2017	9-1/MOR/17
83	MK580165	MK580162	8	Morocco	2017	8/MOR/17
84	MK580167	MK580163	64	Morocco	2018	64/MOR/18
85	MK472712	MK472711	S18	China	2018	S18/CHN/18

86	OK043827	OK043826	19P009381	France	2019	19P009381/FRA/19
87	MZ066615	MZ066613	QZ191002	China	2019	QZ191002/CHN/19
88	MW700333	MW700332	JS19-14701	China	2019	JS19-14701/CHN/19
89	MT505348	MT505343	UPM1432	Malaysia	2019	UPM1432/MLS/19
90	MT066170	MT066169	SH19	China	2019	SH19/CHN/19
91	MN604242	MN604241	IBD17JL01	China	2019	IBD17JL01/CHN/19
92	MN393077	MN393076	SHG19	China	2019	SHG19/CHN/19
93	MZ740265	MZ740264	GXB02	China	2020	GXB02/CHN/20
94	MW682878	MW682877	HeN20-7103	China	2020	HeN20-7103/CHN/20
95	MZ367374	MZ367373	4439001	Belgium	2020	4439001/BEL/20
96	MW767378	MW767374	LN-2020-1	China	2020	LN-2020-1/CHN/20
97	MW767379	MW767375	LN-2020-2	China	2020	LN-2020-2/CHN/20
98	MW767380	MW767376	SD-2020-1	China	2020	SD-2020-1/CHN/20
99	MW767377	MW767998	HB-2020-1	China	2020	HB-2020-1/CHN/20
100	MZ593903	MZ593902	FJ2021	China	2021	FJ2021/CHN/21
101	DQ927041	DQ927040	mb	Israel	2008	mb/ISL/08
102	AF362775	D00867	Cu-1	Germany	1975	Cu-1/GER/75
103	KX759557	KX759603	83	Poland	2011	83/POL/11
104	KX759558	KX759604	98	Poland	2013	98/POL/13
105	KX759559	KX759610	217	Poland	2013	217/POL/13
106	KX759560	KX759605	115/14A	Poland	2014	115/14A/POL/14
107	KX759561	KX759606	115/14B	Poland	2014	115/14B/POL/14
108	KX759563	KX759608	131	Poland	2014	131/POL/14
109	KX759564	KX759609	170W	Poland	2014	170W/POL/14
110	KX759550	KX759596	176	Poland	2014	176/POL/14
111	KX759565	KX759611	189	Poland	2014	189/POL/14
112	KX759566	KX759612	216	Poland	2014	216/POL/14
113	KX759551	KX759597	236	Poland	2014	236/POL/14
114	KX759552	KX759598	20	Poland	2015	20/POL/15
115	KX759553	KX759599	72	Poland	2015	72/POL/15
116	KX759567	KX759613	96	Poland	2015	96/POL/15
117	KX759568	KX759614	123	Poland	2015	123/POL/15
118	KX759569	KX759615	200	Poland	2015	200/POL/15
119	ON152535	ON152424	a799	Belgium	2021	a799/BEL/21
120	ON152537	ON152426	a664	Denmark	2021	a664/DEN/21
121	ON152586	ON152475	b335	Netherlands	2021	b335/NLD/21
122	ON152592	ON152481	a833	Portugal	2021	a833/PRT/21
123	ON152570	ON152459	a893	Germany	2021	a893/GER/21
124	ON152573	ON152462	b507	Germany	2021	b507/GER/21
125	ON152538	ON152427	a749	France	2021	a749/FRA/21
126	ON152551	ON152440	b292	France	2021	b292/FRA/21
127	ON152556	ON152445	b317	France	2021	b317/FRA/21
128	ON152562	ON152451	b524	France	2021	b524/FRA/21
129	ON152564	ON152453	b542	France	2021	b542/FRA/21

130	ON152608	ON152497	a227	UK	2021	a227/UK/21
131	ON152619	ON152508	a452	UK	2021	a452/UK/21
132	ON152630	ON152519	a524	UK	2021	a524/UK/21
133	ON152638	ON152527	b289	UK	2021	b289/UK/21
134	ON152642	ON152531	b630	UK	2021	b630/UK/21
135	ON152575	-	1/chicken/ITA/a056/21	Italy	2021	a059/ITA/21
136	ON152577	-	1/chicken/ITA/a186/21	Italy	2021	a186/ITA/21
137	ON152576	-	1/chicken/ITA/a163/21	Italy	2021	a163/ITA/21
138	ON152578	-	1/chicken/ITA/a191/21	Italy	2021	a191/ITA/21
139	ON152579	-	1/chicken/ITA/a217/21	Italy	2021	a217/ITA/21
140	ON152580	-	1/chicken/ITA/a393/21	Italy	2021	a393/ITA/21
141	MT050426	MT050435	AC-09389/02	Irleand	2019	AC-09389/02/IRL/19
142	MT050427	MT050436	AC-11727/01	Czech Republic	2019	AC-11727/01/CZE/19
143	MT050431	MT050440	AC-26757/01	Russia	2019	AC-26757/01/RUS/19
144	MT050433	MT050442	AC-31312/10	Russia	2019	AC-31312/10/RUS/19
145	AJ878641	AF381005	N1	Austraila	1999	N1/AUS/99
146	AB368956	AB200975	KMRG-00	Tanzania	2001	KMRG-00/TNZ/01
147	AB368955	AB200976	KDSM-02	Tanzania	2004	KDSM-02/TNZ/04
148	MF142463	MF142509	189	Russia	2014	189/RUS/14
149	MF142464	MF142514	224	Russia	2014	224/RUS/14
150	MF142465	MF142515	232	Russia	2015	232/RUS/15
151	MF142466	MF142517	276	Jordan	2014	276/JOR/14
152	MF142473	MF142537	437	Russia	2015	437/RUS/15
153	MF142478	MF142549	590	Russia	2015	590/RUS/15
154	MF142479	MF142550	593	Russia	2015	593/RUS/15
155	MF142481	MF142552	624	Russia	2015	624/RUS/15
156	MF142482	MF142554	630	Russia	2015	630/RUS/15
157	MF142483	MF142555	636	Russia	2015	636/RUS/15
158	MF142484	MF142556	641	Russia	2015	641/RUS/15
159	MF142486	MF142561	711	Russia	2015	711/RUS/15
160	MF142487	MF142562	713	Russia	2015	713/RUS/15
161	MF142488	MF142563	716	Russia	2015	716/RUS/15
162	MF142489	MF142564	717	Russia	2015	717/RUS/15
163	MF142490	MF142565	727	Russia	2015	727/RUS/15
164	MF142491	MF142566	731	Russia	2015	731/RUS/15
165	MF142492	MF142567	735	Russia	2015	735/RUS/15
166	MF142497	MF142578	774	Kazakhstan	2016	774/KAZ/16
167	MN765057	MN737273	UVC1	Nigeria	2019	UVC1/NG/19
168	MN765058	MN737277	AT6	Nigeria	2019	AT6/NG/19
169	KP266340	KP266329	BAUCHI37	Nigeria	2012	BAUCHI37/NG/12
170	KP266339	KP152305	PLATEAU7	Nigeria	2009	PLATEAU7/NG/09
171	MZ687403	MZ687395	7	Portugal	2021	7/PRT/21
172	MZ687405	MZ687397	76	Portugal	2021	76/PRT/21
173	MZ687406	MZ687400	168	Portugal	2021	168/PRT/21

174	MZ687407	MZ687401	189	Portugal	2021	189/PRT/21
175	MZ687408	MZ687402	201	Portugal	2021	201/PRT/21
176	-	MN702848	D4628/2	Czech Republic	2019	D4628/2/CZE/19
177	-	MN702836	D3994/1/8	Netherlands	2017	D3994/1/8/NLD/17
178	-	MN702837	D3996/1/1	Belgium	2017	D3996/1/1/BEL/17
179	-	MN702847	D4389/1	Sweden	2018	D4389/1/SWD/18
180	-	MN702839	D4051/1	Sweden	2017	D4051/1/SWD/17

Table S9. Evolutionary divergence estimates between mature VP1 sequences of the study isolates and other IBDV isolates for gene-wise analysis.

Species 1	Species 2	Distance (*d*)
rA/USA/09	rB/USA/09	0.005073
**rA/USA/09**	**D6948/NLD/89**	**0.016861**
rA/USA/09	DD1/RUS/16	0.018033
rA/USA/09	TASIK/IDS/94	0.018037
rA/USA/09	89163/FRA/89	0.018814
rA/USA/09	ks/ISL/90	0.019203
rA/USA/09	mb/ISL/93	0.020362
**rB/USA/09**	**D6948/NLD/89**	**0.021370**
rA/USA/09	HuB-1/CHN/00	0.022396
rB/USA/09	DD1/RUS/16	0.022542
rB/USA/09	TASIK/IDS/94	0.022546
rB/USA/09	89163/FRA/89	0.023323
rA/USA/09	UK661/UK/95	0.023626
rB/USA/09	ks/ISL/90	0.023711
rA/USA/09	Cro-Ig/CRT/02	0.024389
rB/USA/09	mb/ISL/93	0.024871
rB/USA/09	HuB-1/CHN/00	0.026905
rB/USA/09	UK661/UK/95	0.028134
rB/USA/09	Cro-Ig/CRT/02	0.028898
rA/USA/09	T09/NG/98	0.036101
rB/USA/09	T09/NG/98	0.040610
rA/USA/09	150133/3.2/ALG/14	0.062025
rA/USA/09	TN46/19/TUN/19	0.062046
rA/USA/09	150128/4.1/ALG/15	0.062412
rB/USA/09	150133/3.2/ALG/14	0.066534
rB/USA/09	TN46/19/TUN/19	0.066555
rB/USA/09	150128/4.1/ALG/15	0.066920
rA/USA/09	150124/1.1/ALG/16	0.184470
rA/USA/09	150144/5.1/ALG/15	0.185136
rB/USA/09	150124/1.1/ALG/16	0.188979
rA/USA/09	9109/USA/03	0.189158
rB/USA/09	150144/5.1/ALG/15	0.189645
rA/USA/09	JD1/CHN/97	0.189883
rA/USA/09	HZ2/CHN/97	0.190093
rA/USA/09	STC/USA/67	0.191309
rA/USA/09	GZ/96/CHN/04	0.191761
rB/USA/09	9109/USA/03	0.193667
rA/USA/09	F52/70/UK/70	0.194124
rA/USA/09	903/78/HNG/78	0.194193
rB/USA/09	JD1/CHN/97	0.194392
rB/USA/09	HZ2/CHN/97	0.194602
rA/USA/09	Bpop-03/POL/03	0.194974
rA/USA/09	B87/CHN/06	0.195233
rA/USA/09	CEF94/NLD/73	0.195627
rB/USA/09	STC/USA/67	0.195818
rA/USA/09	KZC-104/ZMB/04	0.196024
rB/USA/09	GZ/96/CHN/04	0.196270
rA/USA/09	Gt/CHN/06	0.196415
rA/USA/09	Cu-1wt/GER/75	0.198315
rB/USA/09	F52/70/UK/70	0.198633
rB/USA/09	903/78/HNG/78	0.198702
rA/USA/09	Harbin-1/CHN/92	0.199013
rB/USA/09	Bpop-03/POL/03	0.199483

rB/USA/09	B87/CHN/06	0.199742
rB/USA/09	CEF94/NLD/73	0.200136
rB/USA/09	KZC-104/ZMB/04	0.200533
rB/USA/09	Gt/CHN/06	0.200923
rA/USA/09	OH/USA/82	0.202114
rB/USA/09	Cu-1wt/GER/75	0.202823
rA/USA/09	VarE/USA/94	0.203259
rB/USA/09	Harbin-1/CHN/92	0.203522
rA/USA/09	dIBDV/UY/2014/2202	0.205680
rB/USA/09	OH/USA/82	0.206623
rB/USA/09	VarE/USA/94	0.207768
rB/USA/09	dIBDV/UY/2014/2202	0.210189

rA and rB, showing the closest evolutionary distance to each other, are displayed in bold red. The isolate in bold black is the next closest to rA or rB.

Table S10. Evolutionary divergence estimates between VP2 sequences of the study isolates and other IBDV isolates for gene-wise analysis.

Species 1	Species 2	Distance (*d*)
**rA/USA/09**	**rB/USA/09**	**0.00551634**
**rB/USA/09**	**CAHFS-785/USA/11**	**0.01211331**
**rA/USA/09**	**CAHFS-785/USA/11**	**0.01289615**
rB/USA/09	D6948/NLD/89	0.01345644
rB/USA/09	SH/92/KOR/92	0.01422350
rB/USA/09	ks/ISL/90	0.01422914
rA/USA/09	D6948/NLD/89	0.01423928
rA/USA/09	SH/92/KOR/92	0.01500634
rB/USA/09	3529/MLS/92	0.01500896
rA/USA/09	ks/ISL/90	0.01501198
rB/USA/09	89163/FRA/89	0.01579157
rA/USA/09	3529/MLS/92	0.01579180
rB/USA/09	IBDV78/ABIC/NG/11	0.01579200
rB/USA/09	mb/ISL/93	0.01579200
rB/USA/09	UK661/UK/95	0.01579597
rB/USA/09	Ventri/IND/10	0.01656166
rA/USA/09	89163/FRA/89	0.01657442
rA/USA/09	IBDV78/ABIC/NG/11	0.01657485
rA/USA/09	mb/ISL/93	0.01657485
rA/USA/09	UK661/UK/95	0.01657882
rB/USA/09	DD1/RUS/16	0.01658214
rB/USA/09	HuB-1/CHN/00	0.01686122
rA/USA/09	Ventri/IND/10	0.01734451
rA/USA/09	DD1/RUS/16	0.01736499
rB/USA/09	GZ/96/CHN/04	0.01737064
rA/USA/09	HuB-1/CHN/00	0.01764406
rB/USA/09	Harbin-1/CHN/92	0.01814449
rA/USA/09	GZ/96/CHN/04	0.01815348
rA/USA/09	Harbin-1/CHN/92	0.01892733
rB/USA/09	HHN81/FRA/19	0.01973027
rB/USA/09	HHN79/FRA/19	0.01973967
rB/USA/09	TASIK/IDS/94	0.01976089
rA/USA/09	HHN81/FRA/19	0.02051312
rA/USA/09	HHN79/FRA/19	0.02052252
rA/USA/09	TASIK/IDS/94	0.02054373
rB/USA/09	Bpop-03/POL/03	0.02130091
rB/USA/09	Chinju/KOR/02	0.02140288
rA/USA/09	Bpop-03/POL/03	0.02208376
rA/USA/09	Chinju/KOR/02	0.02218572
rB/USA/09	7741-SEGA/USA/12	0.02376295
rA/USA/09	7741-SEGA/USA/12	0.02454579
rB/USA/09	Cro-Ig/CRT/02	0.02470776
rA/USA/09	Cro-Ig/CRT/02	0.02549061
rB/USA/09	KZC-104/ZMB/04	0.02811255
rA/USA/09	KZC-104/ZMB/04	0.02889540
rB/USA/09	T09/NG/98	0.02893588
rA/USA/09	T09/NG/98	0.02971873
rB/USA/09	150124/1.1/ALG/16	0.03910104
rA/USA/09	150124/1.1/ALG/16	0.03988389
rB/USA/09	150133/3.2/ALG/14	0.03992714
rB/USA/09	TN46/19/TUN/19	0.04043404
rB/USA/09	150128/4.1/ALG/15	0.04070645
rA/USA/09	150133/3.2/ALG/14	0.04070999

rB/USA/09	150144/5.1/ALG/15	0.04071140
rA/USA/09	TN46/19/TUN/19	0.04121688
rA/USA/09	150128/4.1/ALG/15	0.04148929
rA/USA/09	150144/5.1/ALG/15	0.04149425
rB/USA/09	STC/USA/67	0.05187681
rB/USA/09	F52/70/UK/70	0.05228597
rA/USA/09	STC/USA/67	0.05265965
rA/USA/09	F52/70/UK/70	0.05306881
rB/USA/09	903/78/HNG/78	0.05321085
rB/USA/09	CEF94/NLD/73	0.05399110
rA/USA/09	903/78/HNG/78	0.05399370
rB/USA/09	Cu-1wt/GER/75	0.05401096
rA/USA/09	CEF94/NLD/73	0.05477395
rA/USA/09	Cu-1wt/GER/75	0.05479381
rB/USA/09	JD1/CHN/97	0.05479648
rB/USA/09	Gt/CHN/06	0.05555440
rA/USA/09	JD1/CHN/97	0.05557933
rA/USA/09	Gt/CHN/06	0.05633724
rB/USA/09	HZ2/CHN/97	0.05713022
rA/USA/09	HZ2/CHN/97	0.05791306
rB/USA/09	B87/CHN/06	0.05794985
rA/USA/09	B87/CHN/06	0.05873270
rB/USA/09	VarE/USA/94	0.05948172
rA/USA/09	VarE/USA/94	0.06026457
rB/USA/09	9109/USA/03	0.06987537
rA/USA/09	9109/USA/03	0.07065821
rB/USA/09	dIBDV/2202/URY/14	0.07189605
rA/USA/09	dIBDV/2202/URY/14	0.07267890
rB/USA/09	OH/USA/82	0.48834297
rA/USA/09	OH/USA/82	0.48912581

rA and rB, showing the least evolutionary divergence from each other, are demonstrated in bold red. The isolate in bold black is the next closest to rA or rB.

Table S11. Evolutionary divergence estimates between VP3 sequences of the study isolates and other IBDV isolates for gene-wise analysis.

Species 1	Species 2	Distance (*d*)
**rA/USA/09**	**rB/USA/09**	**0.0057214**
**rB/USA/09**	**ks/ISL/90**	**0.0191541**
rB/USA/09	Ventri/IND/10	0.0191697
rB/USA/09	mb/ISL/93	0.0191697
rB/USA/09	IBDV78/ABIC/NG/11	0.0191697
**rA/USA/09**	**ks/ISL/90**	**0.0221667**
rA/USA/09	Ventri/IND/10	0.0221822
rA/USA/09	mb/ISL/93	0.0221822
rA/USA/09	IBDV78/ABIC/NG/11	0.0221822
rB/USA/09	TASIK/IDS/94	0.0233849
rB/USA/09	3529/MLS/92	0.0247903
rB/USA/09	89163/FRA/89	0.0248012
rB/USA/09	D6948/NLD/89	0.0248055
rB/USA/09	Chinju/KOR/02	0.0248214
rB/USA/09	DD1/RUS/16	0.0261998
rA/USA/09	TASIK/IDS/94	0.0263974
rA/USA/09	3529/MLS/92	0.0278028
rA/USA/09	89163/FRA/89	0.0278137
rA/USA/09	D6948/NLD/89	0.0278180
rA/USA/09	Chinju/KOR/02	0.0278339
rB/USA/09	HHN81/FRA/19	0.0282603
rB/USA/09	HHN79/FRA/19	0.0282603
rB/USA/09	CAHFS-785/USA/11	0.0291500
rB/USA/09	HuB-1/CHN/00	0.0291746
rA/USA/09	DD1/RUS/16	0.0292124
rB/USA/09	SH/92/KOR/92	0.0300000
rB/USA/09	UK661/UK/95	0.0305520
rB/USA/09	GZ/96/CHN/04	0.0306055
rA/USA/09	HHN81/FRA/19	0.0312729
rA/USA/09	HHN79/FRA/19	0.0312729
rB/USA/09	Harbin-1/CHN/92	0.0319937
rB/USA/09	Bpop-03/POL/03	0.0320555
rB/USA/09	T09/NG/98	0.0320716
rA/USA/09	CAHFS-785/USA/11	0.0321626
rA/USA/09	HuB-1/CHN/00	0.0321871
rB/USA/09	Cro-Ig/CRT/02	0.0322795
rA/USA/09	SH/92/KOR/92	0.0330126
rB/USA/09	7741-SEGA/USA/12	0.0335165
rA/USA/09	UK661/UK/95	0.0335646
rA/USA/09	GZ/96/CHN/04	0.0336180
rA/USA/09	Harbin-1/CHN/92	0.0350063
rA/USA/09	Bpop-03/POL/03	0.0350680
rA/USA/09	T09/NG/98	0.0350842
rA/USA/09	Cro-Ig/CRT/02	0.0352921
rA/USA/09	7741-SEGA/USA/12	0.0365290
rB/USA/09	TN46/19/TUN/19	0.0381045
rB/USA/09	150144/5.1/ALG/15	0.0395292
rB/USA/09	150124/1.1/ALG/16	0.0395436
rA/USA/09	TN46/19/TUN/19	0.0411170
rB/USA/09	150133/3.2/ALG/14	0.0415670
rA/USA/09	150144/5.1/ALG/15	0.0425418
rA/USA/09	150124/1.1/ALG/16	0.0425561
rB/USA/09	150128/4.1/ALG/15	0.0429482

rA/USA/09	150133/3.2/ALG/14	0.0445796
rB/USA/09	KZC-104/ZMB/04	0.0450113
rA/USA/09	150128/4.1/ALG/15	0.0459607
rA/USA/09	KZC-104/ZMB/04	0.0480238
rB/USA/09	9109/USA/03	0.0638955
rB/USA/09	CEF94/NLD/73	0.0662091
rB/USA/09	Gt/CHN/06	0.0662169
rB/USA/09	903/78/HNG/78	0.0662246
rA/USA/09	9109/USA/03	0.0669080
rB/USA/09	JD1/CHN/97	0.0691077
rA/USA/09	CEF94/NLD/73	0.0692216
rA/USA/09	Gt/CHN/06	0.0692294
rA/USA/09	903/78/HNG/78	0.0692372
rB/USA/09	B87/CHN/06	0.0705421
rB/USA/09	Cu-1wt/GER/75	0.0706318
rB/USA/09	HZ2/CHN/97	0.0719826
rA/USA/09	JD1/CHN/97	0.0721203
rA/USA/09	B87/CHN/06	0.0735547
rA/USA/09	Cu-1wt/GER/75	0.0736443
rA/USA/09	HZ2/CHN/97	0.0749951
rB/USA/09	STC/USA/67	0.0753763
rB/USA/09	F52/70/UK/70	0.0761823
rB/USA/09	VarE/USA/94	0.0776544
rA/USA/09	STC/USA/67	0.0783888
rA/USA/09	F52/70/UK/70	0.0791949
rA/USA/09	VarE/USA/94	0.0806669
rB/USA/09	dIBDV/2202/URY/14	0.0974264
rA/USA/09	dIBDV/2202/URY/14	0.1004390
rB/USA/09	OH/USA/82	0.3458822
rA/USA/09	OH/USA/82	0.3488948

The study isolates rA and rB, displayed in bold red, were the closest evolutionary related with respect to the VP3 sequences. These isolates were next closely related to the ks isolate shown in bold black.

Table S12. Evolutionary divergence estimates between VP4 sequences of the study isolates and other IBDV isolates for gene-wise analysis.

Species 1	Species 2	Distance (*d*)
**rA/USA/09**	**rB/USA/09**	**0.0084760**
**rB/USA/09**	**ks/ISL/90**	**0.0114482**
rB/USA/09	CAHFS-785/USA/11	0.0128848
rB/USA/09	Ventri/IND/10	0.0142503
rB/USA/09	IBDV78/ABIC/NG/11	0.0142586
rB/USA/09	mb/ISL/93	0.0142586
rB/USA/09	89163/FRA/89	0.0142907
rB/USA/09	TASIK/IDS/94	0.0156744
rB/USA/09	D6948/NLD/89	0.0156770
rB/USA/09	GZ/96/CHN/04	0.0170629
**rA/USA/09**	**ks/ISL/90**	**0.0172084**
rA/USA/09	CAHFS-785/USA/11	0.0186449
rB/USA/09	Bpop-03/POL/03	0.0198821
rB/USA/09	DD1/RUS/16	0.0199034
rA/USA/09	Ventri/IND/10	0.0200105
rA/USA/09	IBDV78/ABIC/NG/11	0.0200187
rA/USA/09	mb/ISL/93	0.0200187
rA/USA/09	89163/FRA/89	0.0200509
rB/USA/09	Harbin-1/CHN/92	0.0212618
rB/USA/09	3529/MLS/92	0.0213119
rB/USA/09	HuB-1/CHN/00	0.0213224
rB/USA/09	SH/92/KOR/92	0.0213647
rA/USA/09	TASIK/IDS/94	0.0214346
rA/USA/09	D6948/NLD/89	0.0214372
rA/USA/09	GZ/96/CHN/04	0.0228231
rB/USA/09	Chinju/KOR/02	0.0242065
rB/USA/09	Cro-Ig/CRT/02	0.0242292
rB/USA/09	UK661/UK/95	0.0256032
rA/USA/09	Bpop-03/POL/03	0.0256423
rA/USA/09	DD1/RUS/16	0.0256636
rA/USA/09	Harbin-1/CHN/92	0.0270220
rA/USA/09	3529/MLS/92	0.0270721
rA/USA/09	HuB-1/CHN/00	0.0270825
rA/USA/09	SH/92/KOR/92	0.0271249
rB/USA/09	HHN81/FRA/19	0.0285076
rB/USA/09	HHN79/FRA/19	0.0285076
rB/USA/09	7741-SEGA/USA/12	0.0286803
rB/USA/09	T09/NG/98	0.0288216
rA/USA/09	Chinju/KOR/02	0.0299667
rA/USA/09	Cro-Ig/CRT/02	0.0299893
rA/USA/09	UK661/UK/95	0.0313634
rA/USA/09	HHN81/FRA/19	0.0342678
rA/USA/09	HHN79/FRA/19	0.0342678
rA/USA/09	7741-SEGA/USA/12	0.0344404
rA/USA/09	T09/NG/98	0.0345817
rB/USA/09	KZC-104/ZMB/04	0.0358359
rA/USA/09	KZC-104/ZMB/04	0.0415961
rB/USA/09	150133/3.2/ALG/14	0.0447740
rB/USA/09	TN46/19/TUN/19	0.0454510
rB/USA/09	150128/4.1/ALG/15	0.0475604
rB/USA/09	150124/1.1/ALG/16	0.0489805
rA/USA/09	150133/3.2/ALG/14	0.0505342
rA/USA/09	TN46/19/TUN/19	0.0512112

rB/USA/09	150144/5.1/ALG/15	0.0519517
rA/USA/09	150128/4.1/ALG/15	0.0533206
rA/USA/09	150124/1.1/ALG/16	0.0547407
rA/USA/09	150144/5.1/ALG/15	0.0577119
rB/USA/09	Cu-1wt/GER/75	0.0657544
rB/USA/09	F52/70/UK/70	0.0713938
rA/USA/09	Cu-1wt/GER/75	0.0715146
rB/USA/09	STC/USA/67	0.0717589
rB/USA/09	VarE/USA/94	0.0725303
rB/USA/09	903/78/HNG/78	0.0729188
rB/USA/09	CEF94/NLD/73	0.0743148
rB/USA/09	Gt/CHN/06	0.0757049
rB/USA/09	JD1/CHN/97	0.0757081
rA/USA/09	F52/70/UK/70	0.0771540
rA/USA/09	STC/USA/67	0.0775191
rA/USA/09	VarE/USA/94	0.0782905
rB/USA/09	HZ2/CHN/97	0.0784997
rA/USA/09	903/78/HNG/78	0.0786789
rB/USA/09	B87/CHN/06	0.0799084
rA/USA/09	CEF94/NLD/73	0.0800750
rA/USA/09	Gt/CHN/06	0.0814651
rA/USA/09	JD1/CHN/97	0.0814683
rA/USA/09	HZ2/CHN/97	0.0842599
rB/USA/09	9109/USA/03	0.0844826
rA/USA/09	B87/CHN/06	0.0856686
rA/USA/09	9109/USA/03	0.0902427
rB/USA/09	dIBDV/2202/URY/14	0.1059649
rA/USA/09	dIBDV/2202/URY/14	0.1117251
rB/USA/09	OH/USA/82	0.3923561
rA/USA/09	OH/USA/82	0.3981163

The study isolates rA and rB, displayed in bold red, were the closest evolutionary related in terms of the VP4 sequences. These isolates were next closely related to the ks isolate shown in bold black.

Table S13. Evolutionary divergence estimates between VP5 sequences of the study isolates and other IBDV isolates for gene-wise analysis.

Species 1	Species 2	Distance (*d*)
**rB/USA/09**	**mb/ISL/93**	**0.0023832**
rB/USA/09	ks/ISL/90	0.0023966
rB/USA/09	IBDV78/ABIC/NG/11	0.0023966
rB/USA/09	HHN79/FRA/19	0.0024367
rB/USA/09	KZC-104/ZMB/04	0.0024501
rB/USA/09	SH/92/KOR/92	0.0024769
rB/USA/09	DD1/RUS/16	0.0047146
rB/USA/09	Chinju/KOR/02	0.0047146
rB/USA/09	HHN81/FRA/19	0.0047661
rB/USA/09	TASIK/IDS/94	0.0047681
rB/USA/09	UK661/UK/95	0.0047794
rB/USA/09	D6948/NLD/89	0.0047800
rB/USA/09	3529/MLS/92	0.0055829
rB/USA/09	Ventri/IND/10	0.0062084
**rA/USA/09**	**rB/USA/09**	**0.0069523**
rB/USA/09	CAHFS-785/USA/11	0.0070465
rB/USA/09	7741-SEGA/USA/12	0.0070465
rB/USA/09	89163/FRA/89	0.0070594
rB/USA/09	T09/NG/98	0.0070670
rB/USA/09	Bpop-03/POL/03	0.0070727
rB/USA/09	Cro-Ig/CRT/02	0.0071158
rA/USA/09	mb/ISL/93	0.0093087
rA/USA/09	ks/ISL/90	0.0093221
rA/USA/09	IBDV78/ABIC/NG/11	0.0093221
rA/USA/09	HHN79/FRA/19	0.0093622
rA/USA/09	KZC-104/ZMB/04	0.0093756
rA/USA/09	SH/92/KOR/92	0.0094024
rB/USA/09	HuB-1/CHN/00	0.0094804
rA/USA/09	DD1/RUS/16	0.0116401
rA/USA/09	Chinju/KOR/02	0.0116401
rA/USA/09	HHN81/FRA/19	0.0116916
rA/USA/09	TASIK/IDS/94	0.0116936
rA/USA/09	UK661/UK/95	0.0117049
rA/USA/09	D6948/NLD/89	0.0117055
rB/USA/09	Harbin-1/CHN/92	0.0117496
rB/USA/09	GZ/96/CHN/04	0.0117630
rA/USA/09	3529/MLS/92	0.0125084
rA/USA/09	Ventri/IND/10	0.0131339
rA/USA/09	CAHFS-785/USA/11	0.0139720
rA/USA/09	7741-SEGA/USA/12	0.0139720
rA/USA/09	89163/FRA/89	0.0139849
rA/USA/09	T09/NG/98	0.0139925
rA/USA/09	Bpop-03/POL/03	0.0139982
rA/USA/09	Cro-Ig/CRT/02	0.0140413
rB/USA/09	VarE/USA/94	0.0141430
rB/USA/09	Cu-1wt/GER/75	0.0141745
rB/USA/09	9109/USA/03	0.0141831
rB/USA/09	150128/4.1/ALG/15	0.0143616
rB/USA/09	150133/3.2/ALG/14	0.0143616
rA/USA/09	HuB-1/CHN/00	0.0164059
rB/USA/09	F52/70/UK/70	0.0164856
rA/USA/09	150128/4.1/ALG/15	0.0166436
rA/USA/09	150133/3.2/ALG/14	0.0166436

rB/USA/09	150124/1.1/ALG/16	0.0166679
rA/USA/09	Harbin-1/CHN/92	0.0186751
rA/USA/09	GZ/96/CHN/04	0.0186885
rB/USA/09	903/78/HNG/78	0.0188570
rB/USA/09	Gt/CHN/06	0.0188646
rB/USA/09	STC/USA/67	0.0188656
rB/USA/09	JD1/CHN/97	0.0188838
rB/USA/09	CEF94/NLD/73	0.0189106
rB/USA/09	HZ2/CHN/97	0.0189106
rA/USA/09	150124/1.1/ALG/16	0.0189499
rB/USA/09	150144/5.1/ALG/15	0.0190180
rB/USA/09	TN46/19/TUN/19	0.0195879
rA/USA/09	VarE/USA/94	0.0210685
rA/USA/09	Cu-1wt/GER/75	0.0211000
rA/USA/09	9109/USA/03	0.0211086
rA/USA/09	150144/5.1/ALG/15	0.0213001
rA/USA/09	TN46/19/TUN/19	0.0218699
rA/USA/09	F52/70/UK/70	0.0234111
rB/USA/09	B87/CHN/06	0.0235238
rA/USA/09	903/78/HNG/78	0.0257825
rA/USA/09	Gt/CHN/06	0.0257901
rA/USA/09	STC/USA/67	0.0257911
rA/USA/09	JD1/CHN/97	0.0258093
rA/USA/09	CEF94/NLD/73	0.0258361
rA/USA/09	HZ2/CHN/97	0.0258361
rB/USA/09	dIBDV/2202/URY/14	0.0269099
rA/USA/09	B87/CHN/06	0.0304493
rA/USA/09	dIBDV/2202/URY/14	0.0338354
rB/USA/09	OH/USA/82	0.1432187
rA/USA/09	OH/USA/82	0.1501442

rA was most related to rB (shown in bold black). rB was the closest to mb isolate (shown in bold red).

Table S14. Best fitting model selection for VP1 using Maximum Likelihood fits of 24 different nucleotide substitution models.

Model	BIC	AICc	lnL	I	G	R	FreqA	FreqT	FreqC	FreqG	A=>T	A=>C	A=>G	T=>A	T=>C	T=>G	C=>A	C=>T	C=>G	G=>A	G=>T	G=>C
**TN93+G***	**20434.79**	19768.70	-9813.29	n/a	0.1885	3.6516	0.2847	0.1908	0.2695	0.2550	0.02	0.03	0.13	0.03	0.3	0.03	0.03	0.22	0.03	0.14	0.02	0.03
TN93+G+I	20440.42	19764.95	-9810.42	0.5177	0.7745	3.7135	0.2847	0.1908	0.2695	0.2550	0.02	0.03	0.13	0.03	0.31	0.03	0.03	0.22	0.03	0.14	0.02	0.03
GTR+G+I	20440.67	19737.06	-9793.46	0.4147	0.5144	3.1849	0.2847	0.1908	0.2695	0.2550	0.04	0.03	0.13	0.05	0.28	0.02	0.03	0.2	0.02	0.15	0.01	0.02
GTR+G	20443.96	19749.73	-9800.80	n/a	0.1915	3.0444	0.2847	0.1908	0.2695	0.2550	0.04	0.03	0.13	0.05	0.28	0.02	0.03	0.2	0.03	0.15	0.01	0.03
HKY+G	20492.40	19835.69	-9847.79	n/a	0.1802	3.6461	0.2847	0.1908	0.2695	0.2550	0.02	0.03	0.2	0.03	0.21	0.03	0.03	0.15	0.03	0.22	0.02	0.03
K2+G	20499.57	19871.00	-9868.45	n/a	0.1861	3.4902	0.25	0.25	0.25	0.25	0.03	0.03	0.19	0.03	0.19	0.03	0.03	0.19	0.03	0.19	0.03	0.03
HKY+G+I	20505.34	19839.25	-9848.57	0.3231	0.3637	3.6937	0.2847	0.1908	0.2695	0.2550	0.02	0.03	0.2	0.03	0.21	0.03	0.03	0.15	0.03	0.22	0.02	0.03
K2+G+I	20512.62	19874.67	-9869.28	0.3938	0.4706	3.5332	0.25	0.25	0.25	0.25	0.03	0.03	0.19	0.03	0.19	0.03	0.03	0.19	0.03	0.19	0.03	0.03
T92+G	20516.69	19878.74	-9871.32	n/a	0.1873	3.4903	0.2378	0.2378	0.2622	0.2622	0.03	0.03	0.2	0.03	0.2	0.03	0.03	0.18	0.03	0.18	0.03	0.03
T92+G+I	20524.58	19877.25	-9869.57	0.3753	0.4429	3.5384	0.2378	0.2378	0.2622	0.2622	0.03	0.03	0.2	0.03	0.2	0.03	0.03	0.19	0.03	0.19	0.03	0.03
TN93+I	20813.32	20147.23	-10002.56	0.3818	n/a	3.2474	0.2847	0.1908	0.2695	0.2550	0.02	0.03	0.13	0.03	0.29	0.03	0.03	0.21	0.03	0.14	0.02	0.03
GTR+I	20824.86	20130.63	-9991.25	0.3818	n/a	2.6579	0.2847	0.1908	0.2695	0.2550	0.04	0.04	0.13	0.06	0.27	0.02	0.04	0.19	0.03	0.15	0.02	0.03
K2+I	20883.37	20254.80	-10060.35	0.3818	n/a	3.1595	0.25	0.25	0.25	0.25	0.03	0.03	0.19	0.03	0.19	0.03	0.03	0.19	0.03	0.19	0.03	0.03
T92+I	20895.62	20257.67	-10060.78	0.3818	n/a	3.1633	0.2378	0.2378	0.2622	0.2622	0.03	0.03	0.2	0.03	0.2	0.03	0.03	0.18	0.03	0.18	0.03	0.03
HKY+I	20897.70	20240.99	-10050.44	0.3818	n/a	3.2089	0.2847	0.1908	0.2695	0.2550	0.02	0.03	0.19	0.03	0.21	0.03	0.03	0.15	0.03	0.22	0.02	0.03
TN93	21251.00	20594.29	-10227.09	n/a	n/a	3.0772	0.2847	0.1908	0.2695	0.2550	0.02	0.03	0.13	0.03	0.28	0.03	0.03	0.2	0.03	0.15	0.02	0.03
GTR	21261.55	20576.70	-10215.29	n/a	n/a	2.5672	0.2847	0.1908	0.2695	0.2550	0.04	0.04	0.14	0.06	0.26	0.02	0.04	0.18	0.03	0.15	0.02	0.03
K2	21310.96	20691.77	-10279.83	n/a	n/a	3.0618	0.25	0.25	0.25	0.25	0.03	0.03	0.19	0.03	0.19	0.03	0.03	0.19	0.03	0.19	0.03	0.03
T92	21323.20	20694.63	-10280.26	n/a	n/a	3.0644	0.2378	0.2378	0.2622	0.2622	0.03	0.03	0.2	0.03	0.2	0.03	0.03	0.18	0.03	0.18	0.03	0.03
HKY	21336.33	20689.00	-10275.45	n/a	n/a	3.0675	0.2847	0.1908	0.2695	0.2550	0.02	0.03	0.19	0.03	0.2	0.03	0.03	0.14	0.03	0.21	0.02	0.03
JC+G	21362.03	20742.84	-10305.37	n/a	0.2020	0.5	0.25	0.25	0.25	0.25	0.08	0.08	0.08	0.08	0.08	0.08	0.08	0.08	0.08	0.08	0.08	0.08
JC+G+I	21367.96	20739.39	-10302.65	0.1153	0.2533	0.5	0.25	0.25	0.25	0.25	0.08	0.08	0.08	0.08	0.08	0.08	0.08	0.08	0.08	0.08	0.08	0.08
JC+I	21689.32	21070.13	-10469.01	0.3882	n/a	0.5	0.25	0.25	0.25	0.25	0.08	0.08	0.08	0.08	0.08	0.08	0.08	0.08	0.08	0.08	0.08	0.08
JC	22108.45	21498.64	-10684.27	n/a	n/a	0.5	0.25	0.25	0.25	0.25	0.08	0.08	0.08	0.08	0.08	0.08	0.08	0.08	0.08	0.08	0.08	0.08

*Model with the lowest BIC scores (Bayesian Information Criterion) is considered to describe the substitution pattern best (shown in bold red). For each model. A total of 2640 positions existed in the final dataset. Abbreviations: TN93: Tamura-Nei; K2: Kimura 2-parameter; GTR: General Time Reversible; HKY: Hasegawa-Kishino-Yano; T92: Tamura 3-parameter; JC: Jukes-Cantor.

Table S15. Best fitting model selection for mature VP2 using Maximum Likelihood fits of 24 different nucleotide substitution models.

Model	BIC	AICc	lnL	I	G	R	FreqA	FreqT	FreqC	FreqG	A=>T	A=>C	A=>G	T=>A	T=>C	T=>G	C=>A	C=>T	C=>G	G=>A	G=>T	G=>C
**GTR+G***	**10915.63**	10092.64	-4954.17	n/a	0.2049	3.3050	0.2728	0.2069	0.2697	0.2507	0.04	0.05	0.14	0.05	0.28	0.02	0.05	0.21	0.01	0.15	0.02	0.01
K2+G	10917.47	10157.08	-4993.41	n/a	0.2014	3.2629	0.25	0.25	0.25	0.25	0.03	0.03	0.19	0.03	0.19	0.03	0.03	0.19	0.03	0.19	0.03	0.03
TN93+G	10922.15	10125.99	-4973.85	n/a	0.2005	3.3496	0.2728	0.2069	0.2697	0.2507	0.02	0.03	0.14	0.03	0.28	0.03	0.03	0.21	0.03	0.15	0.02	0.03
K2+G+I	10925.65	10156.32	-4992.03	0.3684	0.4413	3.2564	0.25	0.25	0.25	0.25	0.03	0.03	0.19	0.03	0.19	0.03	0.03	0.19	0.03	0.19	0.03	0.03
GTR+G+I	10926.20	10094.28	-4953.98	0.3892	0.4939	3.2894	0.2728	0.2069	0.2697	0.2507	0.04	0.05	0.14	0.05	0.28	0.02	0.05	0.21	0.01	0.15	0.02	0.01
T92+G	10928.39	10159.05	-4993.40	n/a	0.2011	3.2720	0.2398	0.2398	0.2602	0.2602	0.03	0.03	0.2	0.03	0.2	0.03	0.03	0.18	0.03	0.18	0.03	0.03
TN93+G+I	10930.16	10125.05	-4972.38	0.3953	0.4941	3.3163	0.2728	0.2069	0.2697	0.2507	0.02	0.03	0.14	0.03	0.28	0.03	0.03	0.21	0.03	0.15	0.02	0.03
HKY+G	10931.23	10144.01	-4983.87	n/a	0.1978	3.3494	0.2728	0.2069	0.2697	0.2507	0.02	0.03	0.19	0.03	0.21	0.03	0.03	0.16	0.03	0.21	0.02	0.03
T92+G+I	10934.13	10155.85	-4990.79	0.3700	0.4397	3.2768	0.2398	0.2398	0.2602	0.2602	0.03	0.03	0.2	0.03	0.2	0.03	0.03	0.18	0.03	0.18	0.03	0.03
HKY+G+I	10941.14	10144.98	-4983.35	0.3727	0.4433	3.3270	0.2728	0.2069	0.2697	0.2507	0.02	0.03	0.19	0.03	0.21	0.03	0.03	0.16	0.03	0.21	0.02	0.03
GTR+I	10941.40	10118.41	-4967.05	0.6629	n/a	3.2898	0.2728	0.2069	0.2697	0.2507	0.04	0.04	0.13	0.05	0.28	0.02	0.05	0.21	0.01	0.15	0.02	0.01
K2+I	10944.12	10183.74	-5006.74	0.6709	n/a	3.2134	0.25	0.25	0.25	0.25	0.03	0.03	0.19	0.03	0.19	0.03	0.03	0.19	0.03	0.19	0.03	0.03
TN93+I	10946.77	10150.61	-4986.16	0.6670	n/a	3.3104	0.2728	0.2069	0.2697	0.2507	0.02	0.03	0.14	0.03	0.27	0.03	0.03	0.21	0.03	0.15	0.02	0.03
T92+I	10955.02	10185.68	-5006.71	0.6705	n/a	3.2141	0.2398	0.2398	0.2602	0.2602	0.03	0.03	0.2	0.03	0.2	0.03	0.03	0.18	0.03	0.18	0.03	0.03
HKY+I	10958.21	10170.99	-4997.36	0.6717	n/a	3.2887	0.2728	0.2069	0.2697	0.2507	0.02	0.03	0.19	0.03	0.21	0.03	0.03	0.16	0.03	0.21	0.02	0.03
JC+G	11263.15	10511.71	-5171.73	n/a	0.2383	0.5	0.25	0.25	0.25	0.25	0.08	0.08	0.08	0.08	0.08	0.08	0.08	0.08	0.08	0.08	0.08	0.08
JC+G+I	11269.55	10509.16	-5169.45	0.3595	0.5107	0.5	0.25	0.25	0.25	0.25	0.08	0.08	0.08	0.08	0.08	0.08	0.08	0.08	0.08	0.08	0.08	0.08
JC+I	11288.00	10536.56	-5184.15	0.6572	n/a	0.5	0.25	0.25	0.25	0.25	0.08	0.08	0.08	0.08	0.08	0.08	0.08	0.08	0.08	0.08	0.08	0.08
K2	11290.36	10538.91	-5185.33	n/a	n/a	2.3601	0.25	0.25	0.25	0.25	0.04	0.04	0.18	0.04	0.18	0.04	0.04	0.18	0.04	0.18	0.04	0.04
TN93	11292.69	10505.47	-5164.60	n/a	n/a	2.3648	0.2728	0.2069	0.2697	0.2507	0.03	0.04	0.13	0.04	0.24	0.04	0.04	0.19	0.04	0.14	0.03	0.04
T92	11300.38	10539.99	-5184.86	n/a	n/a	2.3627	0.2398	0.2398	0.2602	0.2602	0.04	0.04	0.18	0.04	0.18	0.04	0.04	0.17	0.04	0.17	0.04	0.04
GTR	11307.21	10493.17	-5155.44	n/a	n/a	1.9603	0.2728	0.2069	0.2697	0.2507	0.05	0.06	0.13	0.06	0.22	0.02	0.06	0.17	0.04	0.15	0.02	0.04
HKY	11311.47	10533.19	-5179.46	n/a	n/a	2.3622	0.2728	0.2069	0.2697	0.2507	0.03	0.04	0.18	0.04	0.19	0.04	0.04	0.15	0.04	0.19	0.03	0.04
JC	11598.05	10855.54	-5344.65	n/a	n/a	0.5	0.25	0.25	0.25	0.25	0.08	0.08	0.08	0.08	0.08	0.08	0.08	0.08	0.08	0.08	0.08	0.08

*Model with the lowest BIC scores depicts the substitution pattern best (shown in bold red). For each model. A total of 1326 positions existed in the final dataset. Abbreviations: GTR: General Time Reversible; TN93: Tamura-Nei; K2: Kimura 2-parameter; HKY: Hasegawa-Kishino-Yano; T92: Tamura 3-parameter; JC: Jukes-Cantor.

Table S16. Best fitting model selection for mature VP3 using Maximum Likelihood fits of 24 different nucleotide substitution models.

Model	BIC	AICc	lnL	I	G	R	FreqA	FreqT	FreqC	FreqG	A=>T	A=>C	A=>G	T=>A	T=>C	T=>G	C=>A	C=>T	C=>G	G=>A	G=>T	G=>C
**TN93+G***	**6491.22**	5743.06	-2782.29	n/a	0.2263	3.6849	0.2947	0.1479	0.2805	0.2769	0.01	0.03	0.11	0.03	0.38	0.03	0.03	0.2	0.03	0.12	0.01	0.03
TN93+G+I	6496.04	5739.48	-2779.49	0.3938	0.5143	3.7681	0.2947	0.1479	0.2805	0.2769	0.01	0.03	0.11	0.03	0.38	0.03	0.03	0.2	0.03	0.12	0.01	0.03
GTR+G	6514.50	5741.14	-2778.31	n/a	0.2404	2.8539	0.2947	0.1479	0.2805	0.2769	0.03	0.03	0.12	0.07	0.33	0.02	0.03	0.17	0.03	0.13	0.01	0.03
GTR+G+I	6516.33	5734.57	-2774.02	0.3853	0.5470	3.0532	0.2947	0.1479	0.2805	0.2769	0.03	0.03	0.12	0.06	0.34	0.02	0.03	0.18	0.02	0.12	0.01	0.02
TN93+I	6518.70	5770.54	-2796.03	0.6561	n/a	3.3994	0.2947	0.1479	0.2805	0.2769	0.02	0.03	0.12	0.03	0.35	0.03	0.03	0.19	0.03	0.13	0.02	0.03
K2+G	6522.49	5807.94	-2818.75	n/a	0.2293	3.3675	0.25	0.25	0.25	0.25	0.03	0.03	0.19	0.03	0.19	0.03	0.03	0.19	0.03	0.19	0.03	0.03
HKY+G	6524.88	5785.13	-2804.33	n/a	0.2204	3.6425	0.2947	0.1479	0.2805	0.2769	0.02	0.03	0.22	0.03	0.22	0.03	0.03	0.12	0.03	0.23	0.02	0.03
T92+G	6525.64	5802.69	-2815.12	n/a	0.2353	3.3392	0.2213	0.2213	0.2787	0.2787	0.03	0.03	0.22	0.03	0.22	0.03	0.03	0.17	0.03	0.17	0.03	0.03
K2+G+I	6526.72	5803.77	-2815.66	0.4303	0.5977	3.4353	0.25	0.25	0.25	0.25	0.03	0.03	0.19	0.03	0.19	0.03	0.03	0.19	0.03	0.19	0.03	0.03
HKY+G+I	6528.79	5780.63	-2801.07	0.4344	0.5719	3.7225	0.2947	0.1479	0.2805	0.2769	0.02	0.03	0.22	0.03	0.22	0.03	0.03	0.12	0.03	0.23	0.02	0.03
T92+G+I	6530.42	5799.06	-2812.30	0.4242	0.6026	3.4050	0.2213	0.2213	0.2787	0.2787	0.02	0.03	0.22	0.02	0.22	0.03	0.02	0.17	0.03	0.17	0.02	0.03
K2+I	6544.23	5829.68	-2829.62	0.6694	n/a	3.2597	0.25	0.25	0.25	0.25	0.03	0.03	0.19	0.03	0.19	0.03	0.03	0.19	0.03	0.19	0.03	0.03
GTR+I	6545.05	5771.69	-2793.59	0.6523	n/a	2.6290	0.2947	0.1479	0.2805	0.2769	0.03	0.03	0.13	0.07	0.31	0.02	0.03	0.16	0.03	0.14	0.01	0.03
T92+I	6546.01	5823.06	-2825.30	0.6657	n/a	3.2283	0.2213	0.2213	0.2787	0.2787	0.03	0.03	0.21	0.03	0.21	0.03	0.03	0.17	0.03	0.17	0.03	0.03
HKY+I	6547.10	5807.34	-2815.43	0.6714	n/a	3.4558	0.2947	0.1479	0.2805	0.2769	0.02	0.03	0.21	0.03	0.22	0.03	0.03	0.11	0.03	0.23	0.02	0.03
TN93	6686.90	5947.14	-2885.33	n/a	n/a	2.8157	0.2947	0.1479	0.2805	0.2769	0.02	0.03	0.13	0.04	0.32	0.03	0.04	0.17	0.03	0.14	0.02	0.03
GTR	6703.81	5938.85	-2878.17	n/a	n/a	2.2073	0.2947	0.1479	0.2805	0.2769	0.04	0.04	0.13	0.07	0.28	0.02	0.05	0.15	0.03	0.14	0.01	0.03
K2	6708.63	6002.48	-2917.03	n/a	n/a	2.7939	0.25	0.25	0.25	0.25	0.03	0.03	0.18	0.03	0.18	0.03	0.03	0.18	0.03	0.18	0.03	0.03
T92	6709.82	5995.27	-2912.41	n/a	n/a	2.7977	0.2213	0.2213	0.2787	0.2787	0.03	0.04	0.21	0.03	0.21	0.04	0.03	0.16	0.04	0.16	0.03	0.04
HKY	6721.52	5990.17	-2907.85	n/a	n/a	2.8002	0.2947	0.1479	0.2805	0.2769	0.02	0.04	0.2	0.04	0.21	0.04	0.04	0.11	0.04	0.22	0.02	0.04
JC+G	6736.67	6030.52	-2931.04	n/a	0.2529	0.5	0.25	0.25	0.25	0.25	0.08	0.08	0.08	0.08	0.08	0.08	0.08	0.08	0.08	0.08	0.08	0.08
JC+G+I	6745.26	6030.71	-2930.13	0.4046	0.6420	0.5	0.25	0.25	0.25	0.25	0.08	0.08	0.08	0.08	0.08	0.08	0.08	0.08	0.08	0.08	0.08	0.08
JC+I	6753.06	6046.90	-2939.24	0.6627	n/a	0.5	0.25	0.25	0.25	0.25	0.08	0.08	0.08	0.08	0.08	0.08	0.08	0.08	0.08	0.08	0.08	0.08
JC	6911.39	6213.64	-3023.61	n/a	n/a	0.5	0.25	0.25	0.25	0.25	0.08	0.08	0.08	0.08	0.08	0.08	0.08	0.08	0.08	0.08	0.08	0.08

*Model with the lowest BIC scores (Bayesian Information Criterion) is regarded as the best describing the substitution pattern (shown in bold red). For each model. A total of 774 positions existed in the final dataset. Abbreviations: TN93: Tamura-Nei; K2: Kimura 2-parameter; GTR: General Time Reversible; HKY: Hasegawa-Kishino-Yano; T92: Tamura 3-parameter; JC: Jukes-Cantor.

Table S17. Best fitting model selection for mature VP4 using Maximum Likelihood fits of 24 different nucleotide substitution models.

Model	BIC	AICc	lnL	I	G	R	Freq A	Freq T	Freq C	Freq G	A=>T	A=>C	A=>G	T=>A	T=>C	T=>G	C=>A	C=>T	C=>G	G=>A	G=>T	G=>C
**K2+G***	**6838.48**	6129.04	-2979.29	n/a	0.2404	2.8693	0.25	0.25	0.25	0.25	0.03	0.03	0.19	0.03	0.19	0.03	0.03	0.19	0.03	0.19	0.03	0.03
K2+G+I	6848.43	6130.64	-2979.08	0.2047	0.3593	2.8788	0.25	0.25	0.25	0.25	0.03	0.03	0.19	0.03	0.19	0.03	0.03	0.19	0.03	0.19	0.03	0.03
T92+G	6848.64	6130.85	-2979.19	n/a	0.2434	2.8573	0.2313	0.2313	0.2687	0.2687	0.03	0.03	0.2	0.03	0.2	0.03	0.03	0.17	0.03	0.17	0.03	0.03
TN93+G	6852.47	6109.66	-2965.58	n/a	0.2320	3.0623	0.2464	0.2162	0.2872	0.2502	0.03	0.04	0.12	0.03	0.3	0.03	0.03	0.22	0.03	0.12	0.03	0.04
K2+I	6853.34	6143.90	-2986.71	0.6423	n/a	2.7934	0.25	0.25	0.25	0.25	0.03	0.03	0.18	0.03	0.18	0.03	0.03	0.18	0.03	0.18	0.03	0.03
T92+G+I	6858.49	6132.37	-2978.94	0.2160	0.3751	2.8548	0.2313	0.2313	0.2687	0.2687	0.03	0.03	0.2	0.03	0.2	0.03	0.03	0.17	0.03	0.17	0.03	0.03
GTR+G	6859.93	6092.11	-2953.78	n/a	0.2331	3.0823	0.2464	0.2162	0.2872	0.2502	0.04	0.04	0.12	0.05	0.3	0.04	0.03	0.22	0.01	0.11	0.03	0.01
HKY+G	6861.21	6126.74	-2975.12	n/a	0.2372	2.9131	0.2464	0.2162	0.2872	0.2502	0.03	0.04	0.19	0.03	0.21	0.03	0.03	0.16	0.03	0.18	0.03	0.04
TN93+G+I	6862.33	6111.18	-2965.33	0	0.2317	3.0651	0.2464	0.2162	0.2872	0.2502	0.03	0.04	0.12	0.03	0.3	0.03	0.03	0.22	0.03	0.12	0.03	0.04
T92+I	6863.38	6145.59	-2986.56	0.6404	n/a	2.7812	0.2313	0.2313	0.2687	0.2687	0.03	0.04	0.2	0.03	0.2	0.04	0.03	0.17	0.04	0.17	0.03	0.04
TN93+I	6867.22	6124.42	-2972.95	0.6392	n/a	3.0076	0.2464	0.2162	0.2872	0.2502	0.03	0.04	0.12	0.03	0.29	0.03	0.03	0.22	0.03	0.12	0.03	0.04
GTR+G+I	6870.29	6094.12	-2953.78	0.0000	0.2325	3.0878	0.2464	0.2162	0.2872	0.2502	0.04	0.04	0.12	0.05	0.3	0.04	0.03	0.22	0.01	0.11	0.03	0.01
HKY+G+I	6871.23	6128.42	-2974.96	0.1794	0.3332	2.9318	0.2464	0.2162	0.2872	0.2502	0.03	0.04	0.19	0.03	0.21	0.03	0.03	0.16	0.03	0.18	0.03	0.04
HKY+I	6876.08	6141.61	-2982.56	0.6440	n/a	2.8664	0.2464	0.2162	0.2872	0.2502	0.03	0.04	0.19	0.03	0.21	0.03	0.03	0.16	0.03	0.18	0.03	0.04
GTR+I	6876.76	6108.94	-2962.19	0.6359	n/a	3.0726	0.2464	0.2162	0.2872	0.2502	0.04	0.04	0.12	0.05	0.3	0.04	0.04	0.22	0.01	0.12	0.03	0.01
JC+G	7040.04	6338.94	-3085.24	n/a	0.2800	0.5	0.25	0.25	0.25	0.25	0.08	0.08	0.08	0.08	0.08	0.08	0.08	0.08	0.08	0.08	0.08	0.08
K2	7045.55	6344.45	-3087.99	n/a	n/a	2.2651	0.25	0.25	0.25	0.25	0.04	0.04	0.17	0.04	0.17	0.04	0.04	0.17	0.04	0.17	0.04	0.04
JC+G+I	7049.88	6340.43	-3084.98	0.2340	0.4575	0.5	0.25	0.25	0.25	0.25	0.08	0.08	0.08	0.08	0.08	0.08	0.08	0.08	0.08	0.08	0.08	0.08
JC+I	7052.42	6351.32	-3091.43	0.6288	n/a	0.5	0.25	0.25	0.25	0.25	0.08	0.08	0.08	0.08	0.08	0.08	0.08	0.08	0.08	0.08	0.08	0.08
T92	7053.21	6343.76	-3086.65	n/a	n/a	2.2690	0.2313	0.2313	0.2687	0.2687	0.04	0.04	0.19	0.04	0.19	0.04	0.04	0.16	0.04	0.16	0.04	0.04
TN93	7064.22	6329.76	-3076.63	n/a	n/a	2.2807	0.2464	0.2162	0.2872	0.2502	0.03	0.04	0.13	0.04	0.25	0.04	0.04	0.19	0.04	0.13	0.03	0.04
HKY	7072.47	6346.35	-3085.93	n/a	n/a	2.2710	0.2464	0.2162	0.2872	0.2502	0.03	0.04	0.17	0.04	0.2	0.04	0.04	0.15	0.04	0.17	0.03	0.04
GTR	7087.78	6328.29	-3072.88	n/a	n/a	1.8552	0.2464	0.2162	0.2872	0.2502	0.05	0.06	0.13	0.06	0.23	0.03	0.05	0.17	0.04	0.13	0.03	0.04
JC	7225.59	6532.83	-3183.19	n/a	n/a	0.5	0.25	0.25	0.25	0.25	0.08	0.08	0.08	0.08	0.08	0.08	0.08	0.08	0.08	0.08	0.08	0.08

*Model with the lowest BIC scores (Bayesian Information Criterion) is considered to describe the substitution pattern best (shown in bold red). For each model. A total of 730 positions existed in the final dataset. Abbreviations: K2: Kimura 2-parameter; TN93: Tamura-Nei; GTR: General Time Reversible; HKY: Hasegawa-Kishino-Yano; T92: Tamura 3-parameter; JC: Jukes-Cantor.

Table S18. Best fitting model selection for mature VP5 using Maximum Likelihood fits of 24 different nucleotide substitution models.

Model	BIC	AICc	lnL	I	G	R	Freq A	Freq T	Freq C	Freq G	A=>T	A=>C	A=>G	T=>A	T=>C	T=>G	C=>A	C=>T	C=>G	G=>A	G=>T	G=>C
**K2+G***	**3123.76**	2456.48	-1142.86	n/a	0.1777	2.0439	0.25	0.25	0.25	0.25	0.04	0.04	0.17	0.04	0.17	0.04	0.04	0.17	0.04	0.17	0.04	0.04
K2+G+I	3131.17	2456.04	-1141.63	0.5461	0.6866	2.1155	0.25	0.25	0.25	0.25	0.04	0.04	0.17	0.04	0.17	0.04	0.04	0.17	0.04	0.17	0.04	0.04
T92+G	3132.03	2456.90	-1142.06	n/a	0.1816	2.0326	0.2388	0.2388	0.2612	0.2612	0.04	0.04	0.18	0.04	0.18	0.04	0.04	0.16	0.04	0.16	0.04	0.04
T92+G+I	3139.59	2456.62	-1140.91	0.5437	0.6808	2.1262	0.2388	0.2388	0.2612	0.2612	0.04	0.04	0.18	0.04	0.18	0.04	0.04	0.16	0.04	0.16	0.04	0.04
K2+I	3140.60	2473.31	-1151.27	0.4256	n/a	1.8506	0.25	0.25	0.25	0.25	0.04	0.04	0.16	0.04	0.16	0.04	0.04	0.16	0.04	0.16	0.04	0.04
HKY+G	3146.55	2455.74	-1139.46	n/a	0.1728	2.0715	0.2651	0.2125	0.2772	0.2451	0.03	0.04	0.17	0.04	0.19	0.04	0.04	0.14	0.04	0.18	0.03	0.04
T92+I	3148.54	2473.42	-1150.32	0.4256	n/a	1.8490	0.2388	0.2388	0.2612	0.2612	0.04	0.05	0.17	0.04	0.17	0.05	0.04	0.16	0.05	0.16	0.04	0.05
K2	3149.35	2489.91	-1160.58	n/a	n/a	1.7927	0.25	0.25	0.25	0.25	0.04	0.04	0.16	0.04	0.16	0.04	0.04	0.16	0.04	0.16	0.04	0.04
JC+G	3149.77	2490.33	-1160.79	n/a	0.1972	0.5	0.25	0.25	0.25	0.25	0.08	0.08	0.08	0.08	0.08	0.08	0.08	0.08	0.08	0.08	0.08	0.08
TN93+G	3153.68	2455.03	-1138.09	n/a	0.1772	2.0927	0.2651	0.2125	0.2772	0.2451	0.03	0.04	0.12	0.04	0.24	0.04	0.04	0.18	0.04	0.13	0.03	0.04
HKY+G+I	3156.63	2457.99	-1139.57	0.5589	0.6624	2.6127	0.2651	0.2125	0.2772	0.2451	0.03	0.04	0.18	0.04	0.2	0.03	0.04	0.15	0.03	0.19	0.03	0.04
T92	3157.19	2489.90	-1159.57	n/a	n/a	1.7933	0.2388	0.2388	0.2612	0.2612	0.04	0.05	0.17	0.04	0.17	0.05	0.04	0.15	0.05	0.15	0.04	0.05
JC+G+I	3157.89	2490.60	-1159.92	0.5046	0.6782	0.5	0.25	0.25	0.25	0.25	0.08	0.08	0.08	0.08	0.08	0.08	0.08	0.08	0.08	0.08	0.08	0.08
TN93+G+I	3161.25	2454.76	-1136.95	0.5322	0.6465	2.1571	0.2651	0.2125	0.2772	0.2451	0.03	0.04	0.12	0.04	0.24	0.04	0.04	0.19	0.04	0.13	0.03	0.04
HKY+I	3163.84	2473.04	-1148.11	0.4256	n/a	1.8572	0.2651	0.2125	0.2772	0.2451	0.04	0.05	0.16	0.05	0.18	0.04	0.05	0.14	0.04	0.17	0.04	0.05
JC+I	3164.90	2505.45	-1168.35	0.4256	n/a	0.5	0.25	0.25	0.25	0.25	0.08	0.08	0.08	0.08	0.08	0.08	0.08	0.08	0.08	0.08	0.08	0.08
TN93+I	3170.72	2472.07	-1146.62	0.4256	n/a	1.8692	0.2651	0.2125	0.2772	0.2451	0.04	0.05	0.12	0.05	0.23	0.04	0.05	0.17	0.04	0.13	0.04	0.05
HKY	3172.80	2489.83	-1157.51	n/a	n/a	1.7944	0.2651	0.2125	0.2772	0.2451	0.04	0.05	0.16	0.05	0.18	0.04	0.05	0.14	0.04	0.17	0.04	0.05
JC	3172.95	2521.35	-1177.31	n/a	n/a	0.5	0.25	0.25	0.25	0.25	0.08	0.08	0.08	0.08	0.08	0.08	0.08	0.08	0.08	0.08	0.08	0.08
TN93	3179.69	2488.88	-1156.03	n/a	n/a	1.8004	0.2651	0.2125	0.2772	0.2451	0.04	0.05	0.12	0.05	0.22	0.04	0.05	0.17	0.04	0.13	0.04	0.05
GTR+G	3179.79	2457.62	-1136.36	n/a	0.1723	2.1684	0.2651	0.2125	0.2772	0.2451	0.04	0.06	0.12	0.05	0.24	0.05	0.06	0.19	0.01	0.13	0.04	0.01
GTR+G+I	3187.86	2457.85	-1135.47	0.5578	0.5991	2.4218	0.2651	0.2125	0.2772	0.2451	0.04	0.06	0.13	0.05	0.25	0.05	0.05	0.19	0	0.14	0.04	0
GTR+I	3196.93	2474.76	-1144.93	0.4256	n/a	1.8817	0.2651	0.2125	0.2772	0.2451	0.04	0.07	0.12	0.05	0.23	0.04	0.06	0.17	0.02	0.13	0.04	0.02
GTR	3208.38	2494.05	-1155.58	n/a	n/a	1.4934	0.2651	0.2125	0.2772	0.2451	0.04	0.07	0.12	0.05	0.2	0.04	0.06	0.15	0.04	0.13	0.03	0.05

*Model with the lowest BIC scores (Bayesian Information Criterion) is considered to describe the substitution pattern best (shown in bold red). For each model. A total of 450 positions existed in the final dataset. Abbreviations: K2: Kimura 2-parameter; TN93: Tamura-Nei; GTR: General Time Reversible; HKY: Hasegawa-Kishino-Yano; T92: Tamura 3-parameter; JC: Jukes-Cantor.
